# High expression of ID family and IGJ genes signature as predictor of low induction treatment response and worst survival in adult Hispanic patients with B-acute lymphoblastic leukemia

**DOI:** 10.1186/s13046-016-0333-z

**Published:** 2016-04-05

**Authors:** Nataly Cruz-Rodriguez, Alba L. Combita, Leonardo J. Enciso, Sandra M. Quijano, Paula L. Pinzon, Olga C. Lozano, Juan S. Castillo, Li Li, Jose Bareño, Claudia Cardozo, Julio Solano, Maria V. Herrera, Jennifer Cudris, Jovanny Zabaleta

**Affiliations:** Programa de Investigación e Innovación en Leucemias Agudas y Crónicas (PILAC), Instituto Nacional de Cancerología, Bogotá, Colombia; Group of Investigation in Biology of Cancer, Instituto Nacional de Cancerología, Calle 1 # 9-85, Bogotá, Colombia; Programa de Doctorado en Ciencias Biológicas, Pontificia Universidad Javeriana, Bogotá, Colombia; Grupo de Inmunobiología y Biología Celular, Departamento de Microbiología, Facultad de Ciencias, Pontificia Universidad Javeriana, Bogotá, Colombia; Hospital Universitario San Ignacio, Bogotá, Colombia; Grupo de Hemato Oncología, Instituto Nacional de Cancerología, Bogotá, Colombia; Universidad CES, Medellin, Colombia; Facultad de Medicina, Universidad Nacional de Colombia, Bogotá, Colombia; Stanley S. Scott Cancer Center, Center Louisiana State University Health Sciences Center Louisiana Cancer Research Center, 1700 Tulane Ave, Room 909, New Orleans, LA USA; Department of Pediatrics, Center Louisiana State University Health Sciences Center Louisiana Cancer Research Center, 1700 Tulane Ave, Room 909, New Orleans, LA USA

**Keywords:** Acute lymphoblastic leukemia, Gene expression profile, Complete remission, Minimal residual disease, Translational research

## Abstract

**Background:**

B-Acute lymphoblastic leukemia (B-ALL) represents a hematologic malignancy with poor clinical outcome and low survival rates in adult patients. Remission rates in Hispanic population are almost 30 % lower and Overall Survival (OS) nearly two years inferior than those reported in other ethnic groups. Only 61 % of Colombian adult patients with ALL achieve complete remission (CR), median overall survival is 11.3 months and event-free survival (EFS) is 7.34 months. Identification of prognostic factors is crucial for the application of proper treatment strategies and subsequently for successful outcome. Our goal was to identify a gene expression signature that might correlate with response to therapy and evaluate the utility of these as prognostic tool in hispanic patients.

**Methods:**

We included 43 adult patients newly diagnosed with B-ALL. We used microarray analysis in order to identify genes that distinguish poor from good response to treatment using differential gene expression analysis. The expression profile was validated by real-time PCR (RT-PCT).

**Results:**

We identified 442 differentially expressed genes between responders and non-responders to induction treatment. Hierarchical analysis according to the expression of a 7-gene signature revealed 2 subsets of patients that differed in their clinical characteristics and outcome.

**Conclusions:**

Our study suggests that response to induction treatment and clinical outcome of Hispanic patients can be predicted from the onset of the disease and that gene expression profiles can be used to stratify patient risk adequately and accurately. The present study represents the first that shows the gene expression profiling of B-ALL Colombian adults and its relevance for stratification in the early course of disease.

**Electronic supplementary material:**

The online version of this article (doi:10.1186/s13046-016-0333-z) contains supplementary material, which is available to authorized users.

## Background

Acute lymphoblastic leukemia (ALL) is a hematologic malignancy involving the bone marrow and other organs due to uncontrolled proliferation of lymphoblasts that are characterized by a progressive loss of the ability of differentiation [1]. Although in Colombia ALL is not one of the most common types of cancer, it represents a public health problem that requires priority attention because its incidence and mortality increase annually [2, 3]. ALL is most common in children under 15 years old than in adults; however, in adults the disease has a more aggressive behavior than in children [4, 5]. Currently about 90 % of individuals younger than 15 years achieve complete remission and 70 % are cured of the disease [4], while in adults, only 75 % reach complete remission and disease free survival (DFS) does not exceed 30 % [6–8].

Although intensive chemotherapeutic schemes have been implemented in adults for more than a decade in the United States and other countries [9–14] and have led to higher rates of durable remissions in Colombia these schemes have shown disappointing results with a median OS of less than 11.3 months, EFS of only 7.34 months, and only 61 % achieve CR [15]. Interestingly, other Latin American studies report similar worst survival when implementing these same regimens [16, 17]. Therefore, it is interesting to explore the underlying molecular characteristics of the disease in Colombian patients.

Identification of prognostic factors in patients with ALL is crucial for the proper planning of treatment strategies and the optimal results of therapy. Currently, in order to risk -stratify patients with B-ALL, several variables are considered: 1) demographic characteristics such as age and gender; 2) leukocyte count at diagnosis of the disease and infiltration of tumor cells in the central nervous system; 3) biological characteristics of tumoral cells including immunophenotype, cytogenetic, chromosomal translocations; and 4) Responsiveness to treatment through detection of (MRD) residual disease using highly sensitive techniques [18, 19]. Genetic alterations that give rise to chromosomal rearrangements together with age (younger or older than 30 years) and white blood cell count (WBCC) (more or less than 30.000/uL) strongly influence the survival of patients with ALL [19, 20]. These 3 variables represent the factors that best determine the prognosis at diagnosis of B-ALL adult patients. However, approximately 50 % of adults with B-ALL do not have chromosomal rearrangements and therefore are classified as standard risk group [21]. It has been reported that this group of patients is particularly heterogeneous according to the OS and DFS and that a large number of them present treatment failure, relapse and death [22]. Therefore, there is a need to find new genetic markers that can be detected at diagnosis and at initial treatment stages in order to increase the accuracy of risk stratification and treatment allocation and also to minimize both, under and overtreatment.

In this context, determining the gene expression profile of thousands of genes simultaneously provides an approximation that enables a better exploration of the mechanisms of transformation and behavior of malignant cells. Since the development of techniques for the evaluation of gene expression like cDNA microarrays, great advances have been made in diagnostic, classification and prognosis of cancer in both hematological malignancies and solid tumors [23–30]. In hematological neoplasms the study of genes expressed in the disease versus normal tissue counterpart has elucidated the mechanisms of pathology, and identified potential points of therapeutic intervention [31]. Several reports have shown the ability of gene expression profiles to discriminate between different types of leukemias, including different subsets of acute lymphoblastic leukemia, as well as different disease outcomes in pediatric ALL groups. However, research in disease prognosis based on gene expression profiles specifically in B-ALL affecting adult patients is poorly explored [26, 27, 32].

The present study compares, for the first time, the gene expression profiles in diagnostic samples from B-ALL Colombian adult patients responding or not to induction therapy. This data represents a potential stratification tool that reflects the genomic characteristics of Colombian population.

## Methods

### Patients and samples

All patients were recruited at the Colombian National Cancer Institute and Hospital Universitario San Ignacio both in Bogota, Colombia. Forty-three samples from adult patients diagnosed with precursor B-ALL were analyzed in this study (41 bone marrow (BM) and 2 peripheral blood (PB) samples obtained at diagnosis). Three normal BM samples were used as control. The diagnosis of B-ALL was based on morphologic evaluation of bone marrow aspirate to determine the presence of blasts, immunophenotyping and cytogenetic analysis of peripheral blood (PB) or BM. This study was approved by the Institutional Review Boards and Ethics Committees of both, Colombian National Cancer Institute and Hospital Universitario San Ignacio. Participation was voluntary, and written informed consent was obtained.

### Flow cytometry

Immunophenotype analysis was performed using the panel of antibodies recommended and standardized by the European consortium Euroflow [33]. Lymphoblasts were gated combining 8-color fluorescence for identification and phenotypic characterization of B-ALL. Antigens tested included surface CD20, CD58, CD66, CD10, CD38, SmIgk, CD33, SIgM, CD117, IgM Lambda, CD9, CD13, CD22, CD24; Cytoplasmic IgMKappa, NuTdT; In addition to CD45, CD19 and CD34 as backbone markers. The 19 markers panel was evaluated in all patients to confirm the diagnosis and after chemotherapy induction treatment to establish the Minimal Residual Disease.

### Isolation of leukemia cells

Preparation of mononuclear cell suspensions from diagnostic BM aspirates or PB were made by density-gradient centrifugation (Lympho prep, Lonza) within 24 hours after sample obtaining. The blast population was separated with magnetic microbeads coated with either anti-CD19 or anti-CD34 antibodies followed by column enrichment using standard procedures and MACS (Miltenyi, Bergisch Gladbach, Germany). Purified cells were then stained with CD34-PERCPCy5.5, CD45-V500 and CD19-PECy7 labeled antibodies to evaluate the purity of isolated cells. Data acquisition was performed in a FACSCanto II flow cytometer-Becton/Dickinson Biosciences (BDB, San Jose, CA) using the FACSDiva software program and the Infinicyt (Cytognos SL, Salamanca, Spain) software program was used for data analysis. The purity of the isolated tumoral cells was at least 90 %. Purification of CD34+ cells was performed also in normal BM.

### RNA isolation

Total RNA from purified leukemic cells was isolated using the RNeasy Mini Kit (Qiagen) according to the manufacturer’s protocols. RNA was quantified by NanoDrop ND1000 Spectrophotometer (Thermo Scientific, Wilmington, USA) at 260 nm wavelength and the quality was checked using the 2100 Bioanalyzer (Agilent Technologies). Twenty-seven RNA samples Patient’s with RIN ≥7 were used for microarrays experiments.

### RNA labeling for microarrays

Three hundred Nanograms (300 ng) of total RNA were used as the input to produce biotin-labeled cRNA for expression analysis using the TargetAmpNano Labeling Kit for Illumina Expression BeadChip (Epicentre). According to the protocol supplied by the manufacturer, to synthesize biotinylated cRNA, total RNA was reverse-transcribed to cDNA using a T7 oligo(dT) primer. A second-strand of cDNA was synthesized, transcribed in vitro, and labeled with biotin-NTP. After purification with RNeasy Mini Kit (Qiagen) columns, the cRNA was quantified using NanoDrop.

### Microarrays for gene expression profiling

Microarray analysis was performed and analyzed at the Stanley S. Scott Cancer Center’s Translational Genomics Core at LSUHSC. Seven hundred fifty Nanograms (750 ng) of labeled cRNA were hybridized at 58 °C for 16 h to the HumanHT-12 v4 Expression BeadChip (Illumina) which contains more than 47.000 probes interrogating more than 34.000 transcripts following the manufacturer’s instructions (Illumina, Inc., San Diego, USA). After washing and staining with streptavadin-Cy3, the chip was scanned with the BeadArray Reader (Illumina Inc.) The arrays were analyzed using the Illumina’s GenomeStudio software as has been described before by Kim SH [34] and Dai L [35]. Briefly, the signals were normalized using the cubic spline algorithm [36] and the background signal was removed using the Detection *P*-value algorithm that uses the signal of irrelevant probes (with no target in the human transcriptome) but thermodinamically similar to the probes targeting human targets. Differential expression was determined by comparing the condition group (no response to induction therapy) and the reference group (response to induction therapy) using the Illumina Custom algorithm. The microarray experiments were performed in triplicates for each sample and average values were used for analysis. Accession number of microarray data deposited in Gene Expression Omnibus (GEO) is GSE76349.

### Real time-PCR validation

cDNA was synthesized from total RNA using SuperScript III First-Strand Synthesis SuperMix Kit (Invitrogen) according to the manufacturer’s procedures. TaqMan probes were used to quantify the levels of mRNA expression of candidate genes obtained by microarray analysis (Assay IDs: ID3 Hs00954037_g1; ID1 Hs03676575_s1; CMTM8 Hs00418243_m1; IGJ Hs00950678_g1; RGS1 Hs01023772_m1; RPS4Y1 Hs00606158_m1; AGAP1 Hs00963817_m1). The reaction was amplified in a QuantStudio 12 K plex Real-Time PCR machine (Applied Biosystems). The 2^-ΔΔCT^ method was used to estimate the fold induction of each gene using GAPDH and an internal calibrator as controls. Water was used as a negative control. Assays were done in triplicate.

### Statistical analysis

GraphPad graphic software was used to compare the expression levels of the genes between the two groups using Mann Whitney test. Clustering analyses and heatmaps were performed using R-project (www.r-project.org), GenomeStudio (Illumina) and Gene set Enrichment Analysis (GSEA, http://software.broadinstitute.org/gsea/index.jsp). Signaling pathway analysis was done in Metacore and Metacore KPA. Survival analyses were estimated by Kaplan-Meier curves and differences between the survival functions were assessed with the log-rank test. Statistical analysis was performed using SPSS software (version 22 for windows) and R. *p-*values that were <0.05 were considered statistically significant. EFS was calculated from the date of trial enrollment to the date of first event (induction failure, relapse or death). OS was measured from the date of study entry until the date of death.

## Results

### Patient characteristics

This study included 43 adult patients newly diagnosed with Common and Pre-B B-precursor acute lymphoblastic leukemia (B-ALL). Additional file 1: Table S1 describes the clinical and molecular characteristics of the patients. Chromosomal alterations were found only in 5 patients. The number of male and female patients was similar; the median age was 30 years and the median tumoral infiltration at diagnosis determined by flow cytometry was 82 %. The overall outcome of the patients in this cohort was very poor with a median OS of 11 months and 2-years EFS of 58 %. We defined 24 months as a period of follow-up from date of patient’s inclusion to the study.

### Unsupervised hierarchical clustering

**Fig. 1:**
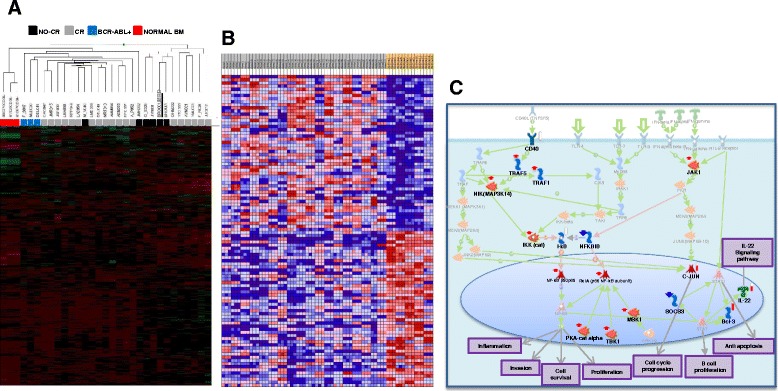
Gene expression profiles of 27 B-ALL bone marrow diagnostic samples. **a** Unsupervised hierarchical clustering is able to differentiate between B-ALL patients. Hierarchical clustering analysis in bone marrow diagnostic samples from 27 adult B-ALL patients revealed 3 main groups included the normal bone marrow grouped together and separately from patient samples. Each square represents 1 sample, each row represents 1 gene. Above, cluster dendrogram of the bone marrow samples. Red square, Normal BM; black square, patients who did not achieve complete remission; blue square, patients with BCR-ABL translocation; grey square, patients who achieve complete remission. **b** Expression analysis of good versus poor induction treatment response patients. Analysis of gene expression from 5 patients who did not respond to induction therapy (yellow) and 22 patients who achieved complete remission (grey and blue). The hierarchical clustering identified 442 genes differentially expressed between both groups with *p* < 0.05. Gene Set Enrichment analysis was used to construct the heatmap showing the top 50 differentially expressed genes. Samples are shown in columns and gene sets are in rows. Increasing (red) or decreasing (blue) gene expression is shown relative to the median (black) for each gene. **c** Signaling pathway analysis using MetaCore revealed activation of different key hubs with *p* < 0.005 in patients with poor response to induction therapy. The total 442 differentially expressed genes were used for pathway analysis. The pathway with the highest activity and involving more of the input genes is the NF-kB signaling. Other signaling pathways like CD40L, IL-9, JAK1, IL-22 appears to be activated in this group of patients. Strong color represents activation key hub (red arrow) or inhibited key hub (blue arrow)

Microarrays experiments were made in 27 of the 43 patient samples. Gene expression data was first analyzed by unsupervised hierarchical clustering. As shown in Fig. 1a, the normal bone marrow samples (red) were clustered together and separated from samples from patients diagnosed with B-ALL. Out of the 27 samples included in the microarrays 3 were positive for *BCR-ABL*–rearrangement and clustered together (blue). In addition, 4 of 5 patients who failed to achieve CR after induction treatment (black) grouped together. It is noteworthy that BM sample of patient BRO001 who did not achieve CR (with a blast percentage of 44 % at the end of induction treatment), grouped together with BRO001_MRD (underlined sample) which corresponds to tumoral residual sample after induction treatment.

### Identification of gene expression profile associated with response to therapy

Supervised analysis was used to identify genes that distinguish non-responders (*n* = 5, black squares in Fig. 1a) from responders (*n* = 22, grey and blue squares in Fig. 1a) to induction treatment. Our analysis identified 442 genes differentially expressed between the two groups (Fig. 1b shows the first 50 differentially expressed genes). After applying additional filters (*p* < 0.05 and fold change > 2) we selected the top 99 genes that distinguished non-responder from responder patients. From this group of genes, 31 were overexpressed in non-responder patients and 68 were over expressed in responder patients to induction treatment. In the non-response group there was a predominant overexpression of genes involved in self-renewal, differentiation, neoplastic transformation (*ID3, ID1*), B cell development (*IGJ*), migration and metastasis (*PLAU*), B cell activation (*CD83*) and oncogenesis (*GFI1*). This pattern suggests a more impaired differentiation and more aggressive behavior in B-ALL cases that did not respond to therapy. The complete set of differentially expressed genes is listed in Additional file 2: Table S2.

We conducted a signaling pathway analysis in Metacore and Metacore KPA using the 442 differentially expressed genes from the two groups of patients. As shown in Fig. 1c, we identified molecular pathways that are aberrantly regulated in the non-response versus the good response group. Among 442 genes, those involved in Nuclear Factor kappa B (NF-kB) signaling are specifically activated in the group of patients who failed to induction chemotherapy treatment. The main alterations in the poor response patients include activation of the kinase complex IKK that phosphorylates I-kB proteins inducing their degradation and NF-kB p50/p65 translocation to the nucleus where it up regulates expression of numerous genes involved in cell survival, proliferation, apoptosis, inflammation, immune response and invasion. Interestingly, in the poor response group both NF-kB subunits p50 and p65 are activated. It is noteworthy that molecules that promote NF-kB signaling, including PKA-cat alpha, TBK1 and MSK1, are also activated. Other top dysregulated pathways in non-responders are involved in lymphocytes proliferation as JAK1 and CD40L signaling and others are involved in anti-apoptosis as pathway generated by IL-9. We also found high activity of c-jun oncogene in this group of patients. All dysregulated pathways derived from non- responders group, are shown in Additional file 3: Table S3.

We also identified molecular interactions and dysregulated pathways in the responders group that could be contributing to successful treatment in these patients. As shown in Additional file 4: Table S4, p53-dependent apoptosis is among the 4 dysregulated key pathway in the remission group. The expression of the *TP53* gene is 16 % increased in this group as compared to the no remission group (data not shown). The pathways dysregulated in the 2 groups (Additional files 5 and 6), are strongly implicated in regulation of leukemic cell functions and several pathways are well known for their role in tumoral development, progression and treatment resistance of different types of leukemia [37–42]. Thus, global pathway analysis allowed us to identify critical biological networks altered in chemotherapy resistant patients.

### Stratification of risk according to gene expression patterns

**Fig. 2:**
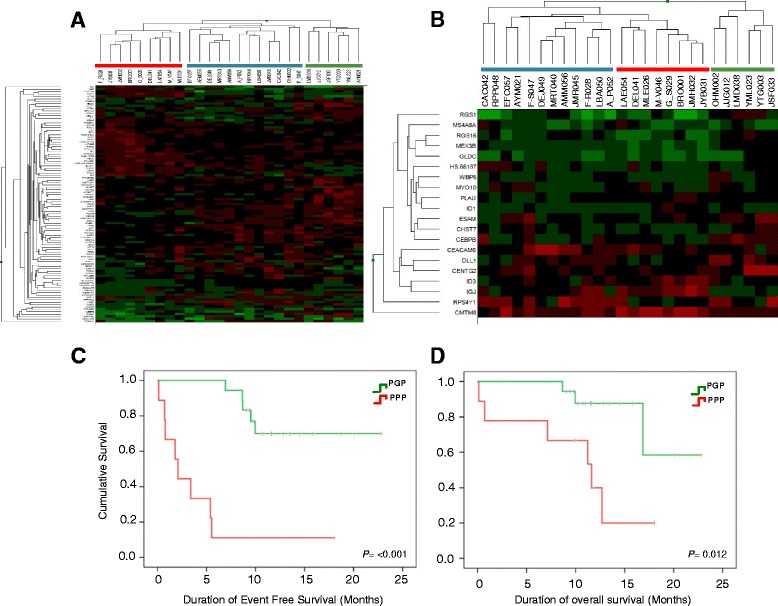
Hierarchical clustering and survival curves of the 27 B-ALL patients based on expression of top selected genes in responding vs. no responding analysis. **a** Top 99 genes providing the biggest expression differences between good and poor response. *p* < 0.05 and fold change >2 were used to cluster the 27 B-ALL patients. **b** Distribution of 20 selected genes in B-ALL from list of 99 genes more differentially expressed between responders and no-responders to induction treatment. Genes with *p* < 0.03, fold change >3 were selected to cluster the 27 B-ALL bone marrow samples. Clustering analysis shows that set of 20 genes can distinguishes the same 3 groups identified with our list of 99 genes candidate predictors of response to therapy (blue, red and green bars). Kaplan Meier curves for EFS (**c**) and OS (**d**) in good and poor prognostic groups according to gene expression profile. Twenty-seven patients were assigned to either predicted good prognosis group (PGP) or predicted poor prognosis group (PPP) based on expression of 99 differentially expressed genes between responders and non-responders to induction therapy

Using unsupervised hierarchical cluster analysis of the top 99 discriminating genes, the samples were separated into three major groups (Fig. 2a). Comparing the clinical characteristics of these groups we found statistically significant differences for age (*p* = 0.049), White Blood Cell Count (WBCC) (*p* = 0.025) and tumoral load in PB at diagnosis (*p* = 0.008). We also found a different trend in hemoglobin, platelets, and tumoral load at diagnostic between groups 1 and 3 (Table 1) located both extremes of heatmap. Group 3 (green bar) included patients who achieved complete remission (6/6), whereas group 1 (red bar) included 5/9 patients with failure to induction therapy. We increased the stringency of the analysis (*p* < 0.03, fold change >3) and found 20 genes that were able to identify the previous same groups in an unsupervised analysis (Fig. 2b). Taken together, our results suggest that gene patterns can be correlated with biological features and can distinguish good and bad prognostic groups in our population.

**Table 1:** Association of expression profiles with high impact prognosis variables

Variable	Group 1	Group 2	Group 3	*P*-value
	(*n*=6)	(*n*=12)	(*n*=9)	(Group 1 vs Group 3)
Age - years				
Median	30	29	21	0,049*
Range	19–63	19–50	16–30	
White blood cell count/ul				
Median	45800	10075	6105	0,025*
Range	1940–412900	1410–170100	1.490–35.970	
Hemoglobin (g/dl)				
Median	7.6	8.85	9.8	0,114
Range	4.15–13.7	5.1–11.5	6.4–13.5	
Platelet count/ul				
Median	27900	15500	140550	0,27
Range	6900–682000	6000–84000	6000–474000	
Bone Marrow blast count in myelogram- (%)				
Median	95	93	84	0,45
Range	61–97	80–97	74–98	
Bone Marrow blast count in Flow cytometry- (%)				
Median	91	90	80	0,28
Range	36–95	54–95	40–95	
Peripheral blood blast count/uL				
Median	41910	173.45	0	0,008*
Range	0–210600	0–124.000	0–2.500	
Complete remision- no. Patients				
Achieve	4/9	12/12	6/6	
Non achieve	5/9	0/12	0/6	

* indicates statistical difference

### Evaluation of the clinical impact of gene expression profile associated with prognosis

To evaluate the clinical impact of our gene expression analysis, we examined the survival of patients in the two risk groups defined by their gene expression profile (group 1 and 3). As can be seen in Fig. 2c and d, there is a statistically significant difference in EFS (log-rank test *p* = 0,001) and OS (log-rank test *p* = 0,012) between the 2 groups. Considering that groups 1 and 3 established by gene expression profile are different in terms of other variables that can strongly predict the prognosis of disease including WBCC (group 1 > 30 000/uL and group 3 < 30 000/uL), age at diagnosis (group 1 > 30 and group 3 < 30 years old) and also in EFS and OS, we defined these two groups as predicted good and poor prognosis groups (PGP and PPP, respectively).

### Confirmation analysis by Real-time quantitative PCR

**Fig. 3:**
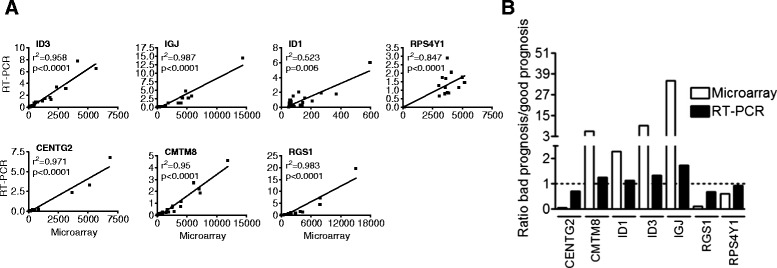
Correlations of expression data between microarrays and RT-PCR. **a** Spearman’s correlation plots show a positive correlation between data obtained by microarrays and RT-PCR. **b** The expression obtained by RT-PCR is consistent with microarrays data for all 7 evaluated genes. CMTM8, ID1, ID3 and IGJ shows an increased expression in bad prognosis group, whereas CENTG2, RGS1 and RPS4Y1 have low expression in this prognostic group in both techniques

To confirm the results of our microarray analysis, we selected the 7 most differentially expressed genes (*CMTM8, ID3, IGJ, RGS1, RPS4Y1, CENTG2, ID1*) between the responder versus non-responder groups and analyzed their expression by semi-quantitative RT-PCR in 43 samples (27 initially included in the microarray analysis plus 16 new samples). As shown in Fig. 3a, there was a significant correlation between the microarray data and RT-PCR for the expression of the 7 validated genes. Figure 3b shows the expression of our 7 genes profile determined by microarrays and RT-PCR in our good and poor prognosis groups. As can be seen, *CMTM8*, *ID1*, *ID3* and *IGJ* are highly expressed in the bad prognosis group, whereas *CENTG2*, *RGS1* and *RPS4Y1* have lower expression in this group of patients and these results are consistent both by microarrays and PCR assays. Taken together, these results support the validity of the expression data obtained by microarrays for this set of 7 genes.

### Validation of gene expression profile for predicting outcome

**Fig. 4:**
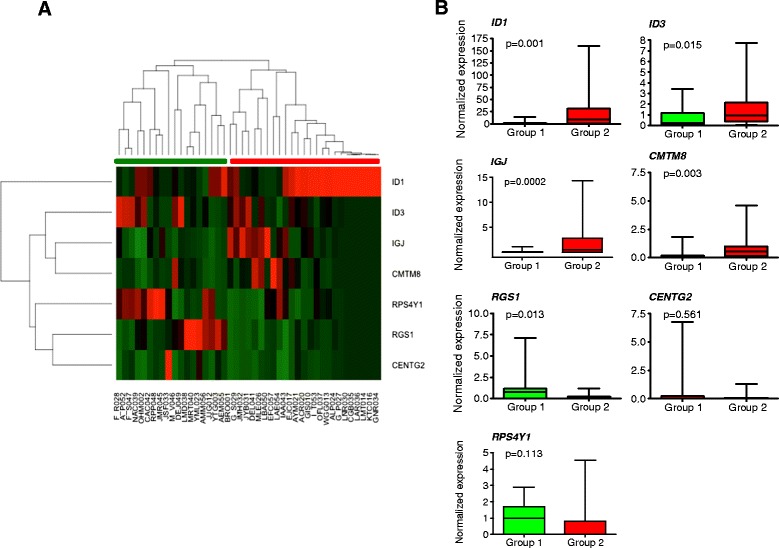
Validation of gene expression profile for outcome prediction. **a** Unsupervised hierarchical clustering analysis applied to 43 patients according to the expression of our 7 genes signature for prognosis prediction. Expression of selected 7 prognostic relevant genes determined by RT-PCR was used to cluster all 43 patients included in the study. Unsupervised cluster distinguished 2 groups of samples (red, bad prognosis; and green, good prognosis). **b** Expression of 7 genes for prognosis prediction in the 2 clustered groups (red and green bars in Figs. 1a and 4a) determined by RT-PCR. Results were normalized against the expression level of GAPDH. High expression of ID3, IGJ, ID1 and CMTM8 was shown to be associated with predicted poor prognosis (PPP)

**Fig. 5:**
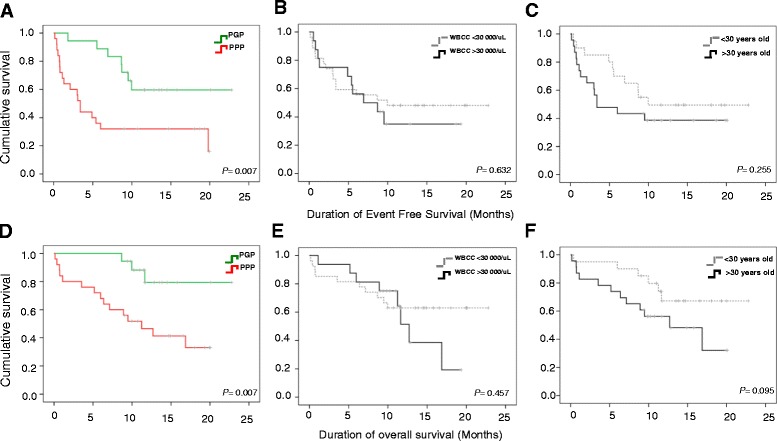
Kaplan Meier survival curves for good and poor prognostic groups according to gene expression profile, WBCC and age. Forty-three patients were assigned to either predicted good prognosis group (PGP) or poor prognosis group (PPP) based on expression of 7 differentially expressed genes. EFS (**a**) and OS (**d**) in predicted groups. Impact of WBCC count at diagnosis on EFS (**b**) and OS (**e**). Impact of age at diagnosis on EFS (**c**) and OS (**f**)

Figure 4a shows the heatmap and dendrogram for the unsupervised cluster analysis applied to 43 patients according to the expression of our 7 genes signature. Interestingly, we found 2 different groups with different gene signatures. The therapy response rate was significantly higher in group 1 (green) than in groups 2 (red) (94 % vs. 60 % respectively) and each group included the same patients classified before as PPP and PGP (respectively) according to microarray analysis (Figs. 2a and 4a). As can be seen in Fig. 4b, for most of the genes (ID1, ID3, IGJ, CMTM8, RGS1) there are statistical significant differences (*p* < 0.05) in their expression between the 2 clustered groups. Both EFS and OS of patients with the predicted poor prognosis signature were significantly shorter than those of the subgroup of patients with a predicted good prognosis signature. Fig. 5a and d show the Kaplan-Meier curves for the predicted prognosis groups based on RT-PCR data (*P =* 0,007 for EFS and 0,007 for OS). As can be seen, the OS predictive power of our gene signature is better with 7 genes than with 99 (Figs. 2d and 5d). As clinical variables such as high WBCC and age at disease onset have been reported to be strongly associated with outcome in B-ALL [19], we also evaluated these conventional variables for predicting outcome in our patients in order to compare their predictive power with our gene expression profile variable. As shown in Fig. 5b, and d for WBCC and 5C and 5 F for age, although older patients and those with high WBCC have worse EFS and OS, the difference was not statistically significant and both conventional parameters appear to be less predictive than our gene expression signature.

Given the consistency of the results obtained by the two methods, we propose that our 7 genes signature could be a potential prognostic factor for the better stratification in risk groups of adult Colombian patients with B-ALL.

### Prognostic impact of ID1/ID3/IGJ expression signature in the context of other clinical and molecular parameters

**Fig. 6:**
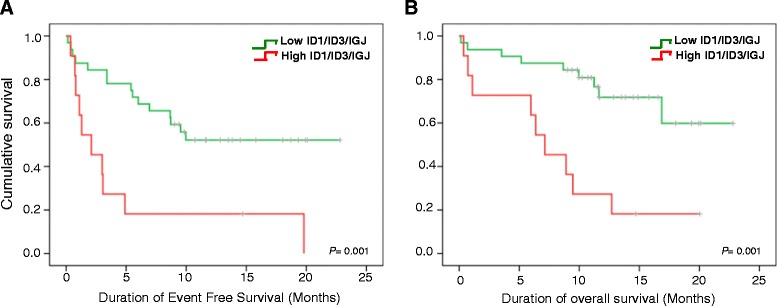
Kaplan Meier curves according to the presence of high risk expression profile. Event free survival (**a**) and Overall Survival (**b**) of patients with simultaneous low ID1/ID3/IGJ expression (green line) vs. patients with simultaneous high ID1/ID3/IGJ expression (red line)

We performed correlation analysis in order to determine which of our 7 genes were the most influential in the prognosis and outcome of patients. Significant correlations were found only between the overexpression of ID3, IGJ and ID1 with the presence of event (*p* = 0.001, 0.015 and 0.017 respectively), and non-CR achievement (*p* = 0.002, 0.010, 0.019 respectively). ID3 and IGJ overexpression were significantly correlated with poor EFS (*p* = 0.003, 0.001 respectively). Overexpression of IGJ tended to correlate with worst OS (*p* = 0.057). According to the potential prognostic value of ID1, ID3 and IGJ, we used a multivariate model analysis with our gene profile in addition to other factors significantly associated with prognosis in univariate analysis for this cohort (clinical and molecular variables with *p* < 0.1 in univariate analysis were entered into multivariate model) to evaluate if simultaneous overexpression of ID1, ID3 and IGJ is an independent prognostic parameter for event risk, CR achievement, EFS and OS. Our gene profile was an independent prognostic parameter for CR achievement, presence of event, EFS and OS in a multivariate model including only clinical variables obtained previous to induction treatment. We also found that our gene profile is an independent prognostic parameter for CR achievement, presence of event and low EFS when we include in our multivariate model the MRD detection variable, which is the most important predictor currently used (Table 2). As can be seen in Fig. 6a and b, patients with high expression of only ID1, ID3 and IGJ genes at time of diagnosis, showed significantly shorter EFS and OS (*p* = 0.001 for EFS and 0.001 for OS). Interestingly, this 3-gene prediction of disease outcome is even better tan previoulsy observed with 7 genes (Fig. 5a and d).

**Table 2:** Prognostic impact of ID1/ID3/IGJ expression signature in the context of other clinical and molecular parameters

	Univariate model		Multivariate model pre-treatment variables		Multivariate model pre-treatment variables and MRD	
Parameter	*p* Value	OR (95 % CI)	*p* Value	OR (95 % CI)	*p* Value	OR (95 % CI)
EVENT						
Gene profile	0.029	6.57 (1.217–35.529)	0.029	6.57 (1.217–35.529)	0.029	6.57 (1.217–35.529)
Age >30	0.052	3.48 (0.990–12.242)	0.120	2.86 (0.759–10.779)	0.405	1.89 (0.420–8.553)
WBCC >30.000/ul	0.090	4.09 (0.803–20.870)	0.315	2.10 (0.494–8.932)	0.152	3.55 (0.628–20.118)
MRD	0.077	4.05 (0.859–19.085)			0.114	3.29 (0.752–14.452)
COMPLETE REMISSION						
Gene profile	0.016	6.48 (1.413–29.713)	0.016	6.48 (1.413–29.713)		
Sex	0.092	0.14 (0.016–1.366)	0.313	0.43 (0.089–2.170)		
EVENT FREE SURVIVAL						
Gene profile	0.004	3.58 (1.493–8.597)	0.004	3.58 (1.493–8.597)	0.017	3.08 (1.223–7.759)
Age >30	0.056	2.40 (0.979–5.922)	0.139	2.00 (0.799–5.027)	0.533	1.37 (0.504–3.760)
MRD	<0.001	1.00 (1.001–1.004)			<0.001	1.002 (1.001–1.003)
OVERALL SURVIVAL						
Gene profile	0.008	3.97 (1.439–111.000)	0.029	3.21 (1.127–9.176)	0.122	2.41 (0.789–7.408)
Platelets count	0.002	1.00 (1.000–1.000)	0.015	1.00 (1.000–1.000)	0.002	1.00 (1.000–1.000)
Age >30	0.060	4.08 (0.944–17.655)	0.025	4.32 (1.204–15.550)	0.060	4.08 (0.944–17.655)
t(9;22)	0.076	0.08 (0.005–1.298)	0.327	0.34 (0.040–2.927)	0.076	0.08 (0.005–1.298)
MRD	<0.001	1.00 (1.002–1.005)			<0.001	1.00 (1.002–1.008)

## Discussion

The main objective of this study was to understand the biology and behavior of B-ALL specifically in a Colombian sample and set the bases for future research of this kind in Hispanic population in order to identify a gene expression signature that may have prognostic value. Given that one of the challenges in the treatment of ALL is to facilitate the development of more appropriate therapeutic approach, the development of new genetic prognostic factors are needed to increase the accuracy of risk stratification. An improved and more accurate patient classification in risk groups with similar clinical outcome would help to devise new strategies for the identification of patients at higher risk to improve their benefits to allogeneic transplant; in addition, those patients with a more favorable prognosis and with less risk of relapse would be helped by receiving less intensive treatment reducing the toxicity and death associated with therapy.

We have identified a set of 442 differentially expressed genes able to distinguish patient subsets with different response to chemotherapy induction treatment. Within the set of genes, we found high expression levels of genes *ID1, ID3, IGJ, CMTM8* in the group of patients with the worst outcome. Interestingly, some of these genes have been associated with leukemia and other tumors. For example, ID family genes such as *ID1* and *ID3* have found to be deregulated in many types of human tumors and contribute to processes such as tumorigenesis, tumor progression, angiogenesis, cell migration, epithelial mesenchymal transition and stem cell renewal [43–55]. The gene *IGJ,* which we found also overexpressed in the poor response group has also been reported as part of a bad prognosis signature in pediatric patients with B-LLA with the worst 4 years-event free survival, high frequency of positive MRD and high Latin Hispanic ethnicity [32]. The gene *CMTM8* is reported as a candidate tumor suppressor gene in an osteosarcoma tumor model suppressing the activity of the oncogenic proteins c-Met and GPR177 [56], however, its role in hematological tumors has not been reported.

Other genes showing altered expression in the poor response group included *CENTG2* (*AGAP1*), *RPS4Y1* and *RGS1*. Interestingly, *CENTG2* has been reported to have dual roles in susceptibility to disease due to its association with a bad prognostic signature in B-ALL in children [32], but having a protective role in gastric cancer [57]. On the other hand, *RGS1* that consistent with our data, was observed under expressed in the high gene expression risk group with the poorest outcome described by Kang et al in a children B-ALL population [58]. In addition, RGS1 encodes a member of the regulator of G-protein signaling family. This protein is strongly expressed in immune cells including germinal center B-lymphocytes, T lymphocytes, natural killer cells, dendritic cells and monocytes. At functional level, RGS1 regulates T-cell migration and B cell homing to lymph nodes in response to chemokines signaling (CXCL12 and CCL19) [59]. Additionally, while the function of *RPS4Y1* is not well described and has no prior link to leukemia, this gene could have a possible role in leukemogenesis based on known functions identified in other models of cancer stem cells resistant to radiation which presents more than 5-fold reduction in the expression of RPS4Y1 [60].

Finally, our set of 3 genes (ID1, ID3 and IGJ) was able to characterize all patients in risk groups, whereas other molecular and clinical prognostic variables including age, WBCC and BCR-ABL translocation showed no clinical impact. Importantly, our results support the fact that it could be possible to use gene expression profiling to predict the outcome of all patients at the time of diagnostic regardless of whether or not they have chromosomal rearrangements. In addition, although the detection of MRD is currently the variable that can predict with greater certainty the prognosis, this determination is made after induction treatment and there are still a high number of patients who die by the high toxicity of induction therapy because of the lack of correct risk stratification. Considering this, it is important to highlight that our 3-gene signature is the independent prognostic parameter with the highest impact between all pre-treatment variables included in our multivariate model. Interestingly, the ID genes, which are significantly implicated in the outcome of our patients, have been reported in other models of cancer as promoters of cell survival and have been associated with the up-regulation of anti-apoptotic pro-survival factors through activation of NF-kB signaling pathway [43], which represents the signaling pathway most aberrantly regulated in the non-response group (Fig. 1c). In addition, some studies have reported that transfection of head and neck squamous cell carcinoma (HNSCC) cells with ID1 in vitro induced the phosphorylation of Akt (p-Akt) via phosphoinositide kinase-3 (PI3K) and increased the expression of survivin via NF-κB [61]. This evidence represents a possible mechanism that might explain the chemo-resistance and poor survival of our patients and also represents a potential opportunity to evaluate the effect of the alteration of our 3-gene signature in the NF-κB signaling pathway. Altogether, these data suggest that simultaneous high expression of ID1, ID3 and IGJ could be associated with poor prognosis in Hispanic adult B-ALL due to its potential to increase tumor cell survival possibly through NF-kB signaling.

Gene expression profiles associated with prognosis have been reported in B-ALL [26, 32, 62]. However most studies are focused on this population since the incidence of this disease is higher in this age group. The fact that there are great differences in genetic and molecular characteristics between adults and children with B-ALL may explain the existence of large differences regarding prognosis. This highlights the need to increase the effort on research directed towards the identification of molecular markers underlying the different outcome of B-ALL in adult patients.

We have to admit the existence if certain limitations in our results. In this study we used a small number of patients due to several difficulties such as the low incidence of this disease in Colombian adult population and also due to both, the multiple barriers that patients have to access health care and the lack of cancer centers that are needed for the establishment and accurate diagnosis and a prompt treatment. Therefore, the number of patients with B-ALL confirmed diagnosis is small and perhaps for this reason there are no studies about the molecular biology of the disease in Hispanic patients. Our prospective study was conducted including all patients who visited the hematology service for 2 years at the National Cancer Institute of Colombia, which is the biggest cancer reference center in our country and also in the hospital Universitario San Ignacio, one of the largest cancer centers in the Country. Even though because of the small number of patients included in our study, our results should be interpreted with caution and await validation in an independent larger cohort. In addition, due to the large genetic variation in Hispanic populations we cannot extrapolate our results to other Latin American Countries. Similarly, comparison and validation of our results in different ethnic groups, including African Americans and Caucasians with B-ALL would support the role of these genes in the pathogenesis of the disease. The study of the response to induction therapy and clinical outcome in different ethnic groups is warranted by the differential response to the treatment associated with ethnicity [9–14]. Even though it is not possible to ensure that Hispanic patients, specifically Colombians, have a worse response and poorer outcome due to biological characteristics inherent to our population, is interesting that even after following the same scheme of treatment, the survival varies greatly by population. So far, ethnic differences in survival of childhood ALL have been reported in many studies, with poorer survival observed among African Americans or those with Hispanic ethnicity when compared with European, Americans or Asians [63–69]. However, up to this moment, the role of ethnicity to the therapy in adults with B-ALL has not been studied. Therefore, it would be interesting to explore whether these differences are due to differences in biological characteristics of patients or due to differences in the chemotherapeutic schemes.

Despite these limitations of our study, we believe it is important that developing countries begin to assess the biology of the disease in their patients. We have described for the first time the transcriptional basis of adult B-ALL in our population, which reflects molecular differences between B-ALL cases with good and poor response to induction chemotherapy. In addition, our comprehensive analysis suggests the basis for future functional analysis that may lead to new potential targets for the treatment of B-ALL, including *ID* gene family, which has been associated with chemo resistance in different models of cancer.

## Conclusions

Overall, our data suggest that high expression of ID1, ID3 and IGJ genes is associated with failure to induction treatment and poor prognosis in adult B-ALL. These analyses identified a subset of high-risk B-ALL adult patients who may benefit from new emerging target therapies or personalized chemotherapy approaches. In addition, assessment of the expression levels of this relatively small number of genes could be easily translated into a clinically useful and inexpensive assay, which could be quite important in developing countries with precarious health systems.

### Ethics approval and consent to participate

This study complied wit all applicable requirements of the ethics committee of the participant institutions. The Institutional Review Board and Ethics Committee of Colombian National Cancer Institute approved this study on August 21 2012 (INT-OFI-007445-2012); Ethics and investigation Committee of Hospital Universitario San Ignacio approved the study on May 13 2014 (FM-CIE-8081-14) signed by Dr. Surella Acosta and Dr. Mary Bermudez, Ethics Committee presidents of Colombian National Cancer Institute and Hospital Universitario San Ignacio respectively. The Institutional Review Boards and Ethics Committee of both institutions approved the Informed consent forms.

### Availability of data and materials

The microarray dataset supporting the conclusions of this article, is available in the Gene Expression Omnibus (GEO) with the accession number GSE76349. (http://www.ncbi.nlm.nih.gov/geo/query/acc.cgi?acc=GSE76349).

## Additional files

Additional file 1:
**Table S1.** Clinical and molecular baseline characteristics of patients. Clinical and molecular data obtained at time of diagnostic of the 43 patients included in the cohort. (PPTX 64 kb) Additional file 2:
**Table S2.** Complete set of genes differentially expressed between good (*n* = 22) versus poor (*n* = 5) induction treatment response patients. Differential expression was performed in Illumina’s GenomeStudio software using the Illumina Custom algorithm. The signals were normalized using the cubic spline algorithm and the background signal removed using the Detection *P*-value algorithm. (PDF 70 kb) Additional file 3:
**Table S3.** Complete list of signaling pathways dysregulated in patients who failed to complete remission therapy. Signaling pathway analysis was done using MetaCore KPA using the set of 442 genes differentially expressed between good and poor response group. (XLSX 10 kb) Additional file 4:
**Table S4.** Complete list of signaling pathways dysregulated in patients who achieved complete remission therapy. Signaling pathway analysis was done using MetaCore KPA using the set of 442 genes differentially expressed between good and poor response group. (XLSX 10 kb) Additional file 5: Complete file derived from pathway analysis in patients who failed to remission therapy. Signaling analysis was done using MetaCore KPA. File shows 10 key pathways dysregulated in this group of patients. (PDF 1174 kb) Additional file 6: Complete file derived from pathway analysis in patients who achieved complete remission. Signaling analysis was done using MetaCore KPA. File shows 4 key pathways dysregulated in this group of patients. (PDF 658 kb)

## Abbreviations

B-ALL: B- acute lymphoblastic leukemia
OS: overall survival
CR: complete remission
EFS: event free survival
ALL: acute lymphoblastic leukemia
DFS: disease free survival
MRD: minimal residual disease
BM: bone marrow
PB: peripheral blood
NF-kB: nuclear factor kappa B
WBCC: white blood cell count
PGP: predicted good prognosis
PPP: predicted poor prognosis
