# Supplementary material for: High expression of ID family and IGJ genes signature as predictor of low induction treatment response and worst survival in adult Hispanic patients with B-acute lymphoblastic leukemia
Source: J Exp Clin Cancer Res. 2016 Apr 5;35:64. doi: 10.1186/s13046-016-0333-z (PMC4820984; doi:10.1186/s13046-016-0333-z)
Supplement: Additional file 1: — Table S1. Clinical and molecular baseline characteristics of patients. Clinical and molecular data obtained at time of diagnostic of the 43 patients included in the cohort. (PPTX 64 kb) [file 13046_2016_333_MOESM1_ESM.pptx]

## Slide 1
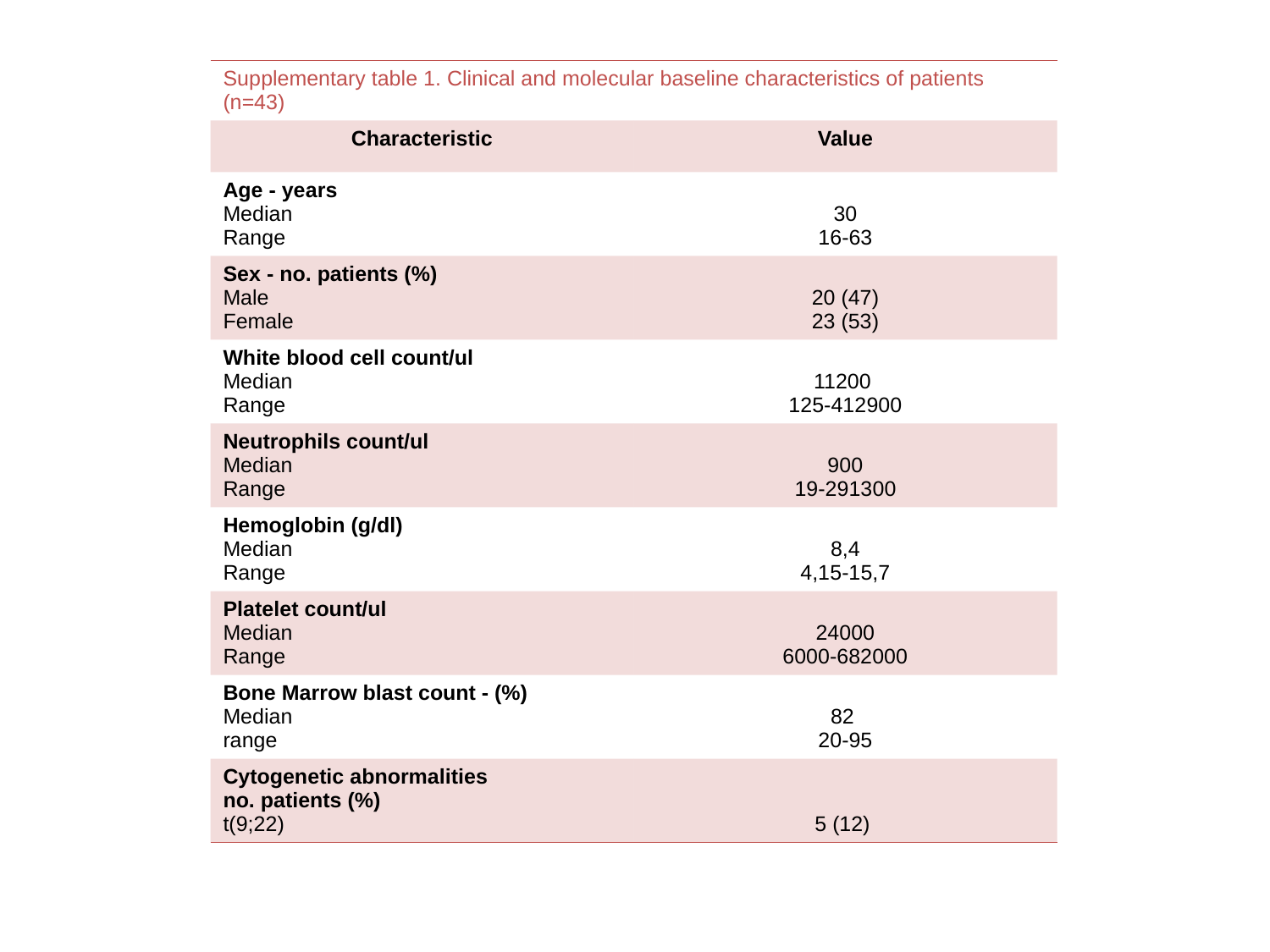

| Supplementary table 1. Clinical and molecular baseline characteristics of patients (n=43) | |
| --- | --- |
| Characteristic | Value |
| Age - years Median Range | 30 16-63 |
| Sex - no. patients (%) Male Female | 20 (47) 23 (53) |
| White blood cell count/ul Median Range | 11200 125-412900 |
| Neutrophils count/ul Median Range | 900 19-291300 |
| Hemoglobin (g/dl) Median Range | 8,4 4,15-15,7 |
| Platelet count/ul Median Range | 24000 6000-682000 |
| Bone Marrow blast count - (%) Median range | 82 20-95 |
| Cytogenetic abnormalities no. patients (%) t(9;22) | 5 (12) |
