# Supplementary material for: High expression of ID family and IGJ genes signature as predictor of low induction treatment response and worst survival in adult Hispanic patients with B-acute lymphoblastic leukemia
Source: J Exp Clin Cancer Res. 2016 Apr 5;35:64. doi: 10.1186/s13046-016-0333-z (PMC4820984; doi:10.1186/s13046-016-0333-z)
Supplement: Additional file 2: — Table S2. Complete set of genes differentially expressed between good (n = 22) versus poor (n = 5) induction treatment response patients. Differential expression was performed in Illumina’s GenomeStudio software using the Illumina Custom algorithm. The signals were normalized using the cubic spline algorithm and the background signal removed using the Detection P-value algorithm. (PDF 70 kb) [file 13046_2016_333_MOESM2_ESM.pdf]

TargetID	Remision	No Remisic	Diff Pval	Ratio Rm/n
RPS4Y1	2112.5	75.9	0.00000	27.84
SLC25A37	207.4	101.7	0.00000	2.04
LOC653778	616.2	282.7	0.00000	2.18
TNS3	681.7	274.1	0.00000	2.49
LOC654103	932.2	413.9	0.00000	2.25
ID3	546.9	3125	0.00000	0.17
TOR1B	263.8	152	0.00000	1.74
LOC729088	101.1	68.9	0.00000	1.47
PCCA	344.4	120.4	0.00000	2.86
MGC18216	228.6	113.2	0.00000	2.02
IQGAP2	607.6	325.5	0.00000	1.87
MEX3B	413.5	132.8	0.00000	3.11
CD83	791.3	2198.4	0.00000	0.36
NELF	670.2	273.9	0.00000	2.45
NKD2	521.7	75.3	0.00001	6.93
ABCA1	1660.9	690.7	0.00001	2.4
RASA2	213.1	128.3	0.00001	1.66
LRRC32	144.3	75	0.00001	1.92
RDH10	113.3	67.9	0.00001	1.67
PRKX	312.3	155.6	0.00003	2.01
SLAIN1	390.8	845.9	0.00003	0.46
RAB11FIP5	90.9	213.3	0.00003	0.43
CCDC24	287.4	644.4	0.00004	0.45
PDE3B	229.2	84.5	0.00004	2.71
HMGB3	162.3	82.2	0.00005	1.97
KCNK3	174.7	78.4	0.00006	2.23
C6ORF192	260.2	140.1	0.00008	1.86
RNASE6	191.5	77.6	0.00009	2.47
CEBPB	1396.8	338.1	0.00011	4.13
TMEM181	537.1	270.3	0.00012	1.99
MYC	1396.2	674.3	0.00012	2.07
PTTG1IP	1948.4	1132.6	0.00013	1.72
CMTM8	1480.9	4601.8	0.00018	0.32
FAM7A1	68.2	109.1	0.00018	0.63
TM9SF3	719.7	425.9	0.00019	1.69
AGPAT5	193.8	113.3	0.00020	1.71
CD99	1017.4	2275.9	0.00020	0.45
KIF20B	373.2	232.8	0.00020	1.6
ID1	80.2	310.4	0.00021	0.26
RASD1	3720.8	1755.8	0.00022	2.12
HS.497591	185.3	98.1	0.00025	1.89
HDAC4	187.5	118.4	0.00028	1.58
HS.412918	162.6	108.3	0.00029	1.5
LRFN3	141.4	68.6	0.00029	2.06
MYO10	266.4	81.4	0.00029	3.27
LHFP	229	135.6	0.00030	1.69

C13ORF27	536.1	225.7	0.00030	2.37
LOC648399	186.1	107.9	0.00031	1.72
RPL23AP53	320.6	163.5	0.00031	1.96
NFKBIE	279	597.4	0.00032	0.47
AURKA	189.7	91.1	0.00034	2.08
CREG1	1027.1	451.5	0.00035	2.27
OAT	400	225.7	0.00036	1.77
SETD6	227.6	126.4	0.00036	1.8
SIPA1L2	872.4	377.3	0.00036	2.31
CCT5	168.1	114.8	0.00036	1.46
LOC644128	354.1	188.2	0.00036	1.88
FAM108C1	993.8	445.2	0.00038	2.23
HS.444291	77.2	132.3	0.00038	0.58
LANCL2	185.5	121.1	0.00038	1.53
WFS1	1591.7	594.4	0.00039	2.68
MOSPD1	138	97.8	0.00040	1.41
ITPRIPL2	518.7	179.4	0.00043	2.89
UCK2	215.6	120	0.00043	1.8
LAMC1	687.9	367.2	0.00045	1.87
ZEB2	1130.6	683.4	0.00047	1.65
SUCLA2	389.1	239.6	0.00051	1.62
LOC730256	95.8	149.2	0.00057	0.64
H1FX	478.5	897.8	0.00065	0.53
C14ORF4	1018.6	1990.3	0.00067	0.51
EHBP1	494.8	268.6	0.00070	1.84
C9ORF21	205.4	95	0.00078	2.16
APLP2	125.9	85.2	0.00080	1.48
AMN1	143.8	102.4	0.00082	1.41
CCNJ	206.3	93	0.00086	2.22
PRIC285	319.2	191.1	0.00086	1.67
JUN	4038.1	7769.9	0.00088	0.52
QSOX2	928.2	505	0.00090	1.84
CTR9	295	169.5	0.00098	1.74
IL17RA	220.6	109.9	0.00099	2.01
RHOBTB3	211.5	93.5	0.00105	2.26
NT5C3	997.6	630.4	0.00116	1.58
FAM43A	374.7	175.6	0.00122	2.13
CALU	200.2	132.4	0.00128	1.51
FAM64A	127.6	79.3	0.00133	1.61
SLC1A3	114.3	70.1	0.00137	1.63
LOC100129668	139.1	225.4	0.00145	0.62
BRWD3	181.6	110.5	0.00149	1.64
NOP16	180.1	126.3	0.00167	1.43
EPDR1	557	233.1	0.00169	2.39
RIPK2	997.8	2064.2	0.00170	0.48
POLR3B	400	262.1	0.00179	1.53
LOC100133760	310.6	200.7	0.00187	1.55

OPN3	148.7	101.3	0.00194	1.47
ORMDL3	195.9	385.2	0.00194	0.51
MGST3	562.6	346.4	0.00198	1.62
TBL1X	262.6	143	0.00198	1.84
WBP5	262.1	75.7	0.00209	3.46
C14ORF181	210.8	391.5	0.00216	0.54
TBCD	265.9	554.3	0.00217	0.48
TARS2	172	122.1	0.00223	1.41
TYROBP	267	109.4	0.00223	2.44
C5	332.2	603.3	0.00226	0.55
HS.409512	145.8	89.5	0.00233	1.63
SEC23B	586.3	381.8	0.00263	1.54
MT1F	325.8	135.3	0.00266	2.41
XIST	987.4	2063.2	0.00273	0.48
IL3RA	295.9	136.7	0.00281	2.17
ESAM	347.7	98.7	0.00284	3.52
PIM2	371	656.3	0.00285	0.57
DKC1	2299.1	1449.8	0.00292	1.59
HSPC111	322.4	217.1	0.00293	1.49
HS.579530	488.1	1150	0.00301	0.42
SNORD56	249.6	151.2	0.00333	1.65
ZCCHC6	220.4	139.7	0.00345	1.58
GIMAP4	1320.5	681.6	0.00345	1.94
PTGER4	1726	4147	0.00347	0.42
KIAA0427	131.1	93.1	0.00372	1.41
EPB41L2	191	113.4	0.00376	1.68
UTRN	143.6	103.5	0.00385	1.39
CHST7	372.9	124.3	0.00389	3
TAF5	243.6	160.9	0.00395	1.51
WDR49	190.2	310.7	0.00405	0.61
MS4A6A	292.5	95.7	0.00411	3.06
MED11	123.2	92.4	0.00414	1.33
NFKBIA	6312.6	11244.7	0.00414	0.56
LOC654260	138.7	234.2	0.00418	0.59
EMP3	361.8	622.9	0.00420	0.58
DHX33	217.7	152.9	0.00422	1.42
RHOB	929.5	2628.7	0.00429	0.35
HS.66187	284.3	93.4	0.00446	3.04
LY6E	449.7	295.4	0.00446	1.52
DEPDC6	87.7	66.6	0.00455	1.32
HBEGF	1899.1	1025.5	0.00460	1.85
CCDC99	292.9	191.2	0.00469	1.53
MT1A	683.7	322	0.00469	2.12
SNORA61	528.7	337.6	0.00483	1.57
B4GALT5	230	106.7	0.00493	2.15
RAB20	109	73.9	0.00502	1.47
TSR1	137.2	100.7	0.00510	1.36

PLAU	126	387.1	0.00514	0.33
LOC727820	307.1	567.1	0.00522	0.54
C10ORF50	95.9	159.3	0.00523	0.6
C5ORF33	232.9	158.3	0.00523	1.47
LOC652771	121.9	85.3	0.00532	1.43
KIAA1279	180	123.8	0.00543	1.45
MTE	122	72.5	0.00543	1.68
TICAM1	250	449.7	0.00558	0.56
IL10RA	129.8	234.7	0.00574	0.55
FKBP1A	433.6	284.2	0.00576	1.53
LOC729680	110.4	76.6	0.00581	1.44
LOC646993	158.4	85.7	0.00603	1.85
PGM2	279.9	184.7	0.00603	1.52
LOC100128899	89.9	67.9	0.00603	1.32
DDN	124.9	81.4	0.00628	1.53
ETS1	1338.5	683.3	0.00640	1.96
PRRC1	551.5	364.9	0.00645	1.51
ARL9	154.4	257.9	0.00659	0.6
LOC100188949	666.9	1669.6	0.00659	0.4
ZHX2	1116.3	711.9	0.00686	1.57
EFHC2	139.2	70.8	0.00712	1.97
TMEM2	580.1	295	0.00714	1.97
ZNF185	395.9	200	0.00726	1.98
G3BP1	355.5	214.1	0.00727	1.66
WARS2	101.3	75.5	0.00728	1.34
KANK2	223.8	123.8	0.00734	1.81
TIFA	187.6	381	0.00734	0.49
HK2	641.4	305	0.00736	2.1
TTC39C	227	361.1	0.00744	0.63
TCF7L2	192.2	112.9	0.00751	1.7
TGFBR3	171.9	95.7	0.00752	1.8
PFAS	884	585.5	0.00758	1.51
BIVM	141	64.7	0.00769	2.18
LOC729708	1307.8	807.8	0.00780	1.62
CDC2	150.8	94.5	0.00781	1.59
C11ORF82	205.3	135.9	0.00785	1.51
BNIP3	638.8	336.9	0.00786	1.9
CHERP	245.3	163.5	0.00791	1.5
NT5E	804.1	1705.5	0.00794	0.47
SDCBP	475.5	324	0.00801	1.47
SEH1L	249.1	173	0.00807	1.44
CBLN3	171.7	308.1	0.00809	0.56
TPBG	195.6	80.9	0.00825	2.42
GCNT1	305.9	159.2	0.00850	1.92
HDGFRP3	264.9	92.6	0.00851	2.86
FAM65B	1113.4	2028.9	0.00852	0.55
MAP2K4	265.8	175.9	0.00853	1.51

DENND3	1053.3	1849.2	0.00871	0.57
IQCG	134.2	199.6	0.00886	0.67
DYNLL2	561.7	1233.2	0.00908	0.46
NEXN	308.8	191.5	0.00926	1.61
TRNT1	177.1	131.9	0.00942	1.34
C18ORF19	230.2	114.1	0.00947	2.02
LEPROTL1	517.8	332.7	0.00967	1.56
GADD45A	1034.4	561.8	0.00968	1.84
MTHFD2L	229.2	146.5	0.01027	1.56
FUCA1	595.9	365.1	0.01030	1.63
TFAP4	166.1	119.6	0.01048	1.39
IGJ	1108.1	5970.9	0.01060	0.19
FAM44A	107.8	77.6	0.01075	1.39
SLC39A14	117.3	79.8	0.01075	1.47
HS.104091	72.7	96.2	0.01084	0.76
MAP3K5	254.4	176.5	0.01088	1.44
SIRT1	1029.3	664.8	0.01105	1.55
MOBKL1A	306.3	186.4	0.01106	1.64
CECR6	177	81.7	0.01124	2.17
ACSL1	844.3	372.6	0.01130	2.27
BSPRY	169.8	266.2	0.01130	0.64
HS.60257	227.2	119.2	0.01130	1.91
KLF11	329.4	797	0.01148	0.41
STAMBPL1	364.4	627.9	0.01148	0.58
FAM101B	112.2	79.9	0.01151	1.4
NPHP4	171.5	102.4	0.01159	1.67
RNMT	560.8	334.3	0.01162	1.68
IER3IP1	532.7	364.1	0.01164	1.46
ALG3	174.6	143.4	0.01173	1.22
HS.532698	297.3	184.3	0.01174	1.61
GFI1	380.5	921.4	0.01175	0.41
ZFPM1	99.4	75.1	0.01210	1.32
KLRF1	111.3	74.5	0.01227	1.49
MIR155HG	482.9	944.9	0.01255	0.51
GTF3A	3670.1	6112.8	0.01287	0.6
RGPD2	108.3	69	0.01317	1.57
TNFSF13B	209.3	144.7	0.01374	1.45
FRAT2	656.9	443.9	0.01417	1.48
PRDX3	870.8	583.1	0.01420	1.49
LOC401623	131.9	100	0.01478	1.32
LOC93622	278.3	192.5	0.01488	1.45
EAF2	678.4	1096.4	0.01524	0.62
GLDC	538.5	141.6	0.01544	3.8
RGPD8	169.2	107.8	0.01567	1.57
RGS18	417.5	74.3	0.01567	5.62
KIF11	306.3	173.6	0.01571	1.76
SV2A	126.2	189.9	0.01582	0.66

IER5	823.1	1357.8	0.01585	0.61
RSPH3	215.3	328.8	0.01585	0.65
GCET2	132.3	218.7	0.01589	0.6
CHST2	179.2	73.8	0.01601	2.43
DLAT	328.3	232.1	0.01601	1.41
SUSD2	82.9	113.8	0.01601	0.73
QDPR	461.8	318.9	0.01617	1.45
LOC90925	1580.5	657.4	0.01621	2.4
DNASE2	217	504.3	0.01629	0.43
GCLM	178.6	152.3	0.01639	1.17
CUGBP1	97.4	77.3	0.01645	1.26
DLL1	1477.4	329.1	0.01650	4.49
LCN2	126.6	74	0.01650	1.71
ZNF792	415.6	261.8	0.01656	1.59
CORO1C	209.3	106	0.01676	1.97
LOC730525	318.3	180.9	0.01678	1.76
OAF	167.7	124.6	0.01678	1.35
EMILIN2	715.1	367.3	0.01722	1.95
RRM2	101.2	74.5	0.01722	1.36
LOC441150	116.5	164.8	0.01730	0.71
ZNF721	623.4	429.8	0.01772	1.45
PGRMC1	531.6	368.7	0.01775	1.44
TSHZ1	302.3	196.7	0.01775	1.54
CLASP2	184.2	134.4	0.01800	1.37
FBXO34	598.4	370.8	0.01809	1.61
C19ORF42	263	189.6	0.01842	1.39
FABP5L2	1115.5	742.4	0.01843	1.5
CD93	256	145.6	0.01868	1.76
CD40	78.2	132.2	0.01879	0.59
NCOR1	141.6	106.8	0.01951	1.33
MRPL19	302.2	216.3	0.01964	1.4
LOC727935	84.9	117.5	0.02028	0.72
FAM100B	585.9	996	0.02029	0.59
RNF141	177.5	131	0.02034	1.35
CBR1	103.3	73.4	0.02034	1.41
CD27	352.2	805.7	0.02034	0.44
TOP2A	628.5	275.1	0.02122	2.28
LRRC58	287.3	206.4	0.02127	1.39
MS4A1	140.2	249.1	0.02133	0.56
ICAM3	921.1	1533.4	0.02144	0.6
HS.493947	212.9	78.9	0.02146	2.7
SPAG5	88.7	68.8	0.02185	1.29
APAF1	578.8	355.9	0.02212	1.63
CEACAM6	1031.7	195.1	0.02279	5.29
ID2	2084.7	4209.5	0.02314	0.5
C5ORF30	204	139	0.02316	1.47
PPM2C	203.4	147.6	0.02336	1.38

LRRC28	232.8	149.2	0.02376	1.56
ITGB7	381.2	757.9	0.02398	0.5
PPAT	330.2	236.5	0.02406	1.4
IRF1	1574.8	2592.3	0.02507	0.61
LOC650919	191.5	348.7	0.02507	0.55
GTF2IP1	623.1	418.3	0.02512	1.49
NGFRAP1	206.5	79.6	0.02535	2.59
DHRS3	949.8	1524	0.02537	0.62
AHI1	288.4	122.3	0.02538	2.36
3-Mar	255.9	177.4	0.02563	1.44
ASF1B	112.4	74	0.02569	1.52
UBXN8	90.2	70.7	0.02569	1.27
RGS16	621.9	192.5	0.02592	3.23
NDRG1	837.1	525.7	0.02607	1.59
SOX8	108.8	68.8	0.02620	1.58
KCNK6	134.7	94.7	0.02620	1.42
PGBD4	92.4	72.9	0.02668	1.27
PIGM	248.9	167.4	0.02671	1.49
PUS7	405.5	282.9	0.02695	1.43
LOC100133077	104.9	72.4	0.02697	1.45
UAP1	318.9	208.4	0.02712	1.53
PLAGL1	111.8	76.7	0.02719	1.46
PRR7	142.4	254.4	0.02725	0.56
SMAD7	460.4	814.3	0.02734	0.57
CALD1	128.7	71.7	0.02738	1.79
COMMD10	331.2	233.9	0.02744	1.42
LOC643985	127.1	85.6	0.02751	1.48
CAV1	133.5	78.5	0.02756	1.7
LOC731314	652.6	429.3	0.02794	1.52
ZSCAN5A	112.8	85.6	0.02794	1.32
C6ORF203	151.1	115.7	0.02799	1.31
BAHCC1	133.2	91.8	0.02823	1.45
GOLGA4	208.9	153.5	0.02857	1.36
PAPD5	534.9	345.6	0.02857	1.55
ZC3H18	349	250.6	0.02857	1.39
ADIPOR2	845	581.3	0.02885	1.45
GTF2H2	128	90.1	0.02901	1.42
FOXH1	89.7	70.3	0.02958	1.28
RYBP	1172.4	641	0.02958	1.83
SPTLC2	156.9	110.9	0.02958	1.42
DAPP1	4247.3	4549.7	0.02964	0.93
BCL3	338.4	538.1	0.02970	0.63
PAK1	315.1	494.4	0.02982	0.64
CSF1R	651.8	307.3	0.02990	2.12
LOC387882	562.3	370.9	0.02997	1.52
MT2A	227.8	129.4	0.03009	1.76
KIAA1430	133.9	104	0.03030	1.29

CENPE	146.7	104.5	0.03034	1.4
INO80C	413.7	295.3	0.03045	1.4
CENTG2	1026.3	120.3	0.03078	8.53
RGL4	151.9	307.5	0.03085	0.49
C14ORF169	287.6	196.1	0.03128	1.47
RUFY3	227.3	404.6	0.03144	0.56
ASPM	476.5	272.6	0.03164	1.75
ASAP2	179.4	276.8	0.03196	0.65
PAM	664.1	351.4	0.03241	1.89
GM2A	91.5	74.4	0.03254	1.23
ICOSLG	135.3	102.6	0.03254	1.32
VPREB3	2251.9	3767.9	0.03254	0.6
C5ORF39	660.3	1174.8	0.03277	0.56
CCDC102A	398.2	252.6	0.03277	1.58
C3ORF21	270.5	194.6	0.03288	1.39
EXT1	109.3	78.6	0.03300	1.39
VRK1	526.8	369.1	0.03314	1.43
ADRA2A	97.7	78.4	0.03320	1.25
CABLES1	228.6	104.1	0.03387	2.2
HMMR	412.5	210.9	0.03418	1.96
LOC642299	270	171.5	0.03459	1.57
VIL2	6882.2	4726.4	0.03543	1.46
FASTKD2	136.3	103.6	0.03550	1.32
GRK5	342.2	178.3	0.03560	1.92
COPG2	197.2	142.4	0.03570	1.38
SPRED2	159.6	80.6	0.03570	1.98
BUB1	229.3	146.3	0.03573	1.57
CENPQ	145.2	97.6	0.03596	1.49
ZCCHC24	130	101.8	0.03635	1.28
MAP2K1	1206.6	834.1	0.03654	1.45
STK17B	869.5	1470.5	0.03658	0.59
RPL39L	285.2	173.1	0.03679	1.65
GPR146	99	77.9	0.03695	1.27
MBLAC2	200.7	136.8	0.03700	1.47
LOC283932	214.4	158.1	0.03722	1.36
LOC642489	899	577.6	0.03733	1.56
KDELR2	493.6	330.5	0.03783	1.49
C1ORF69	145.5	111.9	0.03798	1.3
TRIP6	392.8	596.9	0.03850	0.66
C9ORF140	93.7	70.8	0.03872	1.32
CDCA7L	280.6	444.4	0.03888	0.63
GPD1L	579.1	408.8	0.03888	1.42
PDIA5	250.3	150.3	0.03906	1.67
DDEF2	225.7	350.2	0.03915	0.64
LOC651621	92.2	144.3	0.03915	0.64
PPAN	88.8	71.3	0.03915	1.24
CHN2	185.7	413.9	0.03917	0.45

SNX4	485.5	347.4	0.03918	1.4
UBE2Q1	409.7	293.4	0.03918	1.4
RGS1	2710.4	732.5	0.03996	3.7
LOC201175	124.3	250.5	0.04021	0.5
OAS2	387.6	210.4	0.04030	1.84
HLA-DRB6	1768	3276.3	0.04043	0.54
SLC25A43	227.2	138.1	0.04060	1.64
GLCE	136.7	83.7	0.04060	1.63
FAR2	287.8	210	0.04065	1.37
LMTK2	143.1	92.3	0.04117	1.55
LOC100133609	201.9	152.2	0.04117	1.33
NOV	231.3	97.2	0.04117	2.38
SLC25A4	173.1	98.5	0.04156	1.76
IFNGR1	702.2	491.9	0.04168	1.43
PGRMC2	787.5	544.9	0.04168	1.45
ATP8B4	150.4	100.8	0.04187	1.49
FLI1	1641.7	1136.8	0.04202	1.44
LOC100129960	109.8	85.9	0.04276	1.28
EVI2B	718.5	1288.3	0.04286	0.56
WDR40A	462.6	257	0.04312	1.8
ARHGEF17	160	264.1	0.04331	0.61
STX2	229	151.1	0.04331	1.52
FEZ2	833.7	588.9	0.04346	1.42
RYK	214.6	128.9	0.04346	1.66
C9ORF119	285.1	419.4	0.04378	0.68
TF	151	87.6	0.04402	1.72
SLC12A2	280.3	201.7	0.04433	1.39
CHCHD7	494.1	364	0.04440	1.36
PDCL	153.2	116.2	0.04457	1.32
LRRC56	118.6	161.3	0.04464	0.74
PITPNA	140.5	105.9	0.04478	1.33
LRMP	2285.9	3618.8	0.04479	0.63
LDB2	553.7	193	0.04484	2.87
NCF1	142.2	91.3	0.04484	1.56
MAK16	628.6	448.7	0.04493	1.4
TLR1	516.3	1069.9	0.04493	0.48
LOC387934	445.8	320.5	0.04499	1.39
MTFR1	104.8	77.8	0.04499	1.35
CENPF	196.5	117.2	0.04520	1.68
MARS2	153.4	107.1	0.04524	1.43
LOC100133697	202	152	0.04548	1.33
LOC286367	367.2	559.8	0.04548	0.66
TSPAN33	684.4	1036.4	0.04548	0.66
ELL2	311.4	180.1	0.04575	1.73
TTC7A	102.6	136.4	0.04585	0.75
LGMN	271	595.5	0.04587	0.46
SLC45A3	216.6	410.7	0.04603	0.53

ITGB2	3791.7	2190.4	0.04609	1.73
TRIB1	526.5	355.8	0.04616	1.48
LOC791120	839.9	1369.5	0.04619	0.61
HS.551137	147.5	114.1	0.04625	1.29
SLC6A16	124.1	90.8	0.04626	1.37
SERPINI1	117.3	88.8	0.04678	1.32
NEK2	217.8	135.3	0.04699	1.61
MGC33556	2007.6	3154.8	0.04710	0.64
CACNB3	437.8	652.7	0.04734	0.67
FADD	263.6	191.4	0.04777	1.38
HMGCS1	535.3	802.8	0.04777	0.67
CEP72	140.5	110.1	0.04791	1.28
ANGPT1	160.8	79.3	0.04865	2.03
RARS	952.2	675.3	0.04874	1.41
TEX10	337.1	247.4	0.04879	1.36
CPT2	208.5	148.3	0.04910	1.41
THOC3	186.5	131.7	0.04917	1.42
ZNF318	544.4	315.7	0.04972	1.72
HS2ST1	193.7	146.1	0.04977	1.33
SDK2	502	825.2	0.04990	0.61

DEFINITION
Homo sapiens ribosomal protein S4, Y-linked 1 (RPS4Y1), mRNA.
Homo sapiens solute carrier family 25, member 37 (SLC25A37), nuclear gene encoding mitochondrial protein, mRI
PREDICTED: Homo sapiens similar to solute carrier family 25, member 37 (LOC653778), mRNA.
Homo sapiens tensin 3 (TNS3), mRNA.
PREDICTED: Homo sapiens similar to solute carrier family 25, member 37 (LOC654103), mRNA.
Homo sapiens inhibitor of DNA binding 3, dominant negative helix-loop-helix protein (ID3), mRNA.
Homo sapiens torsin family 1, member B (torsin B) (TOR1B), mRNA.
PREDICTED: Homo sapiens misc_RNA (LOC729088), miscRNA.
Homo sapiens propionyl Coenzyme A carboxylase, alpha polypeptide (PCCA), nuclear gene encoding mitochondria
PREDICTED: Homo sapiens hypothetical protein MGC18216 (MGC18216), mRNA.
Homo sapiens IQ motif containing GTPase activating protein 2 (IQGAP2), mRNA.
Homo sapiens mex-3 homolog B (C. elegans) (MEX3B), mRNA.
Homo sapiens CD83 molecule (CD83), transcript variant 1, mRNA.
Homo sapiens nasal embryonic LHRH factor (NELF), mRNA.
Homo sapiens naked cuticle homolog 2 (Drosophila) (NKD2), mRNA.
Homo sapiens ATP-binding cassette, sub-family A (ABC1), member 1 (ABCA1), mRNA.
Homo sapiens RAS p21 protein activator 2 (RASA2), mRNA.
Homo sapiens leucine rich repeat containing 32 (LRRC32), mRNA.
Homo sapiens retinol dehydrogenase 10 (all-trans) (RDH10), mRNA.
Homo sapiens protein kinase, X-linked (PRKX), mRNA.
Homo sapiens SLAIN motif family, member 1 (SLAIN1), transcript variant 1, mRNA.
Homo sapiens RAB11 family interacting protein 5 (class I) (RAB11FIP5), mRNA.
Homo sapiens coiled-coil domain containing 24 (CCDC24), mRNA.
Homo sapiens phosphodiesterase 3B, cGMP-inhibited (PDE3B), mRNA.
Homo sapiens high-mobility group box 3 (HMGB3), mRNA.
Homo sapiens potassium channel, subfamily K, member 3 (KCNK3), mRNA.
Homo sapiens chromosome 6 open reading frame 192 (C6orf192), mRNA.
Homo sapiens ribonuclease, RNase A family, k6 (RNASE6), mRNA.
Homo sapiens CCAAT/enhancer binding protein (C/EBP), beta (CEBPB), mRNA.
Homo sapiens transmembrane protein 181 (TMEM181), mRNA.
Homo sapiens v-myc myelocytomatosis viral oncogene homolog (avian) (MYC), mRNA.
Homo sapiens pituitary tumor-transforming 1 interacting protein (PTTG1IP), mRNA.
Homo sapiens CKLF-like MARVEL transmembrane domain containing 8 (CMTM8), mRNA.
PREDICTED: Homo sapiens family with sequence similarity 7, member A1, transcript variant 4 (FAM7A1), mRNA.
Homo sapiens transmembrane 9 superfamily member 3 (TM9SF3), mRNA.
Homo sapiens 1-acylglycerol-3-phosphate O-acyltransferase 5 (lysophosphatidic acid acyltransferase, epsilon) (AG
Homo sapiens CD99 molecule (CD99), transcript variant 1, mRNA.
Homo sapiens kinesin family member 20B (KIF20B), mRNA.
Homo sapiens inhibitor of DNA binding 1, dominant negative helix-loop-helix protein (ID1), transcript variant 2, ml
Homo sapiens RAS, dexamethasone-induced 1 (RASD1), mRNA.
Homo sapiens cDNA FLJ41846 fis, clone NT2RI3003162
Homo sapiens histone deacetylase 4 (HDAC4), mRNA.
Homo sapiens cDNA FLJ32550 fis, clone SPLEN1000056
Homo sapiens leucine rich repeat and fibronectin type III domain containing 3 (LRFN3), mRNA.
Homo sapiens myosin X (MYO10), mRNA.
Homo sapiens lipoma HMGIC fusion partner (LHFP), mRNA.

Homo sapiens chromosome 13 open reading frame 27 (C13orf27), mRNA.
PREDICTED: Homo sapiens similar to Ornithine aminotransferase, mitochondrial precursor (Ornithine--oxo-acid an
Homo sapiens ribosomal protein L23a pseudogene 53 (RPL23AP53), non-coding RNA.
Homo sapiens nuclear factor of kappa light polypeptide gene enhancer in B-cells inhibitor, epsilon (NFKBIE), mRN
Homo sapiens aurora kinase A (AURKA), transcript variant 5, mRNA.
Homo sapiens cellular repressor of E1A-stimulated genes 1 (CREG1), mRNA.
Homo sapiens ornithine aminotransferase (gyrate atrophy) (OAT), nuclear gene encoding mitochondrial protein, n
Homo sapiens SET domain containing 6 (SETD6), mRNA.
Homo sapiens signal-induced proliferation-associated 1 like 2 (SIPA1L2), mRNA.
Homo sapiens chaperonin containing TCP1, subunit 5 (epsilon) (CCT5), mRNA.
PREDICTED: Homo sapiens hypothetical protein LOC644128 (LOC644128), mRNA.
Homo sapiens family with sequence similarity 108, member C1 (FAM108C1), mRNA.
full-length cDNA clone CS0DF003YI18 of Fetal brain of Homo sapiens (human)
Homo sapiens LanC lantibiotic synthetase component C-like 2 (bacterial) (LANCL2), mRNA.
Homo sapiens Wolfram syndrome 1 (wolframin) (WFS1), mRNA.
Homo sapiens motile sperm domain containing 1 (MOSPD1), mRNA.
Homo sapiens inositol 1,4,5-triphosphate receptor interacting protein-like 2 (ITPRIPL2), mRNA.
Homo sapiens uridine-cytidine kinase 2 (UCK2), mRNA.
Homo sapiens laminin, gamma 1 (formerly LAMB2) (LAMC1), mRNA.
Homo sapiens zinc finger E-box binding homeobox 2 (ZEB2), mRNA.
Homo sapiens succinate-CoA ligase, ADP-forming, beta subunit (SUCLA2), mRNA.
PREDICTED: Homo sapiens hypothetical protein LOC730256 (LOC730256), mRNA.
Homo sapiens H1 histone family, member X (H1FX), mRNA.
Homo sapiens chromosome 14 open reading frame 4 (C14orf4), mRNA.
Homo sapiens EH domain binding protein 1 (EHBP1), mRNA.
Homo sapiens chromosome 9 open reading frame 21 (C9orf21), mRNA.
Homo sapiens amyloid beta (A4) precursor-like protein 2 (APLP2), mRNA.
Homo sapiens antagonist of mitotic exit network 1 homolog (S. cerevisiae) (AMN1), mRNA.
Homo sapiens cyclin J (CCNJ), mRNA.
Homo sapiens peroxisomal proliferator-activated receptor A interacting complex 285 (PRIC285), transcript variant
Homo sapiens jun oncogene (JUN), mRNA.
Homo sapiens quiescin Q6 sulfhydryl oxidase 2 (QSOX2), mRNA.
Homo sapiens Ctr9, Paf1/RNA polymerase II complex component, homolog (S. cerevisiae) (CTR9), mRNA.
Homo sapiens interleukin 17 receptor A (IL17RA), mRNA.
Homo sapiens Rho-related BTB domain containing 3 (RHOBTB3), mRNA.
Homo sapiens 5'-nucleotidase, cytosolic III (NT5C3), transcript variant 1, mRNA.
Homo sapiens family with sequence similarity 43, member A (FAM43A), mRNA.
Homo sapiens calumenin (CALU), mRNA.
Homo sapiens family with sequence similarity 64, member A (FAM64A), mRNA.
Homo sapiens solute carrier family 1 (glial high affinity glutamate transporter), member 3 (SLC1A3), mRNA.
PREDICTED: Homo sapiens hypothetical protein LOC100129668 (LOC100129668), mRNA.
Homo sapiens bromodomain and WD repeat domain containing 3 (BRWD3), mRNA.
Homo sapiens NOP16 nucleolar protein homolog (yeast) (NOP16), mRNA.
Homo sapiens ependymin related protein 1 (zebrafish) (EPDR1), mRNA.
Homo sapiens receptor-interacting serine-threonine kinase 2 (RIPK2), mRNA.
Homo sapiens polymerase (RNA) III (DNA directed) polypeptide B (POLR3B), mRNA.
PREDICTED: Homo sapiens similar to Jumonji, AT rich interactive domain 1B (RBP2-like) (LOC100133760), partial n

Homo sapiens opsin 3 (encephalopsin, panopsin) (OPN3), transcript variant 2, mRNA.
Homo sapiens ORM1-like 3 (S. cerevisiae) (ORMDL3), mRNA.
Homo sapiens microsomal glutathione S-transferase 3 (MGST3), mRNA.
Homo sapiens transducin (beta)-like 1X-linked (TBL1X), mRNA.
Homo sapiens WW domain binding protein 5 (WBP5), transcript variant 4, mRNA.
Homo sapiens chromosome 14 open reading frame 181 (C14orf181), mRNA.
Homo sapiens tubulin folding cofactor D (TBCD), mRNA.
Homo sapiens threonyl-tRNA synthetase 2, mitochondrial (putative) (TARS2), nuclear gene encoding mitochondria
Homo sapiens TYRO protein tyrosine kinase binding protein (TYROBP), transcript variant 1, mRNA.
Homo sapiens complement component 5 (C5), mRNA.
qw67g08.x1 NCI_CGAP_Ov33 Homo sapiens cDNA clone IMAGE:1996190 3, mRNA sequence
Homo sapiens Sec23 homolog B (S. cerevisiae) (SEC23B), transcript variant 2, mRNA.
Homo sapiens metallothionein 1F (MT1F), mRNA.
Homo sapiens X (inactive)-specific transcript (non-protein coding) (XIST), non-coding RNA.
Homo sapiens interleukin 3 receptor, alpha (low affinity) (IL3RA), mRNA.
Homo sapiens endothelial cell adhesion molecule (ESAM), mRNA.
Homo sapiens pim-2 oncogene (PIM2), mRNA.
Homo sapiens dyskeratosis congenita 1, dyskerin (DKC1), mRNA.
Homo sapiens hypothetical protein HSPC111 (HSPC111), mRNA.
AGENCOURT_14354957 NIH_MGC_191 Homo sapiens cDNA clone IMAGE:30413554 5, mRNA sequence
Homo sapiens small nucleolar RNA, C/D box 56 (SNORD56), small nuclear RNA.
Homo sapiens zinc finger, CCHC domain containing 6 (ZCCHC6), mRNA.
Homo sapiens GTPase, IMAP family member 4 (GIMAP4), mRNA.
Homo sapiens prostaglandin E receptor 4 (subtype EP4) (PTGER4), mRNA.
Homo sapiens KIAA0427 (KIAA0427), mRNA.
Homo sapiens erythrocyte membrane protein band 4.1-like 2 (EPB41L2), mRNA.
Homo sapiens utrophin (UTRN), mRNA.
Homo sapiens carbohydrate (N-acetylglucosamine 6-O) sulfotransferase 7 (CHST7), mRNA.
Homo sapiens TAF5 RNA polymerase II, TATA box binding protein (TBP)-associated factor, 100kDa (TAF5), mRNA.
Homo sapiens WD repeat domain 49 (WDR49), mRNA.
Homo sapiens membrane-spanning 4-domains, subfamily A, member 6A (MS4A6A), transcript variant 2, mRNA.
Homo sapiens mediator complex subunit 11 (MED11), mRNA.
Homo sapiens nuclear factor of kappa light polypeptide gene enhancer in B-cells inhibitor, alpha (NFKBIA), mRNA.
PREDICTED: Homo sapiens similar to beta-tubulin cofactor D isoform 1 (LOC654260), mRNA.
Homo sapiens epithelial membrane protein 3 (EMP3), mRNA.
Homo sapiens DEAH (Asp-Glu-Ala-His) box polypeptide 33 (DHX33), mRNA.
Homo sapiens ras homolog gene family, member B (RHOB), mRNA.
Homo sapiens clone 23700 mRNA sequence
Homo sapiens lymphocyte antigen 6 complex, locus E (LY6E), mRNA.
Homo sapiens DEP domain containing 6 (DEPDC6), mRNA.
Homo sapiens heparin-binding EGF-like growth factor (HBEGF), mRNA.
Homo sapiens coiled-coil domain containing 99 (CCDC99), mRNA.
Homo sapiens metallothionein 1A (MT1A), mRNA.
Homo sapiens small nucleolar RNA, H/ACA box 61 (SNORA61), small nucleolar RNA.
Homo sapiens UDP-Gal:betaGlcNAc beta 1,4- galactosyltransferase, polypeptide 5 (B4GALT5), mRNA.
Homo sapiens RAB20, member RAS oncogene family (RAB20), mRNA.
Homo sapiens TSR1, 20S rRNA accumulation, homolog (S. cerevisiae) (TSR1), mRNA.

Homo sapiens plasminogen activator, urokinase (PLAU), mRNA.
PREDICTED: Homo sapiens hypothetical protein LOC727820 (LOC727820), mRNA.
PREDICTED: Homo sapiens misc_RNA (C10orf50), miscRNA.
Homo sapiens chromosome 5 open reading frame 33 (C5orf33), transcript variant 2, mRNA.
PREDICTED: Homo sapiens similar to general transcription factor II, i isoform 1 (LOC652771), mRNA.
Homo sapiens KIAA1279 (KIAA1279), mRNA.
Homo sapiens metallothionein E (MTE), mRNA.
Homo sapiens toll-like receptor adaptor molecule 1 (TICAM1), transcript variant 2, mRNA.
Homo sapiens interleukin 10 receptor, alpha (IL10RA), mRNA.
Homo sapiens FK506 binding protein 1A, 12kDa (FKBP1A), transcript variant 12B, mRNA.
PREDICTED: Homo sapiens misc_RNA (LOC729680), miscRNA.
PREDICTED: Homo sapiens similar to high-mobility group box 3 (LOC646993), mRNA.
Homo sapiens phosphoglucomutase 2 (PGM2), mRNA.
PREDICTED: Homo sapiens misc_RNA (LOC100128899), miscRNA.
Homo sapiens dendrin (DDN), mRNA.
Homo sapiens v-ets erythroblastosis virus E26 oncogene homolog 1 (avian) (ETS1), mRNA.
Homo sapiens proline-rich coiled-coil 1 (PRRC1), mRNA.
Homo sapiens ADP-ribosylation factor-like 9 (ARL9), mRNA.
Homo sapiens hypothetical LOC100188949 (LOC100188949), non-coding RNA.
Homo sapiens zinc fingers and homeoboxes 2 (ZHX2), mRNA.
Homo sapiens EF-hand domain (C-terminal) containing 2 (EFHC2), mRNA.
Homo sapiens transmembrane protein 2 (TMEM2), mRNA.
Homo sapiens zinc finger protein 185 (LIM domain) (ZNF185), mRNA.
Homo sapiens GTPase activating protein (SH3 domain) binding protein 1 (G3BP1), transcript variant 2, mRNA.
Homo sapiens tryptophanyl tRNA synthetase 2, mitochondrial (WARS2), nuclear gene encoding mitochondrial pro
Homo sapiens KN motif and ankyrin repeat domains 2 (KANK2), mRNA.
Homo sapiens TRAF-interacting protein with forkhead-associated domain (TIFA), mRNA.
Homo sapiens hexokinase 2 (HK2), mRNA.
Homo sapiens tetratricopeptide repeat domain 39C (TTC39C), mRNA.
Homo sapiens transcription factor 7-like 2 (T-cell specific, HMG-box) (TCF7L2), mRNA.
Homo sapiens transforming growth factor, beta receptor III (TGFBR3), mRNA.
Homo sapiens phosphoribosylformylglycinamidine synthase (FGAR amidotransferase) (PFAS), mRNA.
Homo sapiens basic, immunoglobulin-like variable motif containing (BIVM), mRNA.
PREDICTED: Homo sapiens similar to rcTPI1, transcript variant 1 (LOC729708), mRNA.
Homo sapiens cell division cycle 2, G1 to S and G2 to M (CDC2), transcript variant 1, mRNA.
Homo sapiens chromosome 11 open reading frame 82 (C11orf82), mRNA.
Homo sapiens BCL2/adenovirus E1B 19kDa interacting protein 3 (BNIP3), nuclear gene encoding mitochondrial pro
Homo sapiens calcium homeostasis endoplasmic reticulum protein (CHERP), mRNA.
Homo sapiens 5'-nucleotidase, ecto (CD73) (NT5E), mRNA.
Homo sapiens syndecan binding protein (syntenin) (SDCBP), transcript variant 2, mRNA.
Homo sapiens SEH1-like (S. cerevisiae) (SEH1L), transcript variant 1, mRNA.
Homo sapiens cerebellin 3 precursor (CBLN3), mRNA.
Homo sapiens trophoblast glycoprotein (TPBG), mRNA.
Homo sapiens glucosaminyl (N-acetyl) transferase 1, core 2 (beta-1,6-N-acetylglucosaminyltransferase) (GCNT1), r
Homo sapiens hepatoma-derived growth factor, related protein 3 (HDGFRP3), mRNA.
Homo sapiens family with sequence similarity 65, member B (FAM65B), transcript variant 2, mRNA.
Homo sapiens mitogen-activated protein kinase kinase 4 (MAP2K4), mRNA.

Homo sapiens DENN/MADD domain containing 3 (DENND3), mRNA.
Homo sapiens IQ motif containing G (IQCG), mRNA.
Homo sapiens dynein, light chain, LC8-type 2 (DYNLL2), mRNA.
Homo sapiens nexilin (F actin binding protein) (NEXN), mRNA.
Homo sapiens tRNA nucleotidyl transferase, CCA-adding, 1 (TRNT1), mRNA.
Homo sapiens chromosome 18 open reading frame 19 (C18orf19), mRNA.
Homo sapiens leptin receptor overlapping transcript-like 1 (LEPROTL1), mRNA.
Homo sapiens growth arrest and DNA-damage-inducible, alpha (GADD45A), mRNA.
Homo sapiens methylenetetrahydrofolate dehydrogenase (NADP+ dependent) 2-like (MTHFD2L), mRNA.
Homo sapiens fucosidase, alpha-L- 1, tissue (FUCA1), mRNA.
Homo sapiens transcription factor AP-4 (activating enhancer binding protein 4) (TFAP4), mRNA.
Homo sapiens immunoglobulin J polypeptide, linker protein for immunoglobulin alpha and mu polypeptides (IGJ),
Homo sapiens family with sequence similarity 44, member A (FAM44A), mRNA.
Homo sapiens solute carrier family 39 (zinc transporter), member 14 (SLC39A14), mRNA.
17000532611997 GRN_EB Homo sapiens cDNA 5, mRNA sequence
Homo sapiens mitogen-activated protein kinase kinase kinase 5 (MAP3K5), mRNA.
Homo sapiens sirtuin (silent mating type information regulation 2 homolog) 1 (S. cerevisiae) (SIRT1), mRNA.
Homo sapiens MOB1, Mps One Binder kinase activator-like 1A (yeast) (MOBKL1A), mRNA.
Homo sapiens cat eye syndrome chromosome region, candidate 6 (CECR6), mRNA.
Homo sapiens acyl-CoA synthetase long-chain family member 1 (ACSL1), mRNA.
Homo sapiens B-box and SPRY domain containing (BSPRY), mRNA.
Homo sapiens cDNA FLJ13598 fis, clone PLACE1009921
PREDICTED: Homo sapiens Kruppel-like factor 11 (KLF11), mRNA.
Homo sapiens STAM binding protein-like 1 (STAMBPL1), mRNA.
Homo sapiens family with sequence similarity 101, member B (FAM101B), mRNA.
Homo sapiens nephronophthisis 4 (NPHP4), mRNA.
Homo sapiens RNA (guanine-7-) methyltransferase (RNMT), mRNA.
Homo sapiens immediate early response 3 interacting protein 1 (IER3IP1), mRNA.
Homo sapiens asparagine-linked glycosylation 3 homolog (S. cerevisiae, alpha-1,3-mannosyltransferase) (ALG3), m
Homo sapiens cDNA FLJ33115 fis, clone TRACH2001314
Homo sapiens growth factor independent 1 transcription repressor (GFI1), mRNA.
Homo sapiens zinc finger protein, multitype 1 (ZFPM1), mRNA.
Homo sapiens killer cell lectin-like receptor subfamily F, member 1 (KLRF1), mRNA.
Homo sapiens MIR155 host gene (non-protein coding) (MIR155HG), non-coding RNA.
Homo sapiens general transcription factor IIIA (GTF3A), mRNA.
Homo sapiens RANBP2-like and GRIP domain containing 2 (RGPD2), mRNA. XM_001134112 XM_001134114 XM_0
Homo sapiens tumor necrosis factor (ligand) superfamily, member 13b (TNFSF13B), transcript variant 1, mRNA.
Homo sapiens frequently rearranged in advanced T-cell lymphomas 2 (FRAT2), mRNA.
Homo sapiens peroxiredoxin 3 (PRDX3), nuclear gene encoding mitochondrial protein, transcript variant 1, mRNA.
Homo sapiens similar to LINE-1 reverse transcriptase homolog (LOC401623), mRNA.
PREDICTED: Homo sapiens hypothetical protein BC006130 (LOC93622), misc RNA.
Homo sapiens ELL associated factor 2 (EAF2), mRNA.
Homo sapiens glycine dehydrogenase (decarboxylating) (GLDC), mRNA.
PREDICTED: Homo sapiens RANBP2-like and GRIP domain containing 8 (RGPD8), mRNA.
Homo sapiens regulator of G-protein signaling 18 (RGS18), mRNA.
Homo sapiens kinesin family member 11 (KIF11), mRNA.
Homo sapiens synaptic vesicle glycoprotein 2A (SV2A), mRNA.

Homo sapiens immediate early response 5 (IER5), mRNA.
Homo sapiens radial spoke 3 homolog (Chlamydomonas) (RSPH3), mRNA.
Homo sapiens germinal center expressed transcript 2 (GCET2), transcript variant 1, mRNA.
Homo sapiens carbohydrate (N-acetylglucosamine-6-O) sulfotransferase 2 (CHST2), mRNA.
Homo sapiens dihydrolipoamide S-acetyltransferase (DLAT), mRNA.
Homo sapiens sushi domain containing 2 (SUSD2), mRNA.
Homo sapiens quinoid dihydropteridine reductase (QDPR), mRNA.
Homo sapiens hypothetical protein LOC90925 (LOC90925), mRNA.
Homo sapiens deoxyribonuclease II, lysosomal (DNASE2), mRNA.
Homo sapiens glutamate-cysteine ligase, modifier subunit (GCLM), mRNA.
Homo sapiens CUG triplet repeat, RNA binding protein 1 (CUGBP1), transcript variant 2, mRNA.
Homo sapiens delta-like 1 (Drosophila) (DLL1), mRNA.
Homo sapiens lipocalin 2 (LCN2), mRNA.
Homo sapiens zinc finger protein 792 (ZNF792), mRNA.
Homo sapiens coronin, actin binding protein, 1C (CORO1C), transcript variant 1, mRNA.
PREDICTED: Homo sapiens hypothetical protein LOC730525 (LOC730525), mRNA.
Homo sapiens OAF homolog (Drosophila) (OAF), mRNA.
Homo sapiens elastin microfibril interfacer 2 (EMILIN2), mRNA.
Homo sapiens ribonucleotide reductase M2 polypeptide (RRM2), mRNA.
PREDICTED: Homo sapiens similar to RIKEN cDNA 2310039H08 (LOC441150), mRNA.
Homo sapiens zinc finger protein 721 (ZNF721), mRNA.
Homo sapiens progesterone receptor membrane component 1 (PGRMC1), mRNA.
Homo sapiens teashirt zinc finger homeobox 1 (TSHZ1), mRNA.
Homo sapiens cytoplasmic linker associated protein 2 (CLASP2), mRNA.
Homo sapiens F-box protein 34 (FBXO34), mRNA.
Homo sapiens chromosome 19 open reading frame 42 (C19orf42), mRNA.
PREDICTED: Homo sapiens fatty acid binding protein 5-like 2 (FABP5L2), mRNA.
Homo sapiens CD93 molecule (CD93), mRNA.
Homo sapiens CD40 molecule, TNF receptor superfamily member 5 (CD40), transcript variant 1, mRNA.
Homo sapiens nuclear receptor co-repressor 1 (NCOR1), mRNA.
Homo sapiens mitochondrial ribosomal protein L19 (MRPL19), nuclear gene encoding mitochondrial protein, mRN
PREDICTED: Homo sapiens similar to CHRNA7 (cholinergic receptor, nicotinic, alpha 7, exons 5-10) and FAM7A (fa
Homo sapiens family with sequence similarity 100, member B (FAM100B), mRNA.
Homo sapiens ring finger protein 141 (RNF141), mRNA.
Homo sapiens carbonyl reductase 1 (CBR1), mRNA.
Homo sapiens CD27 molecule (CD27), mRNA.
Homo sapiens topoisomerase (DNA) II alpha 170kDa (TOP2A), mRNA.
Homo sapiens leucine rich repeat containing 58 (LRRC58), mRNA.
Homo sapiens membrane-spanning 4-domains, subfamily A, member 1 (MS4A1), transcript variant 1, mRNA.
Homo sapiens intercellular adhesion molecule 3 (ICAM3), mRNA.
Homo sapiens cDNA FLJ41455 fis, clone BRSTN2012284
Homo sapiens sperm associated antigen 5 (SPAG5), mRNA.
Homo sapiens apoptotic peptidase activating factor 1 (APAF1), transcript variant 1, mRNA.
Homo sapiens carcinoembryonic antigen-related cell adhesion molecule 6 (non-specific cross reacting antigen) (C
Homo sapiens inhibitor of DNA binding 2, dominant negative helix-loop-helix protein (ID2), mRNA.
Homo sapiens chromosome 5 open reading frame 30 (C5orf30), mRNA.
Homo sapiens protein phosphatase 2C, magnesium-dependent, catalytic subunit (PPM2C), nuclear gene encoding

Homo sapiens leucine rich repeat containing 28 (LRRC28), mRNA.
Homo sapiens integrin, beta 7 (ITGB7), mRNA.
Homo sapiens phosphoribosyl pyrophosphate amidotransferase (PPAT), mRNA.
Homo sapiens interferon regulatory factor 1 (IRF1), mRNA.
PREDICTED: Homo sapiens hypothetical protein LOC650919 (LOC650919), mRNA.
Homo sapiens general transcription factor II, i, pseudogene 1 (GTF2IP1) on chromosome 7.
Homo sapiens nerve growth factor receptor (TNFRSF16) associated protein 1 (NGFRAP1), transcript variant 1, mR
Homo sapiens dehydrogenase/reductase (SDR family) member 3 (DHRS3), mRNA.
Homo sapiens Abelson helper integration site 1 (AHI1), mRNA.
Homo sapiens membrane-associated ring finger (C3HC4) 3 (MARCH3), mRNA.
Homo sapiens ASF1 anti-silencing function 1 homolog B (S. cerevisiae) (ASF1B), mRNA.
Homo sapiens UBX domain protein 8 (UBXN8), mRNA.
Homo sapiens regulator of G-protein signalling 16 (RGS16), mRNA.
Homo sapiens N-myc downstream regulated gene 1 (NDRG1), mRNA.
Homo sapiens SRY (sex determining region Y)-box 8 (SOX8), mRNA.
Homo sapiens potassium channel, subfamily K, member 6 (KCNK6), mRNA.
Homo sapiens piggyBac transposable element derived 4 (PGBD4), mRNA.
Homo sapiens phosphatidylinositol glycan anchor biosynthesis, class M (PIGM), mRNA.
Homo sapiens pseudouridylate synthase 7 homolog (S. cerevisiae) (PUS7), mRNA.
PREDICTED: Homo sapiens hypothetical protein LOC100133077 (LOC100133077), mRNA.
Homo sapiens UDP-N-acteylglucosamine pyrophosphorylase 1 (UAP1), mRNA.
Homo sapiens pleiomorphic adenoma gene-like 1 (PLAGL1), transcript variant 4, mRNA.
Homo sapiens proline rich 7 (synaptic) (PRR7), mRNA.
Homo sapiens SMAD family member 7 (SMAD7), mRNA.
Homo sapiens caldesmon 1 (CALD1), transcript variant 3, mRNA.
Homo sapiens COMM domain containing 10 (COMMD10), mRNA.
PREDICTED: Homo sapiens hypothetical protein LOC643985 (LOC643985), mRNA.
Homo sapiens caveolin 1, caveolae protein, 22kDa (CAV1), mRNA.
PREDICTED: Homo sapiens similar to H2A histone family, member X (LOC731314), mRNA.
Homo sapiens zinc finger and SCAN domain containing 5A (ZSCAN5A), mRNA.
Homo sapiens chromosome 6 open reading frame 203 (C6orf203), mRNA.
Homo sapiens BAH domain and coiled-coil containing 1 (BAHCC1), mRNA.
Homo sapiens golgi autoantigen, golgin subfamily a, 4 (GOLGA4), mRNA.
Homo sapiens PAP associated domain containing 5 (PAPD5), transcript variant 2, mRNA.
Homo sapiens zinc finger CCCH-type containing 18 (ZC3H18), mRNA.
Homo sapiens adiponectin receptor 2 (ADIPOR2), mRNA.
Homo sapiens general transcription factor IIH, polypeptide 2, 44kDa (GTF2H2), mRNA.
Homo sapiens forkhead box H1 (FOXH1), mRNA.
Homo sapiens RING1 and YY1 binding protein (RYBP), mRNA.
Homo sapiens serine palmitoyltransferase, long chain base subunit 2 (SPTLC2), mRNA.
Homo sapiens dual adaptor of phosphotyrosine and 3-phosphoinositides (DAPP1), mRNA.
Homo sapiens B-cell CLL/lymphoma 3 (BCL3), mRNA.
Homo sapiens p21/Cdc42/Rac1-activated kinase 1 (STE20 homolog, yeast) (PAK1), mRNA.
Homo sapiens colony stimulating factor 1 receptor, formerly McDonough feline sarcoma viral (v-fms) oncogene h
Homo sapiens hypothetical protein (LOC387882), mRNA.
Homo sapiens metallothionein 2A (MT2A), mRNA.
Homo sapiens KIAA1430 (KIAA1430), mRNA.

Homo sapiens centromere protein E, 312kDa (CENPE), mRNA.
Homo sapiens INO80 complex subunit C (INO80C), transcript variant 2, mRNA.
Homo sapiens centaurin, gamma 2 (CENTG2), mRNA.
Homo sapiens ral guanine nucleotide dissociation stimulator-like 4 (RGL4), mRNA.
Homo sapiens chromosome 14 open reading frame 169 (C14orf169), mRNA.
Homo sapiens RUN and FYVE domain containing 3 (RUFY3), transcript variant 2, mRNA.
Homo sapiens asp (abnormal spindle) homolog, microcephaly associated (Drosophila) (ASPM), mRNA.
Homo sapiens ArfGAP with SH3 domain, ankyrin repeat and PH domain 2 (ASAP2), transcript variant 1, mRNA.
Homo sapiens peptidylglycine alpha-amidating monooxygenase (PAM), transcript variant 3, mRNA.
Homo sapiens GM2 ganglioside activator (GM2A), mRNA.
Homo sapiens inducible T-cell co-stimulator ligand (ICOSLG), mRNA.
Homo sapiens pre-B lymphocyte gene 3 (VPREB3), mRNA.
Homo sapiens chromosome 5 open reading frame 39 (C5orf39), mRNA.
Homo sapiens coiled-coil domain containing 102A (CCDC102A), mRNA.
Homo sapiens chromosome 3 open reading frame 21 (C3orf21), mRNA.
Homo sapiens exostoses (multiple) 1 (EXT1), mRNA.
Homo sapiens vaccinia related kinase 1 (VRK1), mRNA.
Homo sapiens adrenergic, alpha-2A-, receptor (ADRA2A), mRNA.
Homo sapiens Cdk5 and Abl enzyme substrate 1 (CABLES1), transcript variant 1, mRNA.
Homo sapiens hyaluronan-mediated motility receptor (RHAMM) (HMMR), transcript variant 2, mRNA.
PREDICTED: Homo sapiens hypothetical protein LOC642299 (LOC642299), mRNA.
Homo sapiens villin 2 (ezrin) (VIL2), mRNA.
Homo sapiens FAST kinase domains 2 (FASTKD2), mRNA.
Homo sapiens G protein-coupled receptor kinase 5 (GRK5), mRNA.
Homo sapiens coatomer protein complex, subunit gamma 2 (COPG2), mRNA.
Homo sapiens sprouty-related, EVH1 domain containing 2 (SPRED2), mRNA.
Homo sapiens BUB1 budding uninhibited by benzimidazoles 1 homolog (yeast) (BUB1), mRNA.
Homo sapiens centromere protein Q (CENPQ), mRNA.
Homo sapiens zinc finger, CCHC domain containing 24 (ZCCHC24), mRNA.
Homo sapiens mitogen-activated protein kinase kinase 1 (MAP2K1), mRNA.
Homo sapiens serine/threonine kinase 17b (STK17B), mRNA.
Homo sapiens ribosomal protein L39-like (RPL39L), mRNA.
Homo sapiens G protein-coupled receptor 146 (GPR146), mRNA.
Homo sapiens metallo-beta-lactamase domain containing 2 (MBLAC2), mRNA.
Homo sapiens hypothetical protein LOC283932 (LOC283932), mRNA.
PREDICTED: Homo sapiens similar to FK506-binding protein 1A (LOC642489), mRNA.
Homo sapiens KDEL (Lys-Asp-Glu-Leu) endoplasmic reticulum protein retention receptor 2 (KDELR2), transcript va
Homo sapiens chromosome 1 open reading frame 69 (C1orf69), mRNA.
Homo sapiens thyroid hormone receptor interactor 6 (TRIP6), mRNA.
Homo sapiens chromosome 9 open reading frame 140 (C9orf140), mRNA.
Homo sapiens cell division cycle associated 7-like (CDCA7L), mRNA.
Homo sapiens glycerol-3-phosphate dehydrogenase 1-like (GPD1L), mRNA.
Homo sapiens protein disulfide isomerase family A, member 5 (PDIA5), mRNA.
Homo sapiens development and differentiation enhancing factor 2 (DDEF2), mRNA.
PREDICTED: Homo sapiens similar to hydroxysteroid (17-beta) dehydrogenase 7 (LOC651621), mRNA.
Homo sapiens peter pan homolog (Drosophila) (PPAN), mRNA.
Homo sapiens chimerin (chimaerin) 2 (CHN2), transcript variant 2, mRNA.

Homo sapiens sorting nexin 4 (SNX4), mRNA.
Homo sapiens ubiquitin-conjugating enzyme E2Q (putative) 1 (UBE2Q1), mRNA.
Homo sapiens regulator of G-protein signaling 1 (RGS1), mRNA.
Homo sapiens hypothetical protein LOC201175 (LOC201175), mRNA.
Homo sapiens 2'-5'-oligoadenylate synthetase 2, 69/71kDa (OAS2), transcript variant 2, mRNA.
Homo sapiens major histocompatibility complex, class II, DR beta 6 (pseudogene) (HLA-DRB6), non-coding RNA.
Homo sapiens solute carrier family 25, member 43 (SLC25A43), mRNA.
Homo sapiens glucuronic acid epimerase (GLCE), mRNA.
Homo sapiens fatty acyl CoA reductase 2 (FAR2), mRNA.
Homo sapiens lemur tyrosine kinase 2 (LMTK2), mRNA.
PREDICTED: Homo sapiens similar to membrane-associated ring finger (C3HC4) 3 (LOC100133609), mRNA.
Homo sapiens nephroblastoma overexpressed gene (NOV), mRNA.
Homo sapiens solute carrier family 25 (mitochondrial carrier; adenine nucleotide translocator), member 4 (SLC25A
Homo sapiens interferon gamma receptor 1 (IFNGR1), mRNA.
Homo sapiens progesterone receptor membrane component 2 (PGRMC2), mRNA.
Homo sapiens ATPase, class I, type 8B, member 4 (ATP8B4), mRNA.
Homo sapiens Friend leukemia virus integration 1 (FLI1), mRNA.
PREDICTED: Homo sapiens misc_RNA (LOC100129960), miscRNA.
Homo sapiens ecotropic viral integration site 2B (EVI2B), mRNA.
Homo sapiens WD repeat domain 40A (WDR40A), mRNA.
Homo sapiens Rho guanine nucleotide exchange factor (GEF) 17 (ARHGEF17), mRNA.
Homo sapiens syntaxin 2 (STX2), transcript variant 2, mRNA.
Homo sapiens fasciculation and elongation protein zeta 2 (zygin II) (FEZ2), transcript variant 1, mRNA.
Homo sapiens RYK receptor-like tyrosine kinase (RYK), transcript variant 1, mRNA.
Homo sapiens chromosome 9 open reading frame 119 (C9orf119), mRNA.
Homo sapiens transferrin (TF), mRNA.
Homo sapiens solute carrier family 12 (sodium/potassium/chloride transporters), member 2 (SLC12A2), mRNA.
Homo sapiens coiled-coil-helix-coiled-coil-helix domain containing 7 (CHCHD7), transcript variant 5, mRNA.
Homo sapiens phosducin-like (PDCL), mRNA.
Homo sapiens leucine rich repeat containing 56 (LRRC56), mRNA.
Homo sapiens phosphatidylinositol transfer protein, alpha (PITPNA), mRNA.
Homo sapiens lymphoid-restricted membrane protein (LRMP), mRNA.
Homo sapiens LIM domain binding 2 (LDB2), mRNA.
Homo sapiens neutrophil cytosolic factor 1 (NCF1), mRNA.
Homo sapiens MAK16 homolog (S. cerevisiae) (MAK16), mRNA.
Homo sapiens toll-like receptor 1 (TLR1), mRNA.
PREDICTED: Homo sapiens similar to Fatty acid-binding protein, epidermal (E-FABP) (Psoriasis-associated fatty acid
Homo sapiens mitochondrial fission regulator 1 (MTFR1), nuclear gene encoding mitochondrial protein, mRNA.
Homo sapiens centromere protein F, 350/400ka (mitosin) (CENPF), mRNA.
Homo sapiens methionyl-tRNA synthetase 2, mitochondrial (MARS2), nuclear gene encoding mitochondrial protei
PREDICTED: Homo sapiens similar to hCG2040254 (LOC100133697), mRNA.
Homo sapiens FP944 (LOC286367), non-coding RNA.
Homo sapiens tetraspanin 33 (TSPAN33), mRNA.
Homo sapiens elongation factor, RNA polymerase II, 2 (ELL2), mRNA.
Homo sapiens tetratricopeptide repeat domain 7A (TTC7A), mRNA.
Homo sapiens legumain (LGMN), transcript variant 2, mRNA.
Homo sapiens solute carrier family 45, member 3 (SLC45A3), mRNA.

Homo sapiens integrin, beta 2 (antigen CD18 (p95), lymphocyte function-associated antigen 1; macrophage antige
Homo sapiens tribbles homolog 1 (Drosophila) (TRIB1), mRNA.
Homo sapiens hypothetical LOC791120 (LOC791120), non-coding RNA.
BX115738 Soares melanocyte 2NbHM Homo sapiens cDNA clone IMAGp998D07589, mRNA sequence
Homo sapiens solute carrier family 6, member 16 (SLC6A16), mRNA.
Homo sapiens serpin peptidase inhibitor, clade I (neuroserpin), member 1 (SERPINI1), mRNA.
Homo sapiens NIMA (never in mitosis gene a)-related kinase 2 (NEK2), mRNA.
Homo sapiens hypothetical LOC339541 (MGC33556), mRNA.
Homo sapiens calcium channel, voltage-dependent, beta 3 subunit (CACNB3), mRNA.
Homo sapiens Fas (TNFRSF6)-associated via death domain (FADD), mRNA.
Homo sapiens 3-hydroxy-3-methylglutaryl-Coenzyme A synthase 1 (soluble) (HMGCS1), transcript variant 2, mRNA
Homo sapiens centrosomal protein 72kDa (CEP72), mRNA.
Homo sapiens angiopoietin 1 (ANGPT1), mRNA.
Homo sapiens arginyl-tRNA synthetase (RARS), mRNA.
Homo sapiens testis expressed 10 (TEX10), mRNA.
Homo sapiens carnitine palmitoyltransferase II (CPT2), nuclear gene encoding mitochondrial protein, mRNA.
Homo sapiens THO complex 3 (THOC3), mRNA.
Homo sapiens zinc finger protein 318 (ZNF318), mRNA.
Homo sapiens heparan sulfate 2-O-sulfotransferase 1 (HS2ST1), mRNA.
Homo sapiens sidekick homolog 2 (chicken) (SDK2), mRNA.

NA.

l protein, transcript variant 1, mRNA.

PAT5), mRNA.

RNA.

ninotransferase) (LOC648399), mRNA.

ı.

nRNA.

2, mRNA.

ıRNA.

al protein, mRNA.

mRNA.

tein, transcript variant 2, mRNA.

otein, mRNA.

mRNA.

mRNA.

ıRNA.

)01134116

IA.
mily with sequence similarity 7A, exons A-E) fusion (LOC727935), mRNA.

EACAM6), mRNA.

mitochondrial protein, mRNA.

NA.

omolog (CSF1R), mRNA.

riant 1, mRNA.

\4), nuclear gene encoding mitochondrial protein, mRNA.

d-binding protein homolog) (PA-FABP) (LOC387934), mRNA.

n, mRNA.

en 1 (mac-1) beta subunit) (ITGB2), mRNA.

A.
