# Supplementary material for: High expression of ID family and IGJ genes signature as predictor of low induction treatment response and worst survival in adult Hispanic patients with B-acute lymphoblastic leukemia
Source: J Exp Clin Cancer Res. 2016 Apr 5;35:64. doi: 10.1186/s13046-016-0333-z (PMC4820984; doi:10.1186/s13046-016-0333-z)
Supplement: Additional file 5: — Complete file derived from pathway analysis in patients who failed to remission therapy. Signaling analysis was done using MetaCore KPA. File shows 10 key pathways dysregulated in this group of patients. (PDF 1174 kb) [file 13046_2016_333_MOESM5_ESM.pdf]

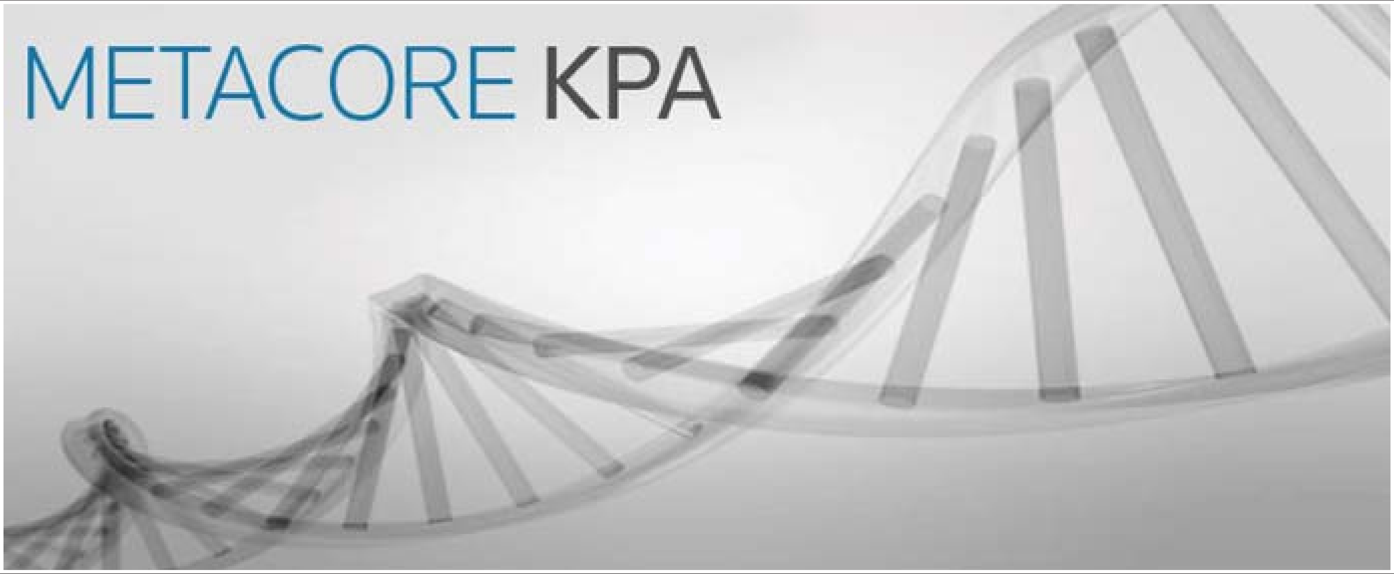

noRemision

05 NOV 2015

CONTENTS

INTRODUCTION 3

INPUT DATA AND SETTINGS 4

RESULTS 5

- Key Pathway Maps 5
- Diseases (by Biomarkers) 30
- Process Networks 32
- Map Folders 33

Appendix 1: Legend 34

Appendix 2: Glossary 35

Appendix 3: List of Key Hubs IDs 37

INTRODUCTION

In order to understand functional processes behind the differentially expressed gene (DEG) list, KPA represents comprehensive pathway analysis workflow.

The Causal Reasoning approach and Overconnectivity analysis help identify both key direct regulators (i.e. one step away) of the dataset and the major “master” regulators of the global protein network. Concurrent enrichment analysis of both differentially expressed genes and their ranked direct and indirect regulators (Key Hubs), allows one to reconstruct the mechanisms underlining differential gene expression more comprehensively.

We define “Key Ontology Processes” as ontology terms (i.e. pathway maps) enriched with both differentially expressed genes and corresponding Key Hubs. They are identified by the following workflow.

1. Enrichment analysis is performed for the list of differentially expressed genes. Statistically significant ontology processes (enrichment p-value < 0.01) for differentially expressed genes are identified.
2. Calculation of Key Hubs by either Causal Reasoning approach (if DEGs associated with expression values) or Overconnectivity analysis (if DEGs uploaded without expression values).
3. Enrichment analysis is performed for the corresponding list of Key Hubs. Statistically significant ontology processes (enrichment p-value < 0.01) for Key Hubs are identified.
4. Ontology processes statistically significant for both the list of differentially expressed genes and the list of corresponding Key Hubs are identified.
5. Ontology processes that display “synergistic” behaviour for the list of differentially expressed genes and the list of corresponding Key Hubs are defined (please see “Enrichment synergy” in Glossary). The final list of synergistic ontology processes includes all ontology terms with synergistic expression pattern for the union of DEGs and Key Hubs and p-value < 0.01.
6. The resulted list of key processes includes ontology terms which show significant enrichment for both lists and synergistic behaviour.

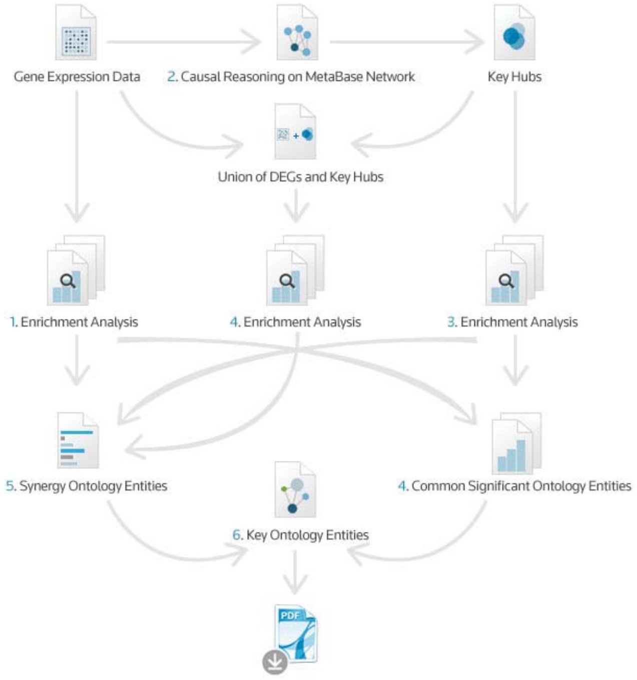

INPUT DATA AND SETTINGS

This section contains experiment file name and data format. Statistics section contains Ids that were in original file and how many of them were recognized by system and mapped on Network Objects. Analysis settings that were used for Key Hubs and Processes calculation steps are listed in the last section.

Analysis Overview	
Analysis Name	noRemision
File Type	Gene Expression
File Content	Tag ID: Gene Symbol, Fold change
KPA Version	2.0

Statistics

IDs in File	104
Network objects	107
Unrecognized IDs	16

Analysis Settings

Selected Processes Ontologies	Key Pathway Maps, Diseases (by Biomarkers), Process Networks, Map Folders
Key Processes p-value Threshold	0.01
Key Hubs Calculation Algorithm	Causal Reasoning Analysis
Key Hubs p-value Threshold	0.01

! This report contains only top 100 key results for each ontology.

RESULTS

Key Pathway Maps

Pathway maps are graphic images representing complete biochemical pathways or signaling cascades in a commonly accepted sense. All maps listed below are enriched with both input genes and Key Hubs.

Key Pathway Maps Details [10 processes]				
#	Name	Input Objects ▲ p-value	Key Hubs p-value	Union Objects p-value
1	Immune response_Role of PKR in stress-induced antiviral cell response	5.727E-6	0.003018	1.51E-7
2	Apoptosis and survival_Role of PKR in stress-induced apoptosis	9.695E-5	0.00231	1.09E-6
3	Immune response_CD40 signaling	2.153E-4	0.004851	5.329E-6
4	SLE genetic marker-specific pathways in antigen-presenting cells (APC)	5.76E-4	0.001901	4.378E-6
5	Immune response_IL-9 signaling pathway	5.84E-4	5.33E-4	8.799E-7
6	Immune response_TNF-R2 signaling pathways	0.001128	0.001254	4.272E-6
7	Immune response_Bacterial infections in normal airways	0.001445	0.001726	7.689E-6
8	Development_Role of G-CSF in hematopoietic stem cell mobilization	0.004112	0.001325	2.42E-4
9	Development_G-CSF-induced myeloid differentiation	0.008297	0.003777	9.974E-4
10	Signal transduction_Additional pathways of NF-kB activation (in the nucleus)	0.008297	1.985E-8	3.392E-10

Maps and Descriptions [1 of 10]

Name	Input Objects ▲ p-value	Key Hubs p-value	Union Objects p-value
Immune response_Role of PKR in stress-induced antiviral cell response	5.727E-6	0.003018	1.51E-7

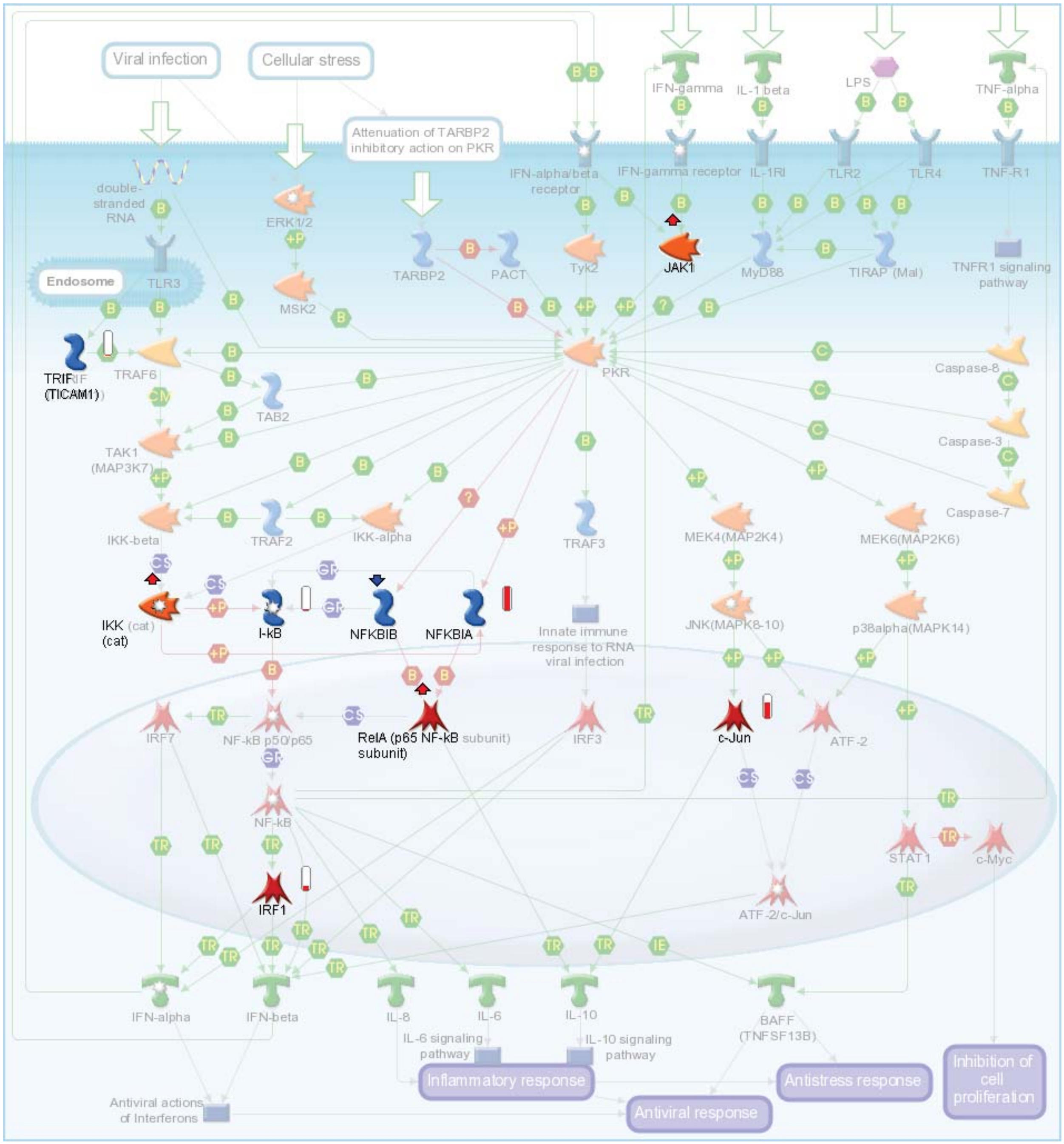

Abstract:

Activation of **double-stranded RNA**-dependent **PKR** by viral infections, extracellular stress signals and/or proinflammatory stimuli, including **LPS**, **TNF-alpha**, **IFN-gamma** and **IL-1 beta**, results in triggering of signaling pathways, which regulate cell antiviral and antistress response to pathogens and other cellular stress signals.

Details:

The interferon-induced **double-stranded RNA**-dependent protein kinase **PKR** is a member of a small family of serine-threonine kinases that are activated by extracellular stresses, mainly viral infections (see: viral process). **PKR** is activated by a variety of activating stimuli [1], [2]. The classical activator of **PKR** is **double-stranded RNA** (dsRNA), which directly binds **PKR** and triggers **PKR** kinase activity [3].

PKR is also activated by both **TNF-alpha** and **IFN-gamma**, which act synergistically during a variety of biological responses to induce **NF-kB** signaling and the expression of genes, involved in immune response and inflammatory response [4], [5]. **IFN-gamma** induces activation of **PKR** via **IFN-gamma receptor**/ **JAK1** pathway [6], [7]. **TNF-alpha** probably activates **PKR** via **TNF-R1**-induced caspase signaling (e.g. **Caspase-8**, **Caspase-3** and/or **Caspase-7**) [8], [9], [10].

PKR is also implicated in toll-like receptor signaling pathway, which plays a fundamental role in pathogen recognition and activation of innate immunity. **LPS**-induced **TLR2** and **TLR4** activate **PKR** via **TIRAP (Mal)** and **MyD88** [11], [12]. **PKR** is also engaged in dsRNA-activated **TLR3**/ **TRIF (TICAM1)**/ **TRAF6**/ **TAK1(MAP3K7)**/ **TAB2** signaling [13], [14]. **IL-1 beta** can also activate **PKR** during antiviral response (see: defense response to virus), probably via **MyD88** [1], [11], [15], [16].

A wide range of different cellular stresses can activate **PKR** through **PACT**, independently of dsRNA or other molecules [17], [18]. **TARBP2**, which is able to inhibit both **PACT** and **PKR**, is repressed by stress stimuli [19]. Another pathway of **PKR** activation is **ERK1/2**/ **MSK2** signaling [20]. Mechanism of **PKR** activation includes its homodimerisation and autophosphorylation [21], [22].

Activated **PKR** triggers multiple signaling pathways, which regulate cell inflammatory response. **PKR** is required for **MEK6(MAP2K6)**/ **p38alpha (MAPK14)**/ **STAT1** and **p38alpha (MAPK14)**/ **ATF-2**/ **ATF-2/c-Jun** signaling [23], [24], [25], [26], [27], [28]. **STAT1** activates **BAFF(TNFSF13B)** expression [29], [30]. **STAT1** also suppresses the expression of **c-Myc**, thus inhibiting cell proliferation [31]. **PKR** also activates **MEK4(MAP2K4)**/ **JNK(MAPK8-10)**/ **ATF-2** and/or **c-Jun**/ **ATF-2/c-Jun** signaling [12], [23], [25], [27], [28]. Activated **ATF-2/c-Jun** launches the expression of **IFN-beta** [28], [32], [33]. Activated **c-Jun** subsequently activates the expression of **IL-10** [34], [35].

PKR stimulates the **RelA (p65 NF-kB subunit)**/ **NF-kB p50/p65**/ **NF-kB** signaling via degradation of **NFKBIA** and/or **NFKBIB**, the members of the **I-kB** protein family [5], [12], [14], [36], [37], [38], or by activation of **IKK-alpha** and **IKK-beta** subunits of the **IKK (cat)** complex [13], [39], [40]. **PKR** is also able to interact with **TRAF2**, which is suggested to activate downstream signal towards the **NF-kB** via **IKK-alpha** and **IKK-beta** [41], [42].

NF-kB-regulated genes are involved in mediating **PKR**-induced antiviral (see: defense response to virus) and antistress response. These are proinflammatory cytokines **TNF-alpha**, **IL-6**, **IL-10** [35], [43], [44], **IL-8** [45], **IFN-beta** [33], [46], **IFN-gamma** [47] and **BAFF(TNFSF13B)** [30]. **NF-kB** also activates the expression of **IRF1** and **IRF7** [6], [48], [49]. **TRAF3** associates with **PKR** in the **IRF3** signaling pathway [49], [50]. **IRF1**, **IRF3** and **IRF7** are the factors launching **IFN-alpha** and **IFN-beta** expression [33], [51]. Thus, **PKR** is one of the first activators of **IFN-alpha** and **IFN-beta** expression in response to infection [51]. **IFN-alpha** and **IFN-beta** promote antiviral response (see: defense response to virus), thus amplifying inflammatory cell signaling in response to viral infection (see: viral process) [52]. Furthermore, **IFN-alpha** and **IFN-beta** are able to activate **PKR** via **IFN-alpha/beta receptor**/ **JAK1**/ **Tyk2** signaling [7].

Maps and Descriptions [2 of 10]

Name	Input Objects ▲ p-value	Key Hubs p-value	Union Objects p-value
Apoptosis and survival_Role of PKR in stress-induced apoptosis	9.695E-5	0.00231	1.09E-6

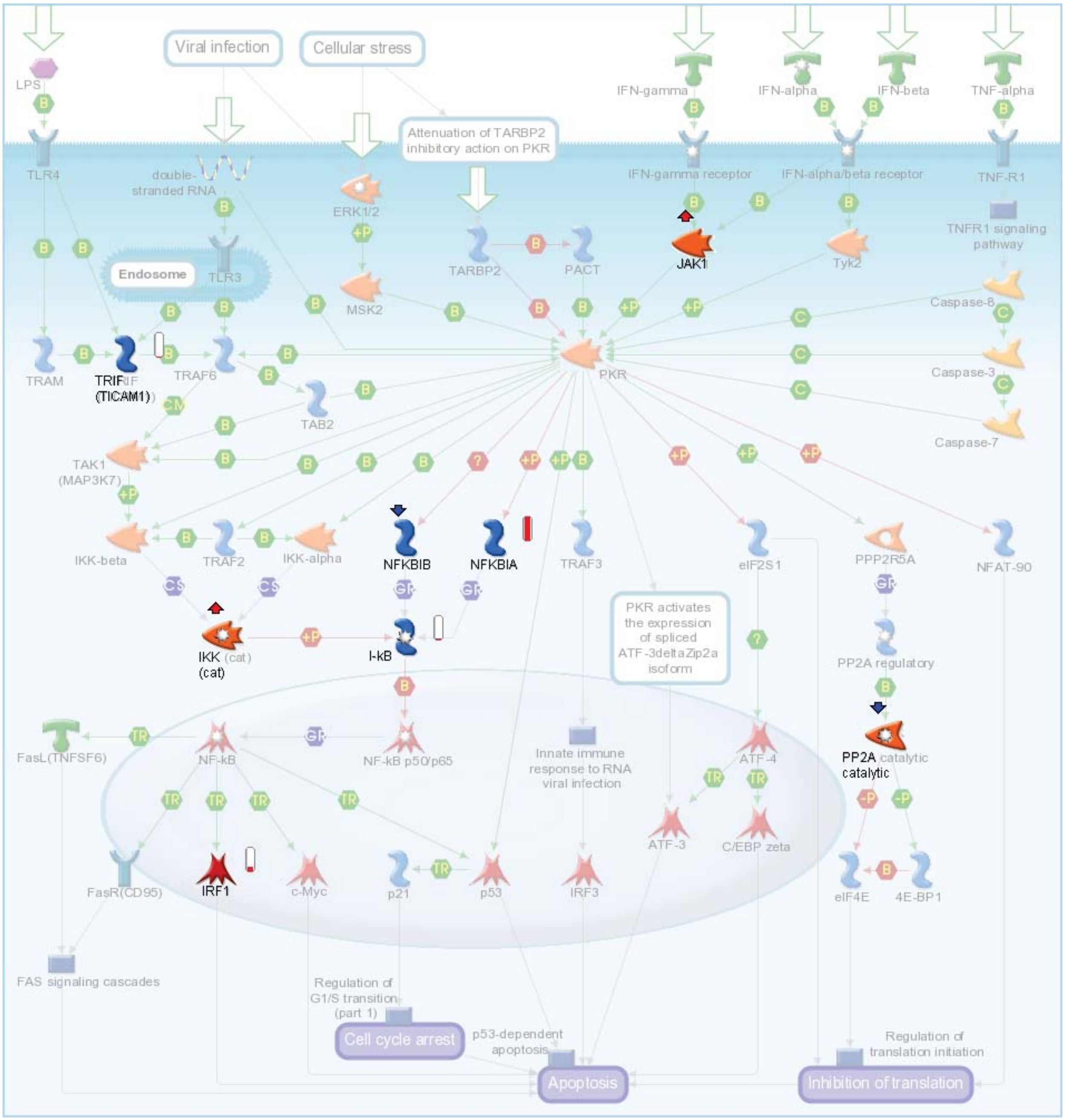

Abstract:

Activation of **double-stranded RNA**-dependent **PKR** by viral infections, extracellular stress signals and/or proinflammatory stimuli, including **LPS**, **TNF-alpha**, **IFN-gamma**, **IFN-alpha** and **IFN-beta**, results in triggering of transcriptional and translational control pathways, which regulate cell apoptosis.

Details:

The interferon-induced **double-stranded RNA**-dependent protein kinase **PKR** is a member of a small family of serine-threonine kinases that are activated by extracellular stresses, mainly viral infections (see: viral process). **PKR** mediates apoptotic process induced by many different stimuli [1], [2]. The classical activator of **PKR** is **double-stranded RNA** (dsRNA), which directly binds **PKR** and triggers **PKR** kinase activity [3].

PKR is also activated by both **TNF-alpha** and **IFN-gamma**, which act synergistically during a variety of biological responses to induce protein synthesis inhibition (see: negative regulation of translation) [4] and activation of **NF-kB** signaling with subsequent expression of genes involved in cell apoptotic process [5], [6]. **TNF-alpha** probably activates **PKR** via **TNF-R1**-induced caspase signaling (**Caspase-8**, **Caspase-3** and/or **Caspase-7**) [7], [8], [9]. **IFN-gamma** induces activation of **PKR** via **IFN-gamma receptor**/ **JAK1** pathway [10], [11]. In addition, **IFN-alpha** and **IFN-beta** are able to activate **PKR** via **IFN-alpha/beta receptor**/ **JAK1**/ **Tyk2** signaling [11].

PKR is also implicated in toll-like receptor signaling pathway, which plays an important role in pathogen recognition. **PKR** is engaged in dsRNA-activated **TLR3**/ **TRIF (TICAM1)**/ **TRAF6**/ **TAK1(MAP3K7)**/ **TAB2** signaling [12], [13], [14], [15]. **LPS**-induced **TLR4** can activate **PKR** via **TRAM**/ **TRIF (TICAM1)** signaling during apoptotic signaling pathway [16], [17], [18].

A wide range of different cellular stresses can activate **PKR** through **PACT**, independently of dsRNA or other molecules [19], [20], [21]. **TARBP2**, which inhibits both **PACT** and **PKR**, is repressed by stress stimuli [22]. Another pathway of **PKR** activation is **ERK1/2**/ **MSK2** signaling [23]. Mechanism of **PKR** activation includes its homodimerisation and autophosphorylation [24], [25].

The **PKR**-induced apoptotic process involves transcriptional and translational control pathways. **NF-kB** is a downstream mediator of **PKR**-induced apoptotic process [26]. **PKR** mediates the activation of **NF-kB p50/p65**/ **NF-kB** signaling via degradation of both **NFKBIA** and/or **NFKBIB** members of **I-kB** protein family [6], [13], [27], [28], [29], [30], or by activation of **IKK-alpha** and **IKK-beta** subunits of the **IKK (cat)** complex [12], [31], [32]. **PKR** is able to interact with **TRAF2**, which is suggested to activate downstream signal towards the **NF-kB** via **IKK-alpha** and **IKK-beta** [33], [34]. **NF-kB** target genes, which are upregulated and induce cell death upon **PKR** expression, are **FasL(TNFSF6)** , **FasR(CD95)**, **c-Myc**, **IRF1** and **p53** [1], [15], [16], [35], [36], [37], [38], [39], [40]. **PKR** also enhances **p53** transcriptional function by **p53** phosphorylation [41]. In turn, **p53** activates **p21** expression and consequent cell cycle arrest during **p53**-induced apoptotic process [42], [43]. **TRAF3** associates with **PKR** in the **IRF3** signaling pathway, which also contributes to apoptotic process [44], [45], [46].

PKR inhibits translational initiation through the phosphorylation of **eIF2S1**, thereby inhibiting protein synthesis and inducing apoptosis [4], [47], [48], [49]. **eIF2S1** activates the expression of **ATF-4** and its downstream targets **C/EBP zeta** and, probably, **ATF-3** leading to apoptotic process [21], [50], [51], [52]. Activation of **PKR** leads to the expression of the spliced ATF-3deltaZip2a form of **ATF-3**, which sensitizes cells to apoptosis [52], [53]. An alternative translational control pathway regulated by **PKR** is the **PPP2R5A**/ **PP2A regulatory**/ **PP2A catalytic** signaling, which decreases the activity of **eIF4E** [54] and activates **4E-BP1** [9], [55]. Additional pro-apoptotic **PKR**-dependent pathway that leads to inhibition of translation is phosphorylation of **NFAT-90** [56].

Maps and Descriptions [3 of 10]

Name	Input Objects ▲ p-value	Key Hubs p-value	Union Objects p-value
Immune response_CD40 signaling	2.153E-4	0.004851	5.329E-6

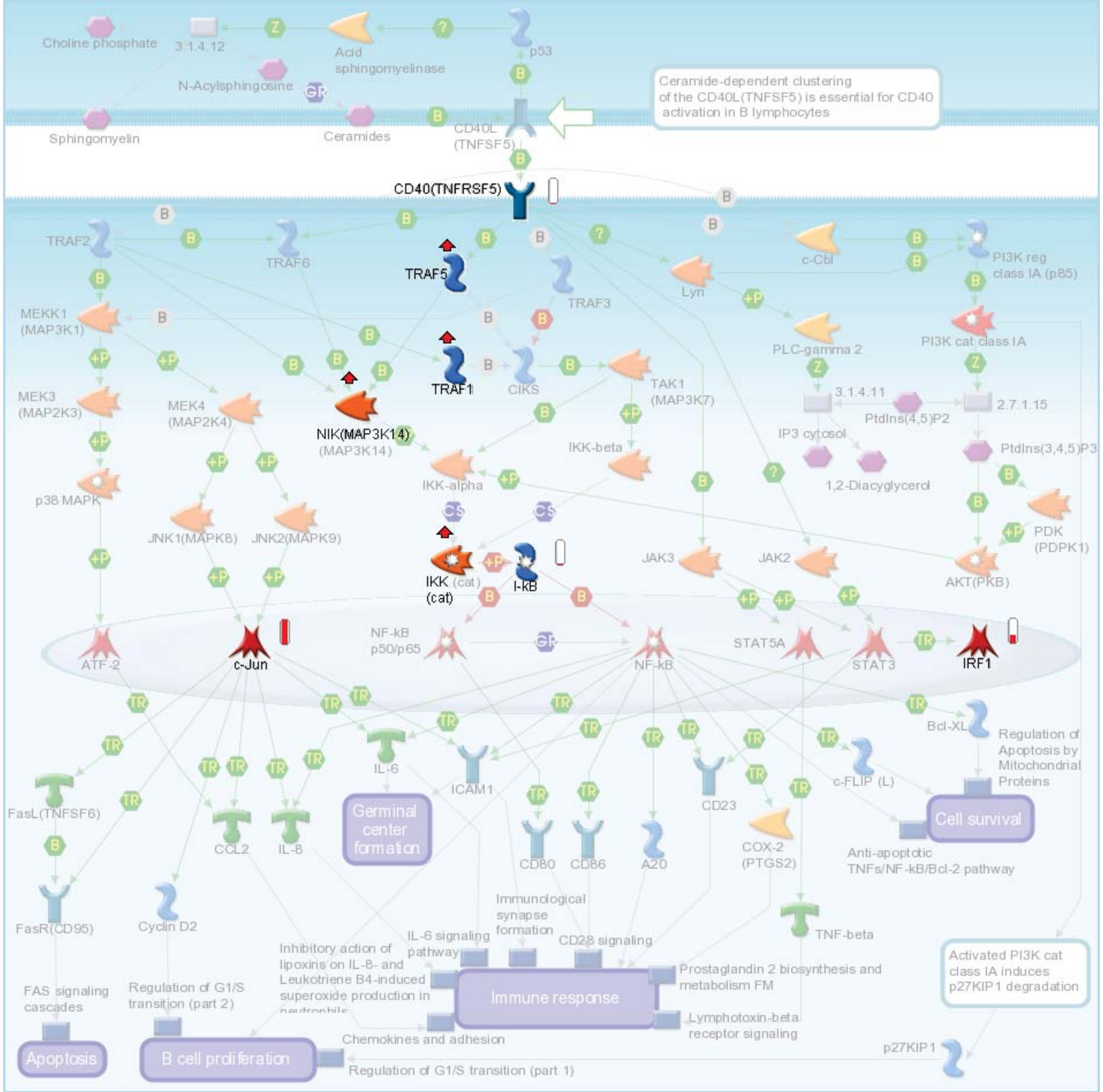

Description

CD40 signaling

CD40 molecule, TNF receptor superfamily member 5(**CD40(TNFRSF5)**) is a member of the tumor necrosis factor receptor (TNFR) superfamily that was initially described to provide activation signals in antigen-presenting cells, such as B-lymphocytes, macrophages, and dendritic cells. It is now recognized that **CD40(TNFRSF5)** is expressed in cells outside of the immune system and may play a role in some aspects of the inflammatory response in nonlymphoid cells [1], [2].

CD40(TNFRSF5) signaling is activated by **CD40(TNFRSF5)** binding to its cognate ligand CD40 ligand (**CD40L(TNFSF5)**). Receptor clustering is a key event in the initiation of signaling. Clustering of a **CD40L(TNFSF5)** is a prerequisite for clustering of the cognate receptor. Clustering of the **CD40L(TNFSF5)** is mediated by an association **CD40L(TNFSF5)** with Tumor protein p53 (**p53**). The latter induces translocation of Sphingomyelin phosphodiesterase 1, acid lysosomal (**Acid sphingomyelinase**) to the cell membrane, its activation and a formation of **Ceramides**. **Ceramides** serve to trap and cluster **CD40L(TNFSF5)** [3].

Activated **CD40(TNFRSF5)** induces activation of Nuclear factor of kappa light polypeptide gene enhancers (**NF-kB**) via association with TNF receptor-associated factors 1, 2, 3, 5, and 6 (**TRAF1**, **TRAF2**, **TRAF3**, **TRAF5** and **TRAF6**) [4], [5], [6], [7], [8]. **CD40(TNFRSF5)** binding to **TRAF2**, **TRAF5** and **TRAF6** promotes Mitogen-activated protein kinase kinase kinase 14 (**NIK(MAP3K14)**) activation [5]. **CD40(TNFRSF5)** binding to **TRAF1**, **TRAF3** and **TRAF5** induces TRAF3 interacting protein 2 (**CIKS**)-dependent Mitogen-activated protein kinase kinase kinase 7 (**TAK1(MAP3K7)**) activation [9]. **NIK(MAP3K14)** and **TAK1(MAP3K7)** phosphorylate **IKK (cat)** subunits leading to **NF-kB** activation. **NF-kB** induces **CD40(TNFRSF5)**-dependent expression of Prostaglandin-endoperoxide synthase 2 (**COX-2(PTGS2)**) and Prostaglandin E2 synthesis [10], [11], [12]. Also, **NF-kB** activates production of Fc fragment of IgE, low affinity II, receptor for (**CD23**), Tumor necrosis factor, alpha-induced protein 3 (**Zinc finger protein A20**), CD80 and CD86 molecules (**CD80**, **CD86**), Intercellular adhesion molecule 1 (**ICAM1**), Interleukines 6 and 8 (**IL-6**, **IL-8**) [1], [5], [13]. **TRAF3** could act as pro-apoptotic agent by inhibiting **CIKS** and preventing **NF-kB** activation [9].

CD40(TNFRSF5) binding to **TRAF2** and **TRAF3** leads to Mitogen-activated protein kinase kinase kinase 1 (**MEKK1(MAP3K1)**) activation and subsequent Mitogen-activated protein kinase 14 (**p38 MAPK**), Mitogen-activated protein kinases 8 and 9 (**JNK1(MAPK8)** and **JNK2(MAPK9)**) via Mitogen-activated protein kinase kinase 3 and 4 (**MEK3(MAP2K3)** and **MEK4(MAP2K4)**) [14], [15], [16]. **p38 MAPK**, **JNK1(MAPK8)** and **JNK2(MAPK9)** phosphorylate Activating transcription factor 2 (**ATF-2**) and Jun oncogene (**c-Jun**), respectively, leading to production of Chemokine (C-C motif) ligand 2 (**CCL2**), **IL-6**, **IL-8**, **Cyclin D2**, **ICAM1** and stimulating spleen germinal centers formation, B cell proliferation and immune response [2], [17]. **JNK1(MAPK8)** and **JNK2(MAPK9)** activation by **CD40(TNFRSF5)** also leads to enhanced Fas ligand (**FasL(TNFSF6)**) and Fas (**FasR(CD95)**) expression and induction apoptosis [16], [18].

CD40(TNFRSF5) could activate Janus kinases 2 and 3 (**JAK2** and **JAK3**) leading to Signal transducers and activators of transcription 3 and 5A (**STAT3** and **STAT5A**) activation. This pathway mediates **CD40(TNFRSF5)**-induced expression of Interferon regulatory factor 1 (**IRF1**), Lymphotoxin alpha (**TNF-beta**), **CD23** and **ICAM1** [19], [20].

CD40(TNFRSF5) association with **TRAF6** and Cas-Br-M (murine) ecotropic retroviral transforming sequence (**c-Cbl**) and v-yes-1 Yamaguchi sarcoma viral related oncogene homolog (**Lyn**) stimulation leads to Phosphoinositide-3-kinase, regulatory subunits (**PI3K reg class IA (p85)**) activation [21], [22], [23]. Activation of Phosphatidylinositol 3-kinase, catalytic (**PI3K cat class IA**) induces Cyclin-dependent kinase inhibitor 1B (**p27KIP1**) degradation and stimulates B cell proliferation [24]. Also, **PI3K cat class IA** promotes activation of v-akt murine thymoma viral oncogene homologs (**AKT(PKB)**). The latter phosphorylates Conserved helix-loop-helix ubiquitous kinase (**IKK-alpha**) inducing **NF-kB** activation. **NF-kB** activates transcription of BCL2-like 1 (**Bcl-XL**) and CASP8 and FADD-like apoptosis regulator (**c-FLIP**

(L)) mediating anti-apoptotic effect of **CD40(TNFRSF5)** engagement [24], [25], [26].

In addition, **CD40(TNFRSF5)** via **Lyn** activation induces phosphorylation of Phospholipase C, gamma 2 (**PLC-gamma 2**) and stimulates **IP3 cytosol** and **1,2-Diacyglycerol** production [21].

Maps and Descriptions [4 of 10]

Name	Input Objects ▲ p-value	Key Hubs p-value	Union Objects p-value
SLE genetic marker-specific pathways in antigen-presenting cells (APC)	5.76E-4	0.001901	4.378E-6

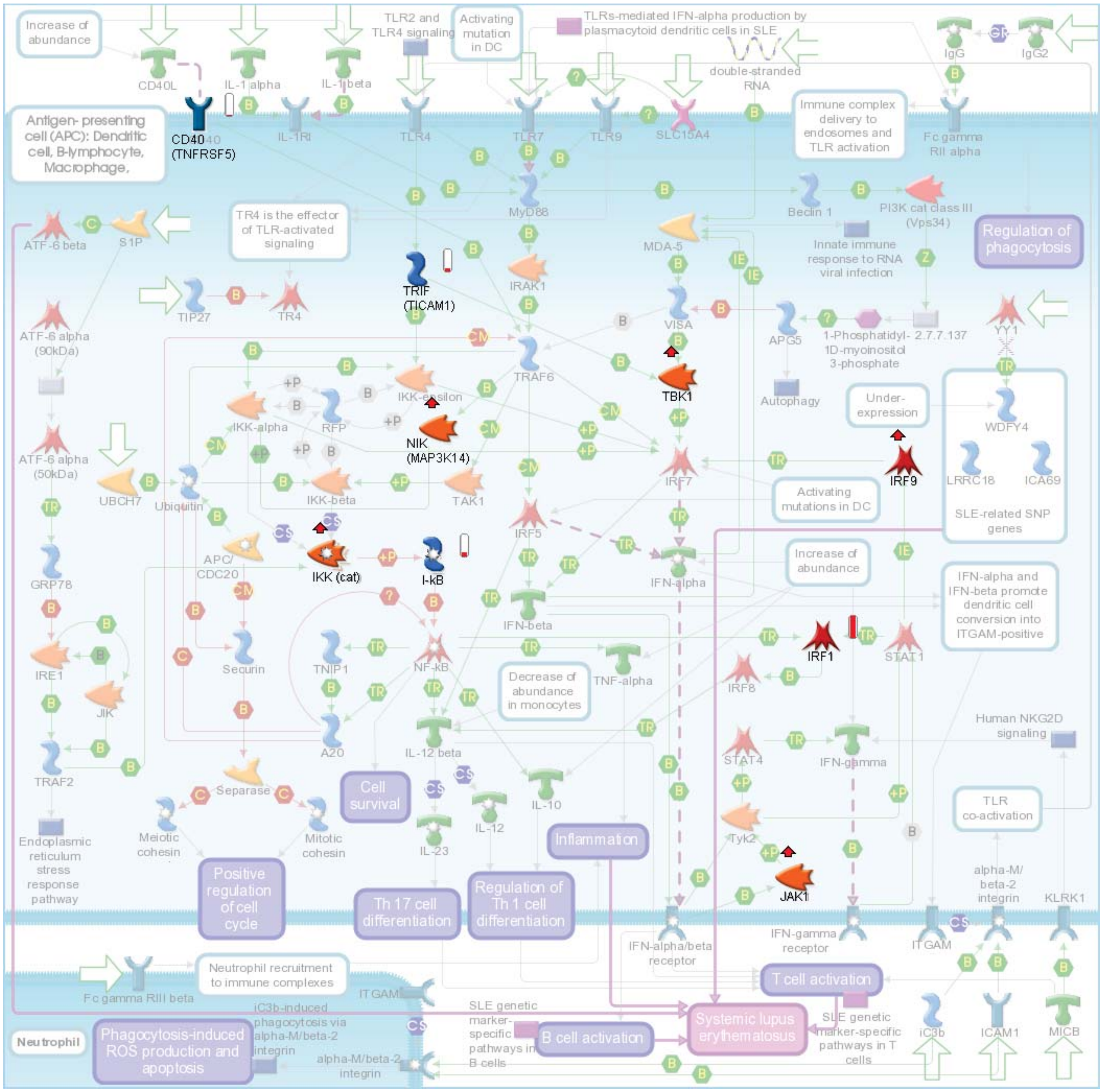

Abstract:

SLE risk gene SNPs, associated with antigen-presenting cell functioning, include **IRAK1**, **IRF5**, **IRF7**, **IRF8**, **TIP27**, **RFP**, **TNIP1**, **A20**, **UBCH7**, **Securin**, **Tyk2**, **STAT4**, **IL-12 beta**, **IL-10**, **MDA-5**, **SLC15A4**, **APG5**, **ITGAM**, **Fc gamma RII alph**a, **Fc gamma RIII beta**, **PXK**, **ATF-6 beta**, **JIK**, **MICB** and probably **WDFY4**, **ICA69**, **LRRC18**. They are implicated in several signaling cascades, among which are **TLR4** , **TLR7**, **TLR9**, **CD40L(TNFSF5)**, **IL-1 alpha**, **IL-1 beta, IFN-alpha**, **IFN-beta** and **MICB**-activated pathways.

Details:

Systemic lupus erythematosus (SLE) is a chronic systemic autoimmune heterogeneous disease, characterized by immunological dysfunction of multiple components of both the innate and adaptive immune systems. Alterations of antigen-presenting cell (APC) homeostasis, namely dendritic cells, monocytes, macrophages and B-lymphocytes, are directly implicated in SLE [1], [2], [3].

Researches indicate that SLE has a significant genetic component. Inherited genetic variations include common and rare single nucleotide polymorphisms (SNPs) [4], [5].

Multiple risk gene SNPs were designated for SLE, which are involved in different signaling pathways in immune cells. Risk genes, associated with antigen-presenting cell functioning, include **IRAK1**, **IRF5**, **IRF7**, **IRF8**, **TIP27**, **RFP**, **TNIP1**, **A20**, **UBCH7**, **Securin**, **Tyk2**, **STAT4**, **IL-12 beta**, **IL-10**, **MDA-5**, **SLC15A4**, **APG5**, **ITGAM**, **Fc gamma RII alpha**, **Fc gamma RIII beta**, **ATF-6 beta**, **JIK**, **MICB** and probably **WDFY4**, **ICA69**, **LRRC18** [4], [5], [6], [7], [8], [9], [10], [11].

In SLE, loss of self-tolerance and the resulting circulating autoantibodies prompt the formation of **IgG**-containing immune complexes, that are endocytosed by plasmacytoid dendritic cells (pDC) via **Fc gamma RII alpha** receptor and further activate **TLR7** and **TLR9** [12], [13], [14], [15].

SLC15A4 also promotes **TLR7** and **TLR9**-activated signaling in dendritic cells [16], [17].

TLR4, **TLR7** and **TLR9** induce **MyD88**/ **IRAK1**/ **TRAF6**-mediated signaling pathway, **TRIF (TICAM1)**/ **IKK-epsilon** signaling pathway and **TRIF (TICAM1)**/ **TBK1**-mediated signaling pathway, leading to activation of **IRF5** and **IRF7**, which induce production of various pro-inflammatory mediators including **IL-12 beta**, **IFN-alpha** and **IFN-beta** [18], [19], [20], [21]. In SLE transcription levels of **TLR7** are increased due to G-allele of rs3853839G/C single nucleotide polymorphism in pDC [22]. Moreover, activating polymorphisms of **IRF5** [23], [24] and **IRF7** [25], [26] lead to increased levels of these proteins in pDC in SLE. These activating mutations result in elevation of **IFN-alpha** production and plasma/ serum levels in SLE and enhanced **IFN-alpha** signaling [27].

Furthermore, **TLR4**, **TLR7** and **TLR9** activate **MyD88**/ **IRAK1**/ **TRAF6**/ **IKK-alpha**/ **IKK (cat)**/ **I-kB** signaling and **MyD88**/ **IRAK1**/ **TRAF6**/ **TAK1(MAP3K7)**/ **IKK-beta**/ **IKK (cat)**/ **I-kB** signaling, resulting in **NF-kB** activation and **IL-12 beta**, **IL-10** and **TNF-alpha** production. SNP genes **RFP**, **TNIP1**, **A20**, **UBCH7** are implicated in regulation of **TLR4**, **TLR7** and **TLR9**-activated **NF-kB** signaling [5], [18], [19], [20], [28], [29], [21]. **TNF-alpha** [27], [30], [31] and **IL-10** [30], [32] plasma/ serum levels are elevated in SLE patients. Conversely, protein production of **IL-12 beta** is decreased in activated monocytes of SLE patients [32].

TLR4, **TLR7** and **TLR9** effect **TR4**, which is regulated by **TIP27** [20], [33], [34], [35].

TLR7 via **MyD88** promotes **Beclin 1**/ **PI3K cat class III (Vps34)**)/ **1-Phosphatidyl-1D-myoinositol 3-phosphate** signaling, leading to activation of **APG5**, which is a regulator of dendritic cell autophagy required for **IFN-alpha** production [36].

CD40L(TNFSF5) via **CD40(TNFRSF5)**/ **TRAF6**/ **NIK(MAP3K14)**/ **IKK-alpha**/ **IKK (cat)**/ **I-kB** cascade [37], [38] and **IL-1 alpha** and/or **IL-1 beta** via **IL-1RI**/ **MyD88**/ **IRAK1**/ **TRAF6**/ **TAK1(MAP3K7)**/ **IKK-beta**/ **IKK (cat)**/ **I-kB** cascade [39], [40], [41], [42] also induce activation of **NF-kB** signaling, regulated by **RFP**, **TNIP1**, **A20**, **UBCH7**. **CD40L(TNFSF5)** level has been found to be elevated in T and B cells of some lupus patients [43]. **IL-1 beta** protein level is elevated in serum of SLE patients [30].

double-stranded RNA-activated **MDA-5** induces **VISA**/ **TBK1**/ **IRF7** signal, leading to activation of **IFN-alpha** and **IFN-beta** expression [44], [45].

IFN-alpha and IFN-beta via IFN-alpha/beta receptor trigger Tyk2/ STAT4 signaling pathway, leading to IFN-gamma production. IFN-gamma activates IFN-gamma receptor/ STAT1/ IRF1/ IRF8 signal, which may contribute to IL-12 beta expression [3], [46], [47]. Protein levels of IFN-gamma are increased in serum of SLE patients [30].

IFN-alpha and IFN-beta can increase the number of ITGAM-positive dendritic cells [48]. In dendritic cells ITGAM/ alpha-M/beta-2 integrin act as a co-activator of Toll-like receptor signaling [49].

In neutrophils ITGAM/ alpha-M/beta-2 integrin promote cell migration via binding to ICAM1, and binding to iC3b implicates phagocytosis-induced reactive oxygen species (ROS) production (see: regulation of reactive oxygen species metabolic process) and apoptotic process [50], [51].

Fc gamma RIII beta is expressed by neutrophils and promotes neutrophil recruitment to immune complexes in immune complex-mediated inflammation [52], [53].

The regulator of chromosomal stability Securin is degraded by APC/CDC20 complex and participates in the cleavage of the sister chromatid cohesion complex, thereby regulating cell cycle progression [54].

S1P-activated ATF-6 beta acts together with ATF-6 alpha (90kDa) as a regulator of endoplasmic reticulum (ER) cellular stress and unfolded-protein response-induced ATF-6 alpha (50kDa)/ GRP78/ IRE1/ TRAF2/ IKK (cat)/ I-kB/ NF-kB signaling, which regulates cell survival (see: negative regulation of apoptotic process). JIK is implicated in ER cellular stress-induced signaling [55], [56], [57].

SLE-related SNP gene WDFY4 is regulated by YY1 and has reduced expression level in peripheral blood mononuclear cells of SLE patients due to lower transcriptional activity of YY1 towards SLE-associated WDFY4 A-allele of rs877819A/G single nucleotide polymorphism [10].

MICB can activate KLRK1 (NKG2D) signaling pathway in dendritic cells, which can result in IFN-gamma production [58], [59], [60].

Thus, autoantibody-induced Fc gamma RII alpha-mediated activation of TLR7/ TLR9 signaling and activating polymorphisms of IRF5 and IRF7 result in elevated production of IFN-alpha. IFN-alpha, TNF-alpha and IL-10 are key regulators of inflammation, B cell, T cell and APC function and autoimmunity in SLE [2], [3], [61].

Maps and Descriptions [5 of 10]

Name	Input Objects ▲ p-value	Key Hubs p-value	Union Objects p-value
Immune response_IL-9 signaling pathway	5.84E-4	5.33E-4	8.799E-7

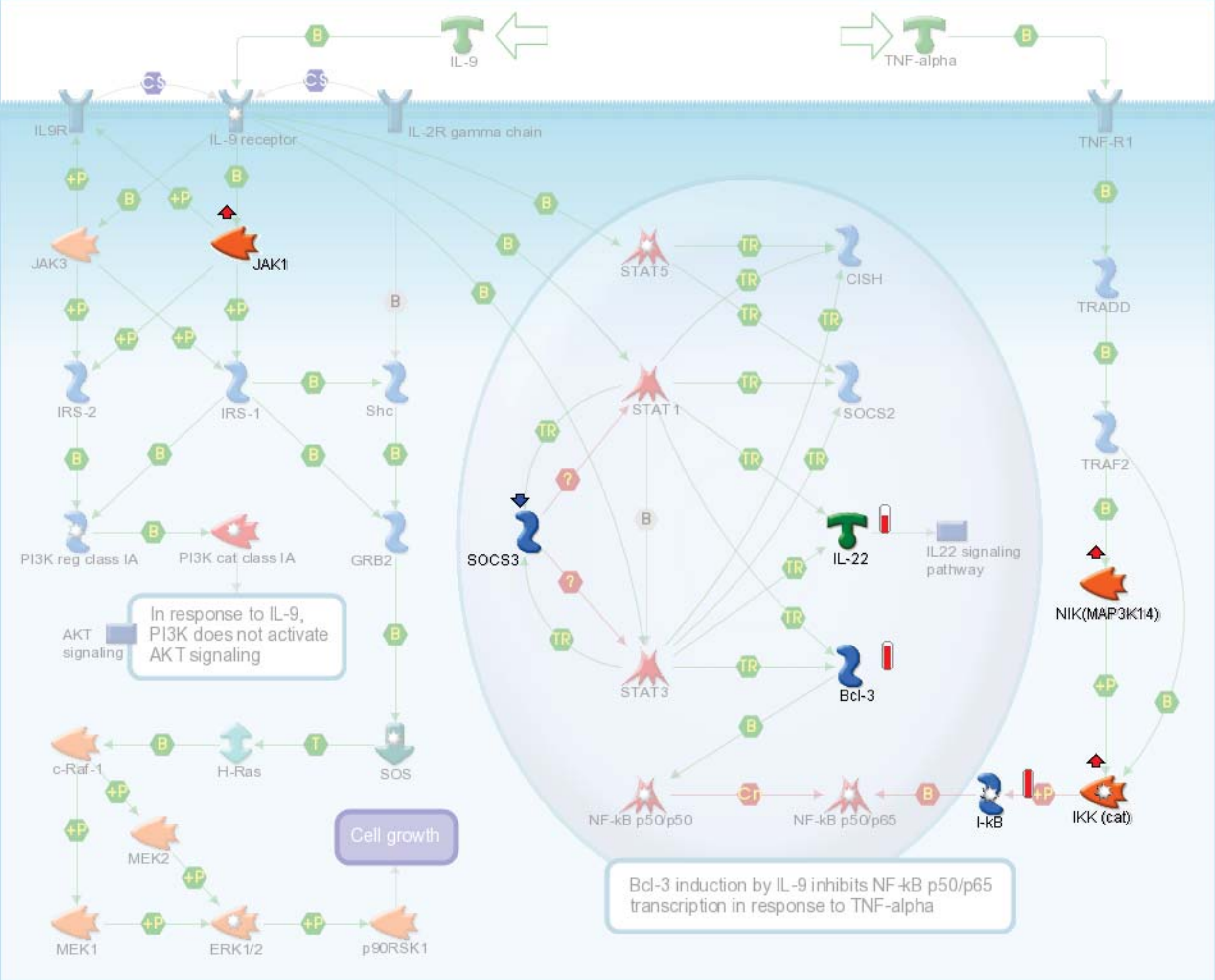

Description

IL-9 signaling pathway

Interleukin-9 (**IL-9**) is a multifunctional cytokine secreted by T helper 2 (Th2) lymphocytes. **IL-9** exerts various effects on a variety of cell types associated with allergic inflammation. **IL-9** stimulates the growth and proliferation of T cells, enhances the production of IgE from B cells, and promotes the proliferation and differentiation of mast cells and hematopoietic progenitors [1], [2]. Besides the role of **IL-9** during immune responses, its growth factor and antiapoptotic activities on multiple transformed cells suggest its potential role in tumorigenesis [3].

IL-9 binds to the heterodimeric receptor (**IL-9 receptor**) comprising a specific chain (**IL9R**) and gamma chain (**IL-2R gamma chain**). **IL-2R gamma chain** is shared by the receptors for Interleukins IL-2, IL-4, IL-7, IL-15 and IL-21.

IL-9 receptor ligation results in auto and/or trans-phosphorylation of Janus kinases 1 and 3 (**JAK1** and **JAK3**), phosphorylation of the receptor, and activation of the pathways involved in **IL-9** signaling. These pathways include Signal transducer and activator of transcription 1, 3 and 5 (**STAT1**, **STAT3** and **STAT5**), Insulin receptor substrate 1 and 2 (**IRS-1** and **IRS-2**)/ Phosphoinositide-3-kinase (**PI3K reg class IA**/ **PI3K cat class IA**) and Extracellular signal regulated kinases 1 and 2 (**ERK1/2**) [3].

In response to **IL-9**, transcriptional activities of **STAT1** and **STAT3** are more related to differentiation processes, whereas **STAT5,** or both **STAT1** and **STAT3,** are more related to the protection against apoptosis and cell proliferation [4].

STAT1 and **STAT3**, activated by **IL-9**, up-regulate the transcription of Interleukin-22 (**IL-22**), an inducible cytokine belonging to the IL-10 family that is involved in the generation of inflammatory and allergic responses [5].

IL-9 induces the expression of three cytokine signal inhibitors, Cytokine inducible SH2-containing protein (CISH), Suppressors of cytokine signaling 2 and 3 (**SOCS2** and **SOCS3**). However, only **SOCS3** exerts a negative effect on **IL-9** activities, such as **STAT3** activation and protection against apoptosis [6], [7].

IL-9 also induces B-cell CLL/lymphoma 3 (**Bcl-3**) transcription by **STAT1** and **STAT3** in T cells and mast cells. **Bcl-3** expression is followed by an increase in the DNA binding of Nuclear factor-kappa B p50 homodimers (**NF-kB p50/p50**) that can efficiently compete with NF-kB p65/p50 heterodimers (**NF-kB p50/p65**) for the sites of NF-kB DNA binding [8]. Tumor necrosis factor alpha (**TNF-alpha**), a proinflammatory cytokine, induces **NF-kB p50/p65** transcriptional activity via Tumor necrosis factor receptor superfamily member 1A (**TNF-R1**)/ TNFRSF1A-associated via death domain (**TRADD**)/ TNF receptor-associated factor 2 (**TRAF2**)/ Mitogen-activated protein kinase kinase kinase 14 (**NIK(MAP3K14**)/ NF-kB inhibitor kinase complex (**IKK (cat)**)/ NF-kB inhibitor (**I-kB**) signaling pathway, leading to the expression of **NF-kB p50/p65** target genes [9], [10]. **IL-9** via **Bcl-3** expression specifically down-regulates a particular set of genes induced by **NF-kB p50/p65** in response to **TNF-alpha** [8].

IL-9 can induce the phosphorylation of ectopically expressed **IRS-1** in T cells and of endogenous **IRS-2** in other hematopoietic cells. After tyrosine phosphorylation, **IRS-1** and **IRS-2** interact with SH2-containing signaling proteins, such as **PI3K reg class IA**, SHC transforming protein 1 (**Shc**) and Growth factor receptor-bound protein 2 (**GRB2**) [3].

Positioned downstream of the **PI3K reg class IA**/ **PI3K cat class IA** signaling, the v-Akt murine thymoma viral oncogene homolog (AKT(PKB)) does not seem to be the main effector of **IL-9**-activated **IRS-1** and **IRS-2** [11], [12].

A pathway that occurs downstream of **Shc**/ **GRB2** signaling involves stimulation of Son of sevenless homologs (**SOS**)/ v-Ha-ras Harvey rat sarcoma viral oncogene homolog (**H-Ras**)/ v-Raf-1 murine leukemia viral oncogene homolog 1 (**c-Raf-1**)/ Mitogen-activated protein kinase kinase 1 and 2 (**MEK1** and **MEK2**)/ **ERK1/2**/ Ribosomal protein S6 kinase 90kDa polypeptide 1 (**p90RSK1**). This pathway leads to growth stimulation of hematopoietic cell lines [3], [13].

Maps and Descriptions [6 of 10]

Name	Input Objects ▲ p-value	Key Hubs p-value	Union Objects p-value
Immune response_TNF-R2 signaling pathways	0.001128	0.001254	4.272E-6

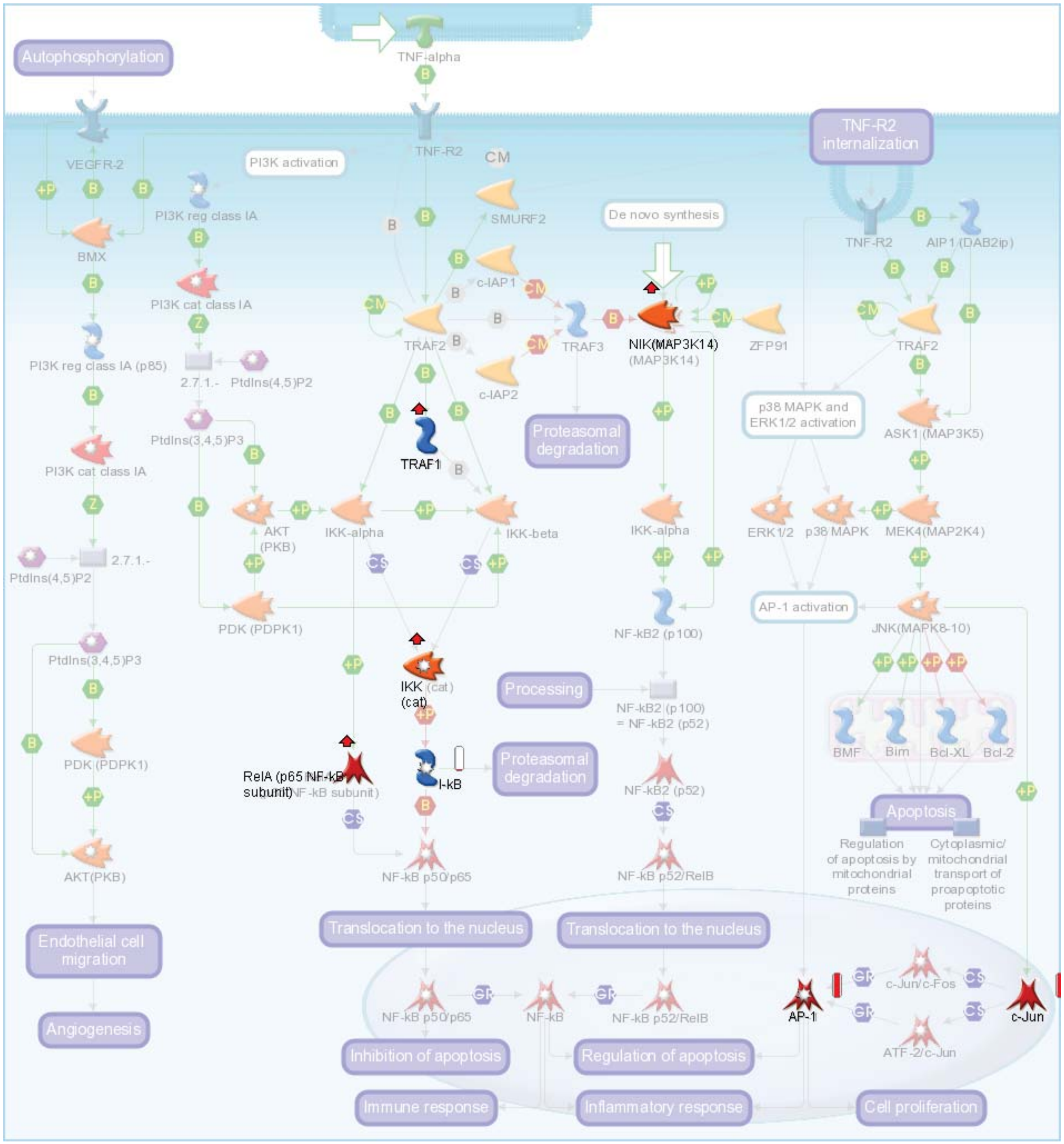

Abstract:

TNF-R2 is activated only by membrane-bound **TNF-alpha**. The ability of **TNF-R2** to induce different signaling pathways depends on its cellular localization that is mainly regulated by **TRAF2** binding to distinct **TNF-R2** cytoplasmic domens. **TNF-R2** on plasma membrane activates a **BMX**-mediated pathway (that is required for endothelial cell migration during inflammatory angiogenesis), and **TRAF2**-dependent canonical and non-canonical pathways of **NF-kB** activation (that are involved in regulation of apoptosis, inflammatory response and immune response). **TNF-R2** internalization results in association with **AIP1 (DAB2ip)** and activation of **JNK(MAPK8-10)**, that leads to cell apoptosis and/or **AP-1**-mediated cell proliferation and inflammatory response.

Details:

TNF-R2, the second receptor known for **TNF-alpha**, is activated only by membrane-bound **TNF-alpha**. Expression of **TNF-R2** is restricted to endothelial cells, human mesenchymal stem cells, T cells, myocytes, thymocytes, oligodendrocytes and astrocytes. Although **TNF-alpha** signaling pathways via its first receptor, tumor necrosis factor receptor superfamily, member 1A (TNF-R1), are widely characterized, the **TNF-R2** signaling pathways are poorly understood and several **TNF-R2** functions still remain unclear [1], [2].

TNF-alpha binding to **TNF-R2** forces its trimerization which leads to its direct interaction with **TRAF2** and association with **TRAF1**, **TRAF3**, **c-IAP1** and **c-IAP2** through their interactions with **TRAF2** [1], [2], [3], [4], [5], [6], [7]. Thus, **TRAF2** acts as a key mediator in the **TNF-R2** signaling which leads to transcriptional activation of genes related to cell proliferation, regulation of apoptotic process, inflammatory response and immune response [1], [2], [3], [8], [9], [10], [11]. **TRAF2** protein autoubiquitination (namely, protein K63-linked ubiquitination) is critically required for its activity [12], [13], [14], [15].

TNF-alpha-induced **TNF-R2** signaling pathways lead to activation of both **NF-kB** [2], [3], [6], [9], [16], [17], [18], [19], [20], [21] and **JNK(MAPK8-10)** [2], [3], [8], [21], [22], [23], [24], [25], [26]. Notably, **TNF-R2** ability to activate either **NF-kB** or **JNK(MAPK8-10)** signaling depends on its cellular localization that is regulated by **TRAF2** interactions with distinct **TNF-R2** cytoplasmic domens. Binding of **TRAF2** (or **BMX**) to **TNF-R2** via a so-called "domen I" maintains the receptor on the plasma membrane that leads to **NF-kB** activation (or **BMX**-mediated endothelial cell migration), whereas **TRAF2** interactions with so-called "domain II" and "domain III" of **TNF-R2** are required for the receptor internalization and its ability to activate **JNK(MAPK8-10)** [21]. In addition, a **TRAF2** binding protein **SMURF2** ubiquitinates **TNF-R2** to trigger **TNF-R2** relocalization and **TNF-R2**-induced **JNK(MAPK8-10)** activation [26].

The **TNF-alpha**/ **TNF-R2**-induced **BMX** signaling seems to be independent of **TRAF2** [2], [27]. Stimulation of endothelial cells with **TNF-alpha** induces **TNF-R2** binding to **BMX** that leads to **BMX** association with **VEGFR-2** resulting in **VEGFR-2** activation by protein autophosphorylation, typically without ligand binding. In turn, activated **VEGFR-2** phosphorylates **BMX**, thus stimulating **BMX** binding to **PI3K reg class IA (p85)** and activation of a **PI3K reg class IA (p85)**/ **PI3K cat class IA**/ **PtdIns(3,4,5)P3**/ **PDK (PDPK1)**/ **AKT(PKB)** signaling pathway [28], [29], [30]. This pathway leads to endothelial cell migration involved in angiogenesis [27], [28], [31], [32], [33], [34], [35], [36]

In response to **TNF-alpha**, plasma membrane **TNF-R2** also induces both the canonical [2], [3], [17], [19] and non-canonical pathways of **NF-kB** activation [2], [3], [6], [9], [16], [37], [38].

The canonical I-kappaB kinase/NF-kappaB pathway depends on activation of **IKK-alpha** and **IKK-beta**, two catalytic subunits (**IKK (cat)**) of the kinase complex that phosphorylates **I-kB** proteins for subsequent ubiquitin-dependent proteasomal degradation (proteasome-mediated ubiquitin-dependent protein catabolic process). Notably, inhibitory **I-kB** proteins retain **NF-kB** dimers (such as **NF-kB p50/p65**) in the cytoplasm. **I-kB** degradation allows liberated **NF-kB** dimers to translocate to the nucleus (NF-kappaB import into nucleus) to regulate gene expression [2], [39], [40], [41], [42].

TNF-alpha can stimulate the recruitment of **IKK-alpha** and **IKK-beta** to the cell membrane signaling complex by **TRAF2** binding to **IKK-alpha** and **IKK-beta** [14], [43]. In addition, binding of **TRAF1** to **IKK-beta** can exert both inhibitory and stimulatory effects on **IKK-beta**

signaling [44]. Subsequently, phosphorylation of **IKK-alpha** and **IKK-beta**, that is critically required for their activity, is mediated via the **TNF-alpha**/ **TNF-R2**/ **PI3K reg class IA**/ **PI3K cat class IA**/ **PtdIns(3,4,5)P3**/ **PDK (PDPK1)**/ **AKT(PKB)** signaling pathway [29], [30], [45], [46]: **AKT(PKB)** phosphorylates **IKK-alpha** [47], [48], while **PDK (PDPK1)** [49] and **IKK-alpha** [50] can phosphorylate **IKK-beta**. Then, catalytically active **IKK (cat)** subunits phosphorylate **I-kB** proteins to activate **NF-kB p50/p65** translocation to the nucleus resulting in expression of genes that promote anti-apoptotic responses (negative regulation of apoptotic process) [2], [17], [39], [40], [42], [45], [51]. In addition, **IKK-alpha** can directly phosphorylate **RelA (p65 NF-kB subunit)** to stimulate its transcriptional activity [17], [52], [53].

The non-canonical pathway of **NF-kB** activation depends on the protein processing of **NF-kB2 (p100)** to yield **NF-kB2 (p52)**, an active subunit of the **NF-kB p52/RelB** complex. This pathway is triggered by **NIK(MAP3K14)** [2], [37], [38], [39], [40].

In unstimulated cells, newly synthesized **NIK(MAP3K14)** is rapidly ubiquitinated (namely, by protein K48-linked ubiquitination) in a **TRAF3**/ **TRAF2**/ **c-IAP1**/ **c-IAP2**-dependent manner and degraded in proteasome. In cells stimulated with **TNF-alpha**, the **TNF-R2**/ **TRAF2**/ **c-IAP1**/ **c-IAP2** signaling complex induces **TRAF3** protein K48-linked ubiquitination and targeting for proteasomal degradation. As a result, in the absence of sufficient **TRAF3**, de novo synthesized **NIK(MAP3K14)** is accumulated and subsequently activated via the protein autophosphorylation and **ZFP91**-dependent protein K63-linked ubiquitination [37], [38], [54], [55], [56].

Once activated, **NIK(MAP3K14)** phosphorylates and activates **IKK-alpha** [38], [55], [57]. Then, both **NIK(MAP3K14)** and **IKK-alpha** phosphorylate **NF-kB2 (p100)**. The protein phosphorylation triggers **NF-kB2 (p100)** protein processing to yield **NF-kB2 (p52)** [38], [58], [59], [60], [61], [62], [63]. Subsequently, the active **NF-kB p52/RelB** complex translocates to the nucleus and induces expression of target genes [37], [63], [2], [38].

Non-plasma membrane bound **TNF-R2** doesn't activate **NF-kB** signaling. Instead, it stimulates the **JNK(MAPK8-10)** cascade. As it has been mentioned above, **TNF-alpha**-induced binding of **TRAF2** to **TNF-R2** via "domain II" and "domain III" leads to the receptor internalization [21]. In addition, **TRAF2** binding to **SMURF2** triggers **TNF-R2** ubiqiutination and relocalization [26]. Inside the cell, **TNF-R2** "domain III" is responsible for **TNF-R2** association with **AIP1 (DAB2ip)** [21], a signaling molecule that specifically activates the **JNK(MAPK8-10)** signaling by recruiting **TRAF2** and **ASK1 (MAP3K5)** to each other [21], [64], [65]. **TRAF2** then binds to and activates **ASK1 (MAP3K5)** [21], [65], [66] that subsequently phosphorylates and activates **MEK4(MAP2K4)**, the main up-stream kinase of **JNK(MAPK8-10)** [3], [7], [66], [67], [68], [69], [70]. Activated **JNK(MAPK8-10)** then induces cell apoptotic process [21], [64] (e.g., by phosphorylating mitochondrial proteins, such as pro-apoptotic proteins **Bim** and **BMF**, and anti-apoptotic proteins **Bcl-XL** and **Bcl-2** [64], [69]), or can mediate cell proliferation, regulation of apoptotic process and inflammatory response (mainly by phosphorylating transcription factors of the **AP-1** family such as **c-Jun**, a subunit of **c-Jun/c-Fos** and **ATF-2/c-Jun** complexes) [1], [2], [3], [10], [67], [69], [71], [72], [73].

In several cell types, **TNF-R2**, most likely after the receptor internalization and, probably, via **TRAF2**, can stimulate **p38 MAPK** and **ERK1/2** signaling, which leads, at least in part, to **AP-1** activation [8], [25], [26], [74], [75], [76], [77], [78], [79], [80], [81], [82], [83].

Maps and Descriptions [7 of 10]

Name	Input Objects ▲ p-value	Key Hubs p-value	Union Objects p-value
Immune response_Bacterial infections in normal airways	0.001445	0.001726	7.689E-6

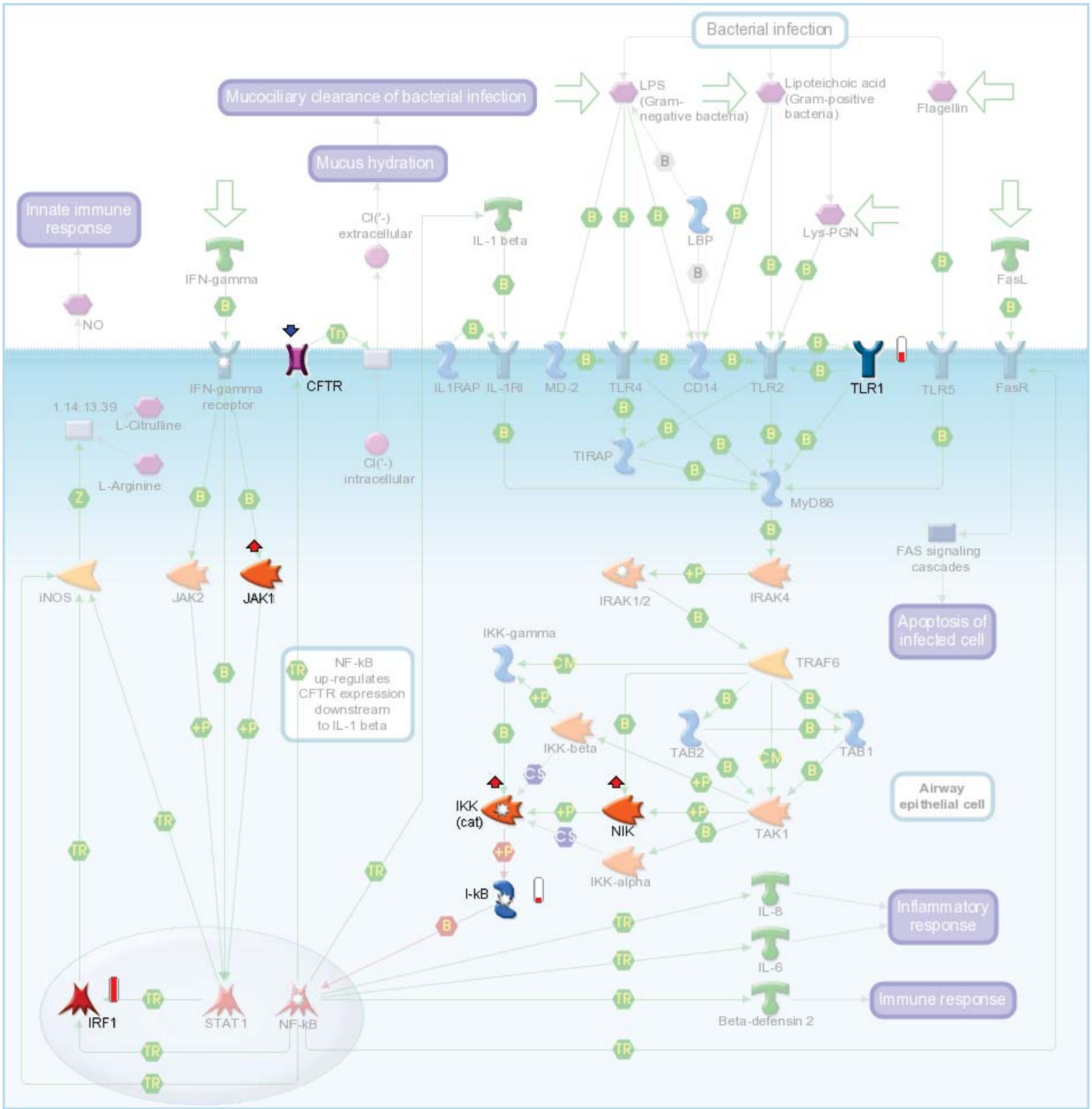

Description

Bacterial infections in normal airways

The upper airways represent a primary site for the introduction of pathogenic microorganisms from inspired air. The ciliated epithelium features several powerful mechanisms for prevention of colonization by inhaled bacteria, thus the lower respiratory tract usually remains sterile. Toll-like receptors (TLRs) play a key role in facilitating the innate immune response to bacterial antigens [1].

Toll-like receptors (TLRs) belong to a family of transmembrane proteins that can recognize and discriminate a diverse array of microbial antigens. Following their activation by specific bacterial ligands, TLRs initiate intracellular signaling cascades that culminate in the activation of transcription factors and ultimately lead to activation of pro-inflammatory gene expression (acute inflammatory response). Epithelial airway cells provide both a physical barrier to infection and an active defense mechanism against invading microoranisms [2].

TLR2 is the predominant TLR expressed on the apical cell surface, with other TLRs (**TLR1**, **TLR4** and **TLR5**) residing mainly intracellularly. However, in inflamed lung following stimulation with bacterial ligands **TLR5** and **TLR4** can be mobilized to the apical surface [2].

All TLRs, as well as **IL-1RI**, induce the canonical pathway of **NF-kB** activation which consists of **MyD88**/ **IRAK4**/ **IRAK1/2**/ **TRAF6**/ **TAB1** and **TAB2**/ **TAK1(MAP3K7)**/ **NIK(MAP3K14)**/ **IKK (cat)**/ **I-kB**/ **NF-kB** cascade [2], [3], [4]. **TLR2** and **TLR4** signaling pathways also require an additional adaptor **TIRAP (Mal)** [5], [6].

TLR signaling and **NF-kB** activation are commonly involved in the up-regulation of chemotactic molecules and cytokines (such as Interleukins **IL-1 beta**, **IL-6** and **IL-8**), production of mediators of innate immune response (such as **NO** that is synthesized by **iNOS**), enhanced expression of antimicrobial peptides (such as **Beta-defensin 2**). **IL-1 beta** signaling, in turn, regulates the levels of **CFTR** [7], [8].

Of all the TLRs, **TLR2** in conjunction with **TLR1** recognizes the broadest repertoire of ligands, such as **Lipoteichoic acid** and peptidoglycan **Lys-PGN** from Gram-positive bacteria followed by **NF-kB** activation and interleukin production [2], [9], [10]. **TLR5** is able to recognize **Flagellin** (from both Gram-positive and Gram-negative bacteria) and also to stimulate **NF-kB** signaling [1], [11], [12].

Pseudomonas aeruginosa has been shown to signal through **TLR4**/ **MD-2**/ **CD14** complex with its **LPS** moiety [13], [14]. Although **TLR4** is expressed in airway epithelial cells, it does not appear to be prominently involved in signaling of P. aeruginosa presented at the apical surface of airway epithelial cells [1], [2], [15], [16]. The low level of **MD-2** expression is also proposed to limit the responses of human airway epithelia to endotoxin stimulation [17].

TLR2 can also mediate **Beta-defensin 2** expression via **NF-kB** activation in response to bacterial antigens in human airway epithelia [18], thus promoting an effective immune response [1].

CFTR is a chloride channel that regulates chloride transport, fluid hydration and mucociliary clearance in the lung, thus preventing the bacterial growth in normal airways [1], [19], [20]. Normal **CFTR** promotes a rapid expression of **FasR(CD95)**, as well as an apoptotic response to P. aeruginosa infection (see: apoptotic process) [21].

Bacterial stimulation also leads to **FasR(CD95)**-dependent **NF-kB** activation [2], [22]. Rapid release of **IL-1 beta** (most probably **NF-kB**-dependent) is enhanced in the presence of functional **CFTR** in respiratory epithelial cells [22].

iNOS is expressed in normal human airway epithelium [23]. Both **NF-kB** and **IFN-gamma** signaling components are necessary for normal **iNOS** expression. **IFN-gamma** activates **JAK1** and **JAK2**/ **STAT1** signaling followed by **IRF1** and **iNOS** expression [24].

Maps and Descriptions [8 of 10]

Name	Input Objects ▲ p-value	Key Hubs p-value	Union Objects p-value
Development_Role of G-CSF in hematopoietic stem cell mobilization	0.004112	0.001325	2.42E-4

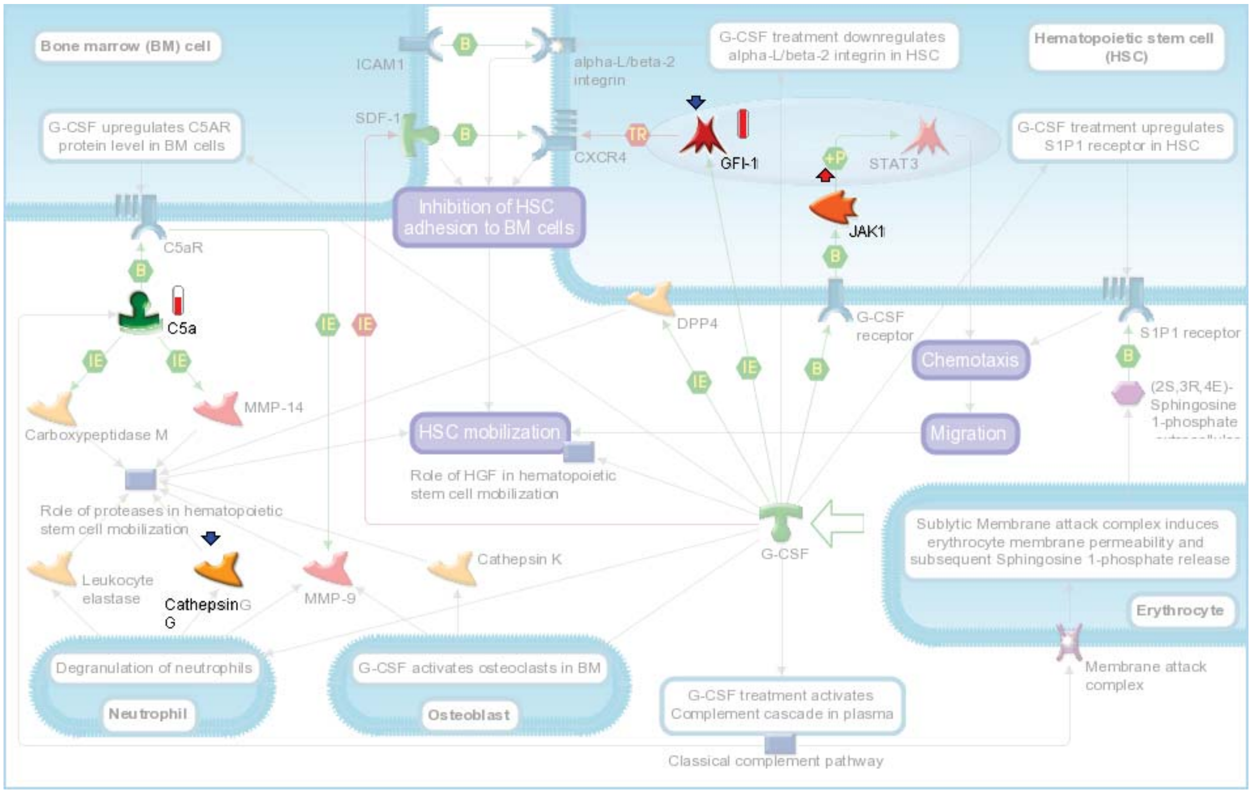

Abstract:

G-CSF-induced mobilization causes hematopoietic stem cell (HSC) egress from bone marrow (BM) niches and trafficking to the peripheral blood. The mechanisms underlying **G-CSF**-induced mobilization include activation of complement cascade, release of proteases in BM, disruption of adhesive contacts between HSCs and BM stromal cells and suppression of the **SDF-1**/ **CXCR4** axis.

Details:

Blood cell production is maintained by hematopoietic stem cells (HSCs) that reside in specialized niches within bone marrow (BM) [1]. Interaction of HSCs with BM cells (see cell-cell adhesion) and extracellular matrix (see cell-matrix adhesion) mediated by adhesion molecules, cytokines and chemokines underlie the retention of HSCs within BM niche [1], [2], [3], [4], [5]. Administration of chemotherapy, hematopoietic growth factors, chemokines, or small molecule inhibitors or antibodies against niche chemokine receptors and integrins causes HSC egress from BM niches and trafficking to the peripheral blood (PB), a process termed mobilization. Mobilization is applied to acquire HSCs for autologous and allogeneic transplantation. **G-CSF** is widely used clinically to mobilize HSCs for transplantation [1]. The mechanisms underlying **G-CSF**-induced HSC mobilization include complement activation, release of proteases in BM, disruption of adhesive contacts between HSCs and BM stromal cells (see negative regulation of cell-cell adhesion) and suppression of the **SDF-1**/ **CXCR4** axis [1], [4], [6].

SDF-1 is a key chemoattractant for HSCs which is constitutively expressed at high levels in BM and promotes HSC retention and maintenance within BM niche [6], [7]. **G-CSF** inhibits **SDF-1**/ **CXCR4** axis through downregulation of expression of both **SDF-1** on the surface of BM cells via unknown mechanism [8], [9] and its receptor **CXCR4** on the surface of HSCs [10] via induction of transcription repressor **GFI-1** [11]. Disruption of **SDF-1**/ **CXCR4** axis leads to inhibition of HSC adhesion to BM cells (see negative regulation of cell-cell adhesion) and HSC mobilization [1], [6].

G-CSF treatment also leads to downregulation of **alpha-L/beta-2 integrin** on the surface of HSCs [12] which results in inhibition of **alpha-L/beta-2 integrin**-**ICAM1**-dependent adhesion (see negative regulation of cell adhesion mediated by integrin) of HSCs to BM cells and HSC mobilization [2].

G-CSF binds to **G-CSF receptor** on HSCs and directly chemoattracts HSCs in a **JAK1**/ **STAT3**-dependent manner [13].

G-CSF treatment activates complement cascade pathway in plasma [14]. The complement activation results in release of anaphylatoxin **C5a** and **Membrane attack complex** [15], [16]. **Membrane attack complex** in sublytic concentrations induces erythrocyte membrane permeability and subsequent **(2S,3R,4E)-Sphingosine 1-phosphate** release from erythrocytes [15], [17], [18].

(2S,3R,4E)-Sphingosine 1-phosphate is a potent chemoattractant for HSCs [18], [19], [20]. It binds to **S1P1 receptor** on HSCs and induces chemotaxis and hematopoietic stem cell migration from BM to PB [15], [18], [20].

G-CSF upregulates **S1P1 receptor** expression in HSCs via unknown pathway [21].

In addition, **G-CSF** upregulates protein level of **C5aR**, receptor for **C5a**, in BM cells. **C5a** binds to **C5aR** and induces expression of proteases **Carboxypeptidase M**, **MMP-9** and **MMP-14** in BM cells [22], [23].

Moreover, **G-CSF** activates BM osteoclasts [24]. Activated osteoclasts release proteases **Cathepsin K** and **MMP-9** within BM niche [1], [25], [26]. In addition, **G-CSF** treatment leads to accumulation of neutrophils in BM and subsequent neutrophil degranulation which results in release of proteases such as **Leukocyte elastase**, **Cathepsin G** and **MMP-9** [4], [27], [28], [29].

Finally, **G-CSF** upregulates expression of protease **DPP4** in HSCs [30].

Proteases increase HSC mobilization via degradation of cell surface bound and extracellular matrix proteins which are responsible for HSC retention within BM [1], [4], [6].

Maps and Descriptions [9 of 10]

Name	Input Objects ▲ p-value	Key Hubs p-value	Union Objects p-value
Development_G-CSF-induced myeloid differentiation	0.008297	0.003777	9.974E-4

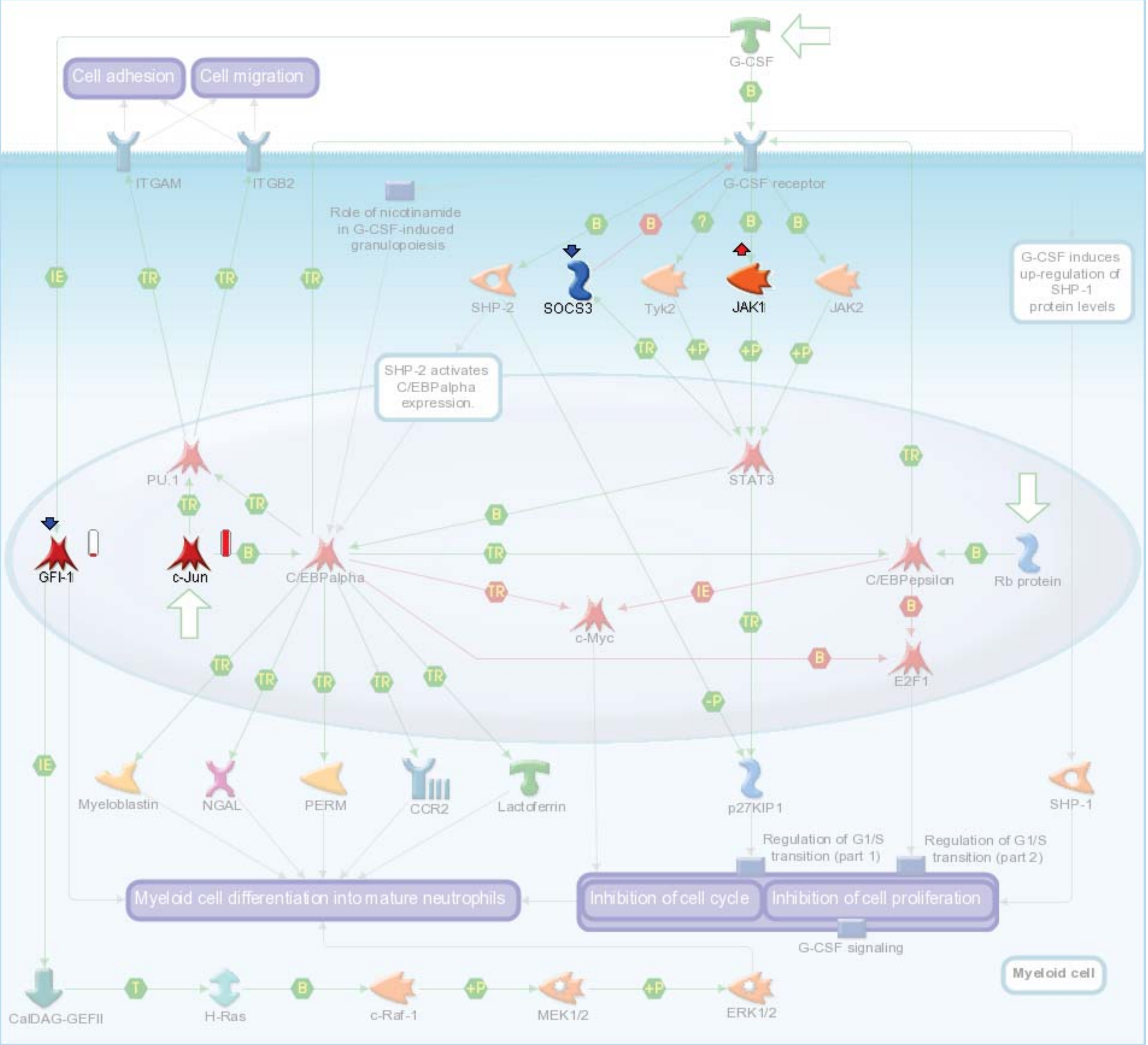

Description

G-CSF-induced myeloid differentiation

Abstract:

G-CSF is a major hematopoietic cytokine responsible for differentiation of myeloid cells into mature neutrophils. **G-SCF** exerts its function via action on **G-CSF receptor**. **G-CSF** induced myeloid cell differentiation through activation of Janus kinases (JAK)/ Signal transducer and activator of transcription (STAT) and **ERK1/2** pathways, as well as **SHP-2** activation. **G-CSF** realizes myeloid cell differentiation though inhibition of cell cycle and cell proliferation, and through activation of expression of myeloid-specific genes, which are regulated by **C/EBPalpha**, **C/EBPepsilon**, **PU.1** and **GFI-1**, the major transcription factors responsible for myeloid cell differentiation.

Details:

G-CSF is a major hematopoietic cytokine responsible for myeloid leukocyte differentiation into mature neutrophil. **G-CSF** exerts its function via action on **G-CSF receptor**, which is a single transmembrane-spanning protein lacking intrinsic kinase activity. Upon ligand binding, **G-CSF receptor** forms functional active homodimer [1], [2].

G-CSF binding to its receptor leads to activation of Janus kinases **JAK1**, **JAK2** and **Tyk2** [3], [4], [5], [6]. Janus kinases mainly phosphorylate **STAT3** [1]. **STAT3** is required for myeloid leukocyte differentiation into mature neutrophils [7], [8], [9], [10] and for regulation of **G-CSF receptor** signaling through activation of **SOCS3** transcription [1], [11], [12], [13]. **SOCS3** binds to **G-CSF receptor**, which leads to ubiquitination and subsequent lysosomal degradation of the receptor [13]. **SOCS3**-dependent control of **G-CSF receptor** lysosomal routing is required for an appropriate balance between proliferation and differentiation of myeloid progenitors in response to **G-CSF** [12], [13].

STAT3 controls cell cycle arrest of myeloid cells and their subsequent differentiation, at least partly, via transcriptional upregulation of **p27KIP1** [1], [14]. Also, **G-CSF receptor** binds to and activates **SHP-2**, which dephosphorylates and activates **p27KIP1**, what leads to inhibition of cell cycle and cell proliferation and subsequent myeloid leukocyte differentiation into mature neutrophils [15].

STAT3 also binds to and augments the transcriptional activity of **C/EBPalpha** [16]. In turn, **C/EBPalpha** in complex with **c-Jun** activates transcription of **PU.1** [17]. Both transcription factors, **PU.1** and **C/EBPalpha**, are required for **G-CSF**-induced granulocyte differentiation (see myeloid leukocyte differentiation) [16], [18], [19]. **PU.1** activates transcription of myeloid-specific genes **ITGAM** and **ITGB2** [18].

In addition, activated **G-CSF receptor** binds to **SHP-2** [9], [20], which stimulates **C/EBPalpha** expression [19]. In turn, **C/EBPalpha** activates expression of myeloid-specific genes **PERM** [19], [21], **Myeloblastin** [19], **Lactoferrin** [19], **NGAL** [16], [22], **CCR2** [21] and **G-CSF receptor** [19], [21], [23]. Also, **C/EBPalpha** can bind to and inhibit **E2F1** transcriptional activity [24], [25], [26] and down-regulate **c-Myc** expression [7], [27], thus inhibiting cell cycle and cell proliferation and promoting myeloid leukocyte differentiation.

C/EBPalpha also activates transcription of **C/EBPepsilon** [23], [28], [29], another transcription factor required for myeloid leukocyte differentiation [30], [31]. It is possible, that **Rb protein** increases **C/EBPepsilon** transcriptional activity during **G-CSF**-induced myeloid leukocyte differentiation, which leads to activation of transcription of **C/EBPepsilon** myeloid-specific target genes, such as **G-CSF receptor** [32], [33]. **C/EBPepsilon**, similar to **C/EBPalpha**, can bind to and inhibit **E2F1** transcriptional activity, as well as down-regulate **c-Myc** expression [34].

G-CSF stimulation also leads to **GFI-1** expression, which is also required for **G-CSF**-induced myeloid leukocyte differentiation [35], [36].

GFI-1 upregulates expression of **CalDAG-GEFII**, which activates **H-Ras**/ **c-Raf-1**/ **MEK1/2**/ **ERK1/2** cascade. Though **MEK1/2**/ **ERK1/2** signaling is known to promote cell proliferation, this pathway is also required for **G-CSF**-induced myeloid leukocyte differentiation. It is possible that different Rat sarcoma viral oncogene homolog (Ras) activators may lead to distinct downstream pathways [36], [37].

SHP-1 protein levels are upregulated during **G-CSF** stimulation, and **SHP-1** inhibits cell cycle and cell proliferation, allowing myeloid leukocyte differentiation [38].

Maps and Descriptions [10 of 10]

Name	Input Objects ▲ p-value	Key Hubs p-value	Union Objects p-value
Signal transduction_Additional pathways of NF-kB activation (in the nucleus)	0.008297	1.985E-8	3.392E-10

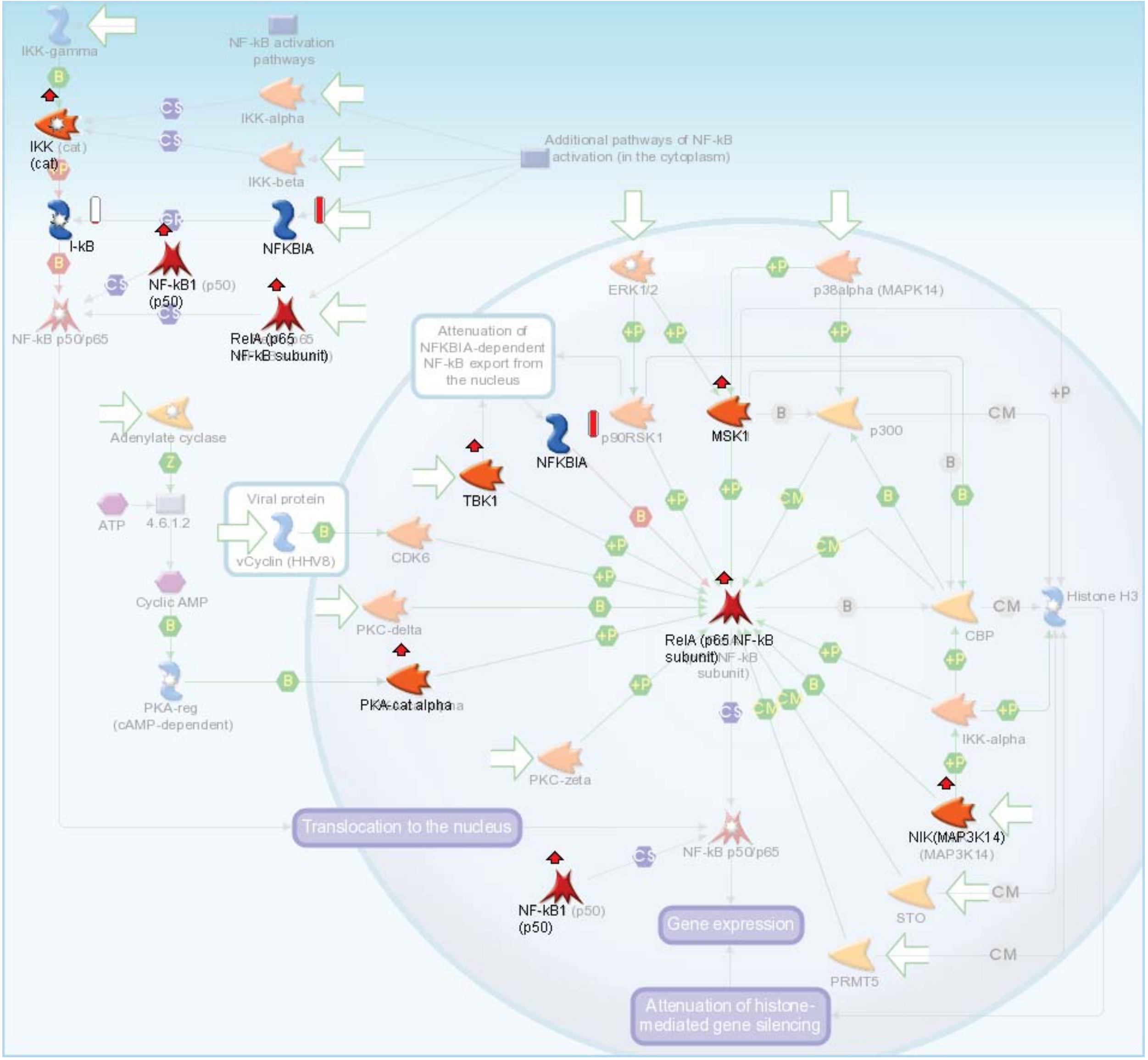

Abstract:

The main pathways of **NF-kB p50/p65** activation include activation of a kinase complex (IKK) that phosphorylates **I-kB** proteins inducing their degradation and **NF-kB p50/p65** translocation to the nucleus where it upregulates expression of numerous genes. In addition, multiple posttranslational modifications (PTMs) of the key signaling proteins including **RelA (p65 NF-kB subunit)** are required for **NF-kB p50/p65** activation. Several of these PTMs occur in the nucleus.

Details:

The transcription factor nuclear factor-kappa-B (NF-kB)/Rel family proteins regulate a wide range of host genes that govern the inflammatory response and immune response and play a critical role in controlling programmed cell death, cell proliferation and cell differentiation. In mammals, the NF-kB/Rel family consists of seven proteins, including RelA/p65 (**RelA (p65 NF-kB subunit)**), c-Rel, RelB, p100, p52, p105 and p50 (**NF-kB1 (p50)**) [1], [2], [3].

NF-kB p50/p65 is a typical NF-kB heterodimer composed of **NF-kB1 (p50)** and **RelA (p65 NF-kB subunit)**. In unstimulated cells, **NF-kB p50/p65** is sequestered in the cytoplasm by its association with inhibitory **I-kB** proteins. Stimulation of the cells by a variety of stimuli leads to the activation of a kinase complex (IKK) consisting of a non-catalytic (**IKK-gamma**) and two catalytic (**IKK-alpha** and/or **IKK-beta**, commonly called **IKK (cat)**) subunits. Activated **IKK (cat)** subunits phosphorylate **I-kB** proteins inducing **I-kB** protein K48-linked ubiquitination and degradation in the 26S proteasome (see: proteasome-mediated ubiquitin-dependent protein catabolic process). Subsequently, the liberated **NF-kB p50/p65** heterodimer translocates to the nucleus where it upregulates expression of multiple genes involved in different biological processes including inflammatory response, cell proliferation and cell survival (see: negative regulation of apoptotic process) [2], [3], [4], [5], [6], [7], [8].

The pathways that induce **NF-kB p50/p65** activation include post-translational protein modification of **RelA (p65 NF-kB subunit)** and can occur both in the cytoplasm and in the nucleus. These modifications play a key role in determining the duration and strength of NF-kB nuclear activity as well as its transcriptional output [3].

Different stimuli induce the classical **Adenylate cyclase**/ **Cyclic AMP**/ **PKA-reg (cAMP-dependent)**/ **PKA-cat alpha** pathway. As a result, **PKA-cat alpha** phosphorylates **RelA (p65 NF-kB subunit)** at Ser276, and thus enhances the overall transcriptional activity of NF-kB [3], [6], [9], [10]. Notably, **PKA-cat alpha** can phosphorylate **RelA (p65 NF-kB subunit)** both in the cytosol and in the nucleus [9], [10]. Moreover, **PKA-cat alpha** regulates the transcriptional activity of NF-kB by promoting its interaction with the transcriptional coactivator CBP/p300 [9].

MSK1 has been identified as a nuclear kinase for **RelA (p65 NF-kB subunit)** [11]. Activated **p38alpha (MAPK14)** and **ERK1/2** [12] directly phosphorylate **MSK1** at Ser360, Thr581 and Thr700 which is required for **MSK1** kinase activity [11], [13], [14], [15], [16]. In the nucleus, **MSK1** phosphorylates **RelA (p65 NF-kB subunit)** at Ser276, which enhances the transcriptional activity of NF-kB and allows efficient recruitment of the cofactors **CBP** and **p300** to the promoters of target genes [3], [11], [17]. **MSK1** also phosphorylates **Histone H3** at Ser11 (histone H3-S10 phosphorylation) and, probably, Ser29 (histone H3-S28 phosphorylation) in response to mitogenics, stress stimuli and epidermal growth factor [17], [18], [19], [20], [21].

In addition, **p38alpha (MAPK14)** controls the transcriptional activity of NF-kB by regulating acetylation of **RelA (p65 NF-kB subunit)** [22]. **p38alpha (MAPK14)**-dependent phosphorylation of acetyltransferase coactivator **p300** [22], [23] leads to subsequent association of **p300** with **RelA (p65 NF-kB subunit)** and acetylation of **RelA (p65 NF-kB subunit)** at Lys310 [22], [24], [25].

ERK1/2 also directly activates **p90RSK1** by phosphorylation [26], [27], [28], [29]. In turn, **p90RSK1** phosphorylates **RelA (p65 NF-kB subunit)** at Ser536 to increase NF-kB transcriptional activity [3], [30]. In addition, **p90RSK1**- or **TBK1**-mediated phosphorylation of Ser536 lowers **RelA (p65 NF-kB subunit)**'s affinity for **NFKBIA** and decreases **NFKBIA**-mediated nuclear export of NF-kB [3], [31], [32].

CDK6, which is stimulated by the viral protein **vCyclin (HHV8)** binding, phosphorylates **RelA (p65 NF-kB subunit)** at Ser536 both in the cytosol and in the nucleus [33].

PKC-zeta directly phosphorylates **RelA (p65 NF-kB subunit)** at Ser311 [34], [35], most probably in the nucleus [36], resulting in NF-kB transcriptional activation [34], [13].

PKC-delta also translocates from the cytoplasm into the nucleus and directly binds to **RelA (p65 NF-kB subunit)**. Although **PKC-delta** does not phosphorylate **RelA (p65 NF-kB subunit)**, its catalytic activity is required for orchestrating NF-kB transactivation via still unknown mechanism [37].

NIK(MAP3K14) is one of the main kinases required for **IKK-alpha** activation. **NIK(MAP3K14)** directly phosphorylates **IKK-alpha** at Ser176 and Ser180 within its activation loop [6], [38], [39]. In turn, **IKK-alpha** phosphorylates **RelA (p65 NF-kB subunit)** at Ser536 and this phosphorylation strongly increases NF-kB transcriptional activity [3], [40], [31]. Notably, **NIK(MAP3K14)** and **IKK-alpha** can function both in the cytosol and in the nucleus [3], [6], [38], [39], [40], [31]. In the nucleus, phosphorylation of **RelA (p65 NF-kB subunit)** at Ser536 by **IKK-alpha** [5], [31], [39] induces formation of enhanceosome, consisting of **NF-kB p50/p65** and transcriptional coactivators **CBP** and **p300** which also have intrinsic acetyltransferase activity catalyzing the acetylation of lysine residues in histone and non-histone proteins [39], [41], [42], [43], [44] (see: histone acetylation; protein acetylation; peptidyl-lysine acetylation).

IKK-alpha phosphorylates **CBP** at Ser1382 and Ser1386 and consequently increases **CBP**'s histone acetyltransferase and transcriptional activity. Importantly, such phosphorylation enhances NF-kB-mediated gene expression [45]. The **CBP**/**p300** acetyltransferase complex acetylates **RelA (p65 NF-kB subunit)** at seven known sites, including Lys122, Lys123 [46], Lys218, Lys221, Lys310 [23], [24], Lys314 and Lys315 [47]. The acetylation of each of these lysines appears to regulate different biological properties of the NF-kB transcription factor [48]. Lys221 acetylation enhances DNA-binding activity of NF-kB *in vitro* and abolishes the interaction with **NFKBIA** leading to a prolonged NF-kB response in the nucleus. The acetylation at Lys310 is required for full transcriptional activity of **RelA (p65 NF-kB subunit)** [24]. In contrast to Lys218, Lys221 and Lys310, acetylation of Lys122 and Lys123 decreases the DNA binding of **RelA (p65 NF-kB subunit)** facilitating its removal from the nucleus resulting in a faster termination of the NF-kB response [46]. Although general transcriptional activity of **RelA (p65 NF-kB subunit)** is not affected by acetylation at Lys314 and Lys315, the expression of specific sets of genes is differentially modulated (either up- or down-regulated) by lysine-specific acetylation of **RelA (p65 NF-kB subunit)** [47].

In addition, **CBP** and **p300** acetylate histones, including acetylation of **Histone H3** [49] at Lys15 (histone H3-K14 acetylation) [49], [50], [51], [52], [53], [54], [55], Lys19 (histone H3-K18 acetylation) [49], [53], [56], Lys28 (histone H3-K27 acetylation) [56], [57], and promote acetylation of **Histone H3** at Lys10 (histone H3-K9 acetylation) [39], [52], [54], [58], [59]. **CBP** and **p300** also acetylate **Histone H3** at Lys57 (histone H3-K56 acetylation), but this site-specific acetylation is unlikely involved in gene expression; however, it plays a critical role in DNA repair [60]. **IKK-alpha** phosphorylates **Histone H3** at Ser11 (histone H3-S10 phosphorylation), and thus promotes its subsequent acetylation. Phosphorylation and acetylation of histones leads to chromatin remodeling and attenuation of histone-mediated gene silencing allowing **NF-kB p50/p65**-dependent transcription of target genes [39], [51].

STO, a histone H3K36 methyltransferase [3], can also monomethylate Lys218 and dimethylate Lys221 of **RelA (p65 NF-kB subunit)** enhancing the transcriptional activity of NF-kB and the expression of NF-kB target genes [3], [61].

Finally, **PRMT5**, that usually methylates histones [62], [63], dimethylates Arg30 of **RelA (p65 NF-kB subunit)** to increase NF-kB activity [64].

Diseases (by Biomarkers)

This ontology is created based on the classification in Medical Subject Headings (MeSH). Each disease in diseases ontology has its corresponding biomarker gene or set of genes annotated manually from the literature.

	Diseases (by Biomarkers) Details [61 processes]			
#	Name	Input Objects ▲ p-value	Key Hubs p-value	Union Objects p-value
1	Hypersensitivity	3.122E-10	9.117E-4	1.728E-10
2	Hypersensitivity, Immediate	3.701E-9	0.001353	1.388E-9
3	Lung Diseases, Obstructive	6.46E-9	0.002517	5.429E-9
4	Inflammation	1.289E-8	4.198E-6	1.509E-12
5	Lymphoma	4.978E-7	1.643E-5	1.273E-10
6	Infection	7.345E-7	3.067E-7	6.592E-12
7	Virus Diseases	1.979E-6	5.913E-5	1.878E-9
8	Lymphoma, Non-Hodgkin	2.213E-6	4.899E-4	2.185E-8
9	Bacterial Infections and Mycoses	3.185E-6	9.69E-7	7.46E-11
10	Rheumatic Diseases	3.631E-6	2.259E-5	9.634E-10
11	Arthritis	8.41E-6	1.982E-6	1.199E-10
12	Lupus Erythematosus, Systemic	1.089E-5	2.584E-7	1.569E-11
13	Joint Diseases	1.108E-5	3.06E-6	2.439E-10
14	RNA Virus Infections	1.197E-5	3.088E-4	4.817E-8
15	Arthritis, Rheumatoid	1.574E-5	6.59E-5	1.023E-8
16	Pathologic Processes	1.957E-5	1.105E-7	1.105E-11
17	Inflammatory Bowel Diseases	2.003E-5	0.003362	1.305E-6
18	Lymphoma, B-Cell	2.213E-5	0.001221	3.038E-7
19	Crohn Disease	2.54E-5	0.005173	2.284E-6
20	Gastroenteritis	5.778E-5	0.002048	1.517E-6
21	Connective Tissue Diseases	7.43E-5	8.096E-8	2.644E-11
22	Autoimmune Diseases	7.994E-5	5.186E-8	1.786E-11
23	Respiratory Tract Infections	9.279E-5	0.009325	1.197E-5
24	Suppuration	1.383E-4	0.001792	8.829E-7
25	Immune System Diseases	3.802E-4	1.564E-7	2.261E-10
26	Carcinoma, Bronchogenic	8.677E-4	3.325E-7	1.153E-9
27	Bronchial Neoplasms	8.819E-4	3.455E-7	1.218E-9
28	Lung Diseases	8.899E-4	2.129E-4	1.134E-6
29	Vascular Diseases	0.001078	4.203E-7	1.797E-9
30	Lymphatic Diseases	0.001347	0.001455	1.148E-5
31	Respiratory Tract Diseases	0.001367	4.532E-4	3.631E-6
32	Lymphoproliferative Disorders	0.001715	1.532E-6	1.027E-8
33	Glomerulonephritis	0.002131	4.774E-4	4.29E-6

Diseases (by Biomarkers) Details [61 processes]				
#	Name	Input Objects ▲ p-value	Key Hubs p-value	Union Objects p-value
34	Lymphoma, Large B-Cell, Diffuse	0.002573	7.282E-5	5.761E-7
35	Nephritis	0.002781	7.437E-4	8.682E-6
36	Immunoproliferative Disorders	0.002862	1.844E-6	2.052E-8
37	Musculoskeletal Diseases	0.003238	0.001374	2.136E-5
38	Prostatic Intraepithelial Neoplasia	0.003352	0.003046	4.156E-5
39	Breast Neoplasms	0.00346	1.803E-5	2.154E-7
40	Breast Diseases	0.00347	1.814E-5	2.174E-7
41	Lung Neoplasms	0.004281	0.001891	4.346E-5
42	Multiple Myeloma	0.004377	6.998E-5	1.196E-6
43	Thoracic Neoplasms	0.004436	0.00201	4.774E-5
44	Paraproteinemias	0.004516	3.395E-5	5.914E-7
45	Neoplasms, Plasma Cell	0.004516	7.48E-5	1.318E-6
46	Cardiovascular Diseases	0.004594	6.94E-7	1.287E-8
47	Neurilemmoma	0.005302	0.006946	1.412E-4
48	Blood Protein Disorders	0.005348	4.953E-5	1.016E-6
49	Hemic and Lymphatic Diseases	0.005497	2.113E-5	8.163E-7
50	Neuroma	0.005541	0.007371	1.565E-4
51	Respiratory Tract Neoplasms	0.005671	0.003058	9.115E-5
52	Hemostatic Disorders	0.005766	1.257E-4	2.802E-6
53	Hematologic Diseases	0.005993	7.126E-6	3.446E-7
54	Colitis, Ulcerative	0.006554	0.001152	2.904E-5
55	Carcinoma, Non-Small-Cell Lung	0.006659	9.002E-8	2.736E-9
56	Neurofibroma	0.007033	0.00812	2.389E-4
57	Carcinoma, Ductal, Breast	0.007202	2.063E-6	5.795E-8
58	Colonic Diseases	0.007403	4.635E-6	1.325E-7
59	Colitis	0.008067	0.001665	5.129E-5
60	Neoplasms, Ductal, Lobular, and Medullary	0.008522	5.124E-7	1.846E-8
61	Hemorrhagic Disorders	0.008629	6.793E-5	2.199E-6

Process Networks

A recognized series of events (interactions or biochemical reactions) accomplished by one or more ordered assemblies of molecular functions with a defined beginning and end.

Process Networks Details [6 processes]				
#	Name	Input Objects ▲ p-value	Key Hubs p-value	Union Objects p-value
1	Cell cycle_G1-S Interleukin regulation	1.744E-5	0.008261	2.547E-6
2	Proliferation_Lymphocyte proliferation	3.86E-4	0.001338	3.086E-6
3	Inflammation_IL-2 signaling	5.607E-4	5.488E-4	1.288E-6
4	Inflammation_NK cell cytotoxicity	6.582E-4	0.002772	1.036E-5
5	Immune response_Antigen presentation	0.001706	0.00912	8.97E-5
6	Inflammation_IL-10 anti-inflammatory response	0.002457	0.004724	4.719E-5

Map Folders

This is a collection of manually created pathway maps, grouped hierarchically into folders according to main biological processes. A map could participate in different folders if depicted pathway takes part in different main biological processes (Folders).

	Map Folders Details [23 processes]			
#	Name	Input Objects ▲ p-value	Key Hubs p-value	Union Objects p-value
1	Immune system response	3.283E-12	2.798E-7	5.933E-16
2	Dermatitis, Allergic Contact	2.424E-10	2.683E-5	8.879E-13
3	Systemic Lupus Erythematosus	1.526E-9	7.006E-6	2.229E-12
4	Asthma	8.847E-9	1.533E-7	1.964E-14
5	Inflammatory response	1.37E-6	1.068E-4	6.908E-9
6	Neurodegeneration in Multiple sclerosis	2.506E-6	8.815E-5	2.773E-9
7	Neurofibromatoses	3.905E-5	3.089E-6	1.617E-9
8	Colorectal Neoplasms	4.092E-5	3.873E-6	6.607E-10
9	Multiple myeloma	1.225E-4	5.335E-5	3.059E-8
10	Apoptosis	1.301E-4	1.969E-5	3.195E-8
11	Pancreatic Neoplasms	1.362E-4	0.007568	1.24E-5
12	Prostatic Neoplasms	3.702E-4	0.002863	7.301E-6
13	Inflammatory diseases	6.083E-4	0.001175	3.586E-6
14	Hematology	0.001117	4.161E-4	1.887E-6
15	Ovarian cancer	0.001359	3.482E-6	3.962E-8
16	Transcription regulation	0.001471	8.743E-7	1.605E-8
17	Cystic fibrosis disease	0.001575	0.008902	8.065E-5
18	Cell differentiation	0.00158	2.211E-5	1.103E-7
19	Cell cycle and its regulation	0.003121	0.004239	5.721E-5
20	Lung cancer	0.005887	5.157E-4	9.428E-6
21	Breast Neoplasms	0.008041	7.777E-4	1.934E-5
22	Tissue remodeling and wound repair	0.009368	1.431E-4	4.089E-6
23	Carcinoma, Hepatocellular	0.00948	7.777E-6	2.311E-7

Appendix 1: Legend

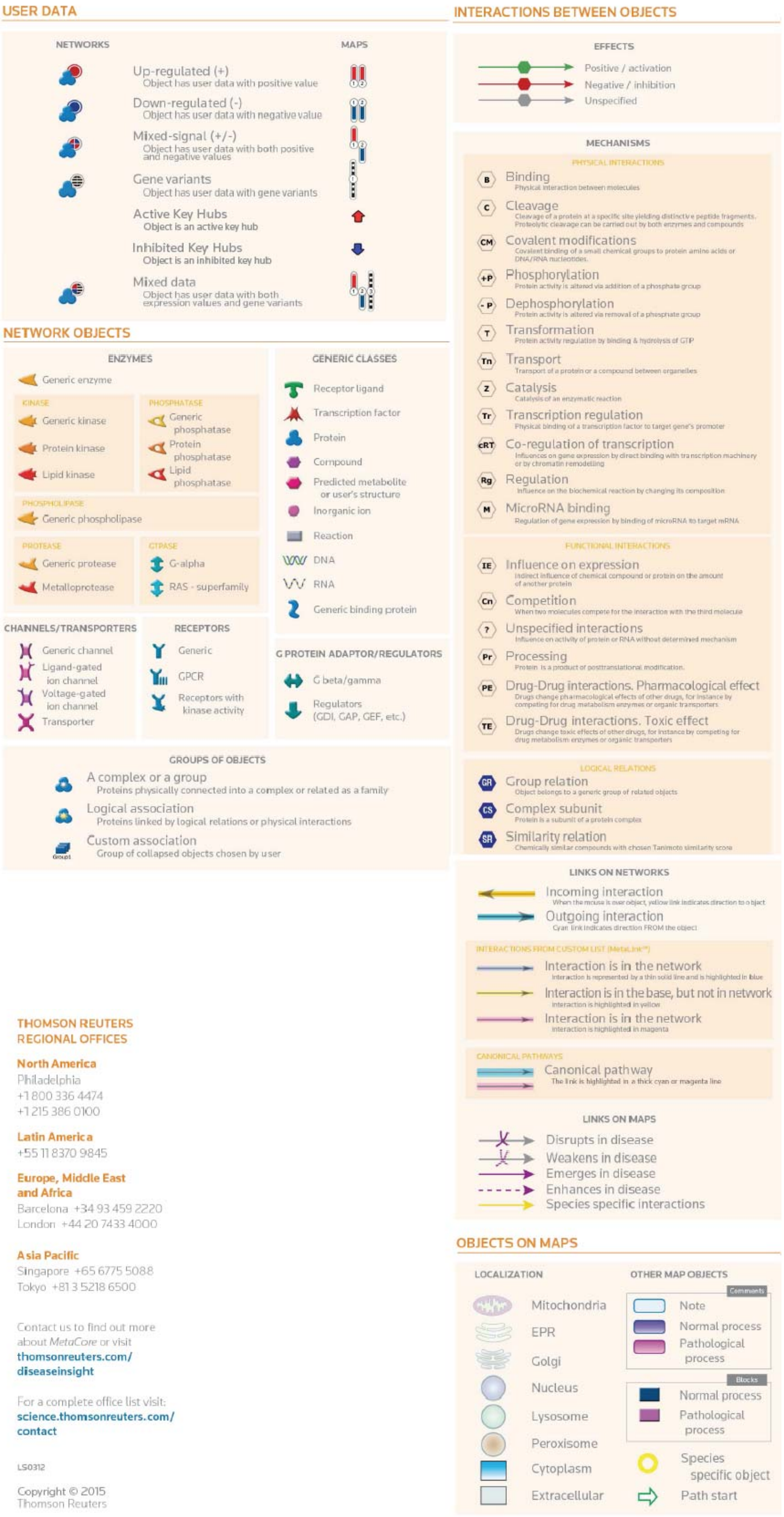

Appendix 2: Glossary

Processes Networks
A recognized series of events (interactions or biochemical reactions) accomplished by one or more ordered assemblies of molecular functions with a defined beginning and end.
Diseases (by Biomarkers)
This ontology is created based on the classification in Medical Subject Headings (MeSH). Each disease in diseases ontology has its corresponding biomarker gene or set of genes.
Disease Biomarker Networks
Manually created network models of diseases with disease biomarkers as seed nodes. The networks are organized in a folder tree. Each folder contains one or more networks for a disease. The name of the folder is the name of the disease.
Drug Target Networks (Drug Action Mechanisms)
Manually created networks with drug targets as seed nodes. These processes are derived from Processes networks that are targetable processes with genes that function coordinately when treated with drug.
Enrichment Analysis (EA) (also, Ontology Enrichment)
An analysis procedure that consists of mapping gene IDs of the dataset(s) of interest onto gene IDs in processes (terms) of built-in functional ontologies such as pathway maps, networks,diseases, etc. The terms in a given ontology are ranked based on “relevance” in the dataset. The statistical relevance procedure, a p-value of hypergeometric distribution, is calculated as the probability of a match to occur by chance, given the size of the ontology, the dataset and the particular process. The lower the p-value, the higher is the 'non-randomness' of finding the intersection between the dataset and the particular ontology term. That, in turn, translates into a higher ranking for the process matched. Everything equal, the more genes/proteins belong to a process/pathway, the lower the p-value. In EA we use multiple proprietary ontologies (canonical pathway maps, cellular processes, toxicities, disease biomarkers etc., and public ontologies such as Gene Ontology (cellular processes, protein functions, localizations).
Enrichment Synergy
The enrichment synergy method was offered for comparison of datasets that are functionally relevant but poorly overlapping at the gene level, for instance mutated and amplified genes in breast cancer [5]. The genes derived from different datasets may populate the very same pathway or process, which suggests that they are functionally complimentary. To determine whether two distinct gene lists cooperatively alter a certain cellular pathway or process, we calculate the synergy between them by ontology enrichment. An ontology term (pathway or process) is considered synergistic if the enrichment p-value for the non-redundant union of compared gene lists is lower than p-values for individual lists. More significant enrichment for the union reflects functional connectivity of two gene lists and their complementary effect on the pathway.
Process
An element or an term in an ontology, e.g., a given disease, or a given process, etc.
GO Localizations
A GO ontology for localization of the gene products inside or outside the cell. A given molecule in a given localization is represented by a network object in MetaCore™.
GO Molecular Functions
A GO ontology of hierarchically structured molecular functions. A protein may be linked to several different molecular functions.
GO Processes
A GO ontology for biological processes. The processes are structured as hierarchical tree with branches defined according to the Gene Ontology controlled vocabulary. GO process folders are nested, i.e., each folder references all the proteins participating in its sub-processes.

Key Hub (KH)
A topologically significant network object that supposed to regulate differential expression genes. KHs could be obtained by two approaches: causal reasoning network analysis and overconnectivity analysis. Using causal resoning the one could define one step KHs (transcriptional factors that statistically significant associated with experimental differential expressed genes regulation) and distant KHs (second step objects regulate one step transcriptional factors, etc, up to four steps). Overconnectivity analysis gives network objects that are overconnected with experimental differentially expressed genes.
Key Process
An ontology term (i.e. pathway maps) that enriched with both differentially expressed genes and corresponding key Hubs (see Introduction part for detailed workflow description).
Map Folder
This is a collection of manually created pathway maps, grouped hierarchically into folders according to main biological processes. Click on the folder name to open the folder in the new window and see maps.
Metabolic Networks
Metabolic Networks represent a reconstruction of metabolic processes.
Network Object
A process that describes the type of molecule, e.g. kinases, transcriptional factors, receptors, etc.
Ontology
Functional ontologies developed for biological processes, toxic processes, disease biomarkers, diseases, drug targets and drug action mechanisms. Each ontology has hierarchical tree structure and each has corresponding sets of pre-built networks and pathway maps, or, in case of disease biomarkers, gene lists.
Pathway Map
Pathway maps are graphic images representing complete biochemical pathways or signaling cascades in a commonly accepted sense. They are drawn by experts using Pathway Map Creator™ tool. Typically, a map comprises 3-5 MetaCore™ pathways. Maps are assembled into map folders divided onto regulatory, metabolic, disease, toxicity and drug action sections, and thus form an ontology of their own kind. Maps are interactive and hyperlinked to annotation pages for all objects displayed on them (genes, proteins, compounds and interactions).
Toxicity Networks
Toxicity networks are models of toxicity-related processes.

Appendix 3: List of Key Hubs IDs

For uploaded DEG lists, a causal reasoning test was performed to identify statistically significant network objects (p-value < 0.01). These proteins can be considered as topologically significant direct and indirect upstream regulators of the input genes (up to four steps from the DEG subset). First step regulators always are transcriptional factors while the other more distant regulators could be different regulatory proteins (Number of step from a significant regulator to DEG subset is defined in Distance column).

Key Hubs - Causal Reasoning						
#	Network Object	Molecular Function	Object Activity	Correct/All predictions	p-value ▲	Distance
1	SCAI	Generic protein	-	16/17	0.0001373	3
2	MKL1	Transcription factor	+	16/17	0.0001373	2
3	MBD6	Generic binding protein	-	21/24	0.0001386	3
4	SUPT16H	Metalloprotease	+	18/20	0.0002012	3
5	DLX4 (BP1)	Transcription factor	-	12/12	0.0002441	2
6	TRAF1	Generic binding protein	+	12/12	0.0002441	3
7	TRAF5	Generic binding protein	+	12/12	0.0002441	3
8	MafK	Transcription factor	+	12/12	0.0002441	2
9	CUX1	Transcription factor	-	15/16	0.0002594	2
10	BRN4	Transcription factor	+	15/16	0.0002594	3
11	ChAF1 subunit A	Generic binding protein	-	17/19	0.0003643	3
12	PKA-cat alpha	Protein kinase	+	14/15	0.0004883	2
13	ZAK	Protein kinase	+	14/15	0.0004883	3
14	miR-148a-3p	RNA	-	14/15	0.0004883	2
15	PRDX1	Generic enzyme	+	11/11	0.0004883	2
16	SRX1	Generic binding protein	+	11/11	0.0004883	3
17	AKAP28	Generic binding protein	+	14/15	0.0004883	3
18	SLM-1	Generic binding protein	+	11/11	0.0004883	3
19	Rab-13	RAS superfamily	-	14/15	0.0004883	3
20	Sequestosome 1(p62)	Generic binding protein	-	14/15	0.0004883	2
21	Par-4	Generic binding protein	-	11/11	0.0004883	2
22	IKK (cat)	Protein kinase	+	11/11	0.0004883	2
23	SAM68	Generic binding protein	+	11/11	0.0004883	2
24	COMMD1 (MURR1)	Transporter	-	11/11	0.0004883	2
25	MSK1	Protein kinase	+	14/15	0.0004883	2
26	PLC-beta3	Generic phospholipase	-	16/18	0.0006561	3

Key Hubs - Causal Reasoning						
#	Network Object	Molecular Function	Object Activity	Correct/All predictions	p-value ▲	Distance
27	SOX11	Transcription factor	+	16/18	0.0006561	2
28	SOX11	Transcription factor	+	22/27	0.0007569	3
29	CFTR	Generic channel	-	13/14	0.0009155	3
30	IRF2	Transcription factor	-	13/14	0.0009155	2
31	SPHK2	Lipid kinase	+	10/10	0.0009766	2
32	miR-142-5p	RNA	-	10/10	0.0009766	2
33	Karyopherin alpha 3	Transporter	+	10/10	0.0009766	3
34	DAB1	Generic binding protein	+	10/10	0.0009766	3
35	NFKBIB	Generic binding protein	-	10/10	0.0009766	2
36	Cathepsin G	Generic protease	-	10/10	0.0009766	3
37	TRPS1	Transcription factor	-	25/32	0.001051	3
38	FKBP4	Generic binding protein	+	25/32	0.001051	3
39	ZNF639	Generic binding protein	+	15/17	0.001175	3
40	miR-199a-5p	RNA	-	15/17	0.001175	2
41	RN7SK	RNA	-	15/17	0.001175	3
42	miR-663a	RNA	-	21/26	0.001247	3
43	miR-106a-3p	RNA	-	17/20	0.001288	3
44	miR-22-3p	RNA	-	17/20	0.001288	2
45	HSC70	Generic enzyme	+	17/20	0.001288	2
46	SGTB	Generic binding protein	+	17/20	0.001288	3
47	miR-3120-5p	RNA	-	17/20	0.001288	3
48	DNAJB12	Generic binding protein	+	17/20	0.001288	3
49	miR-3120-3p	RNA	-	17/20	0.001288	3
50	GSC	Transcription factor	-	26/34	0.001468	3
51	HMG20B	Transcription factor	-	24/31	0.001663	3
52	PEAR1	Generic protein	+	12/13	0.001709	3
53	TRPS1	Transcription factor	-	12/13	0.001709	2
54	TRAF7	Generic enzyme	-	12/13	0.001709	2
55	PPME1	Generic enzyme	+	12/13	0.001709	3
56	PP2A catalytic	Protein phosphatase	-	12/13	0.001709	2
57	Bcl-6	Transcription factor	-	22/28	0.00186	3
58	CEGP1	Generic binding protein	-	9/9	0.001953	3
59	CD33	Generic receptor	-	9/9	0.001953	3
60	FAM21A	Generic phosphatase	+	9/9	0.001953	2

Key Hubs - Causal Reasoning						
#	Network Object	Molecular Function	Object Activity	Correct/All predictions	p-value ▲	Distance
61	Nucleoredoxin	Generic enzyme	+	9/9	0.001953	3
62	KIR2DL2	Generic receptor	-	9/9	0.001953	3
63	ABCG2	Generic channel	-	9/9	0.001953	3
64	AZI2	Generic protein	+	9/9	0.001953	3
65	UACA	Generic binding protein	-	9/9	0.001953	2
66	IRAK1BP1	Generic binding protein	+	9/9	0.001953	2
67	DVL-1	Generic binding protein	-	9/9	0.001953	2
68	CD244	Generic receptor	-	9/9	0.001953	3
69	SRRF	Generic binding protein	-	9/9	0.001953	3
70	Nkx2.8	Transcription factor	+	9/9	0.001953	3
71	Tubulin gamma 1	Generic binding protein	+	9/9	0.001953	3
72	MMP-24	Metalloprotease	+	9/9	0.001953	3
73	Kir2.2	Generic channel	+	9/9	0.001953	2
74	PIPKI gamma	Generic kinase	-	9/9	0.001953	3
75	PTPR-mu	Generic receptor	-	9/9	0.001953	3
76	SIGLEC5	Generic receptor	-	9/9	0.001953	3
77	Sec5	Generic binding protein	+	9/9	0.001953	3
78	Cullin 2	Generic binding protein	-	9/9	0.001953	2
79	HSPA1B	Generic binding protein	-	9/9	0.001953	2
80	UCHL3	Generic protease	-	9/9	0.001953	3
81	Prolargin	Generic binding protein	-	9/9	0.001953	2
82	HURP	Protein phosphatase	+	9/9	0.001953	2
83	ZNF537	Transcription factor	-	9/9	0.001953	2
84	CRIP2	Generic binding protein	-	9/9	0.001953	2
85	BTLA	Generic receptor	-	9/9	0.001953	3
86	MPP8	Generic binding protein	+	9/9	0.001953	3
87	AKIP1	Generic binding protein	-	9/9	0.001953	2
88	miR-26b-3p	RNA	-	9/9	0.001953	2
89	Fc gamma RII beta	Generic receptor	-	9/9	0.001953	3
90	DDX1	Generic enzyme	+	9/9	0.001953	2
91	IL-33	Receptor ligand	-	9/9	0.001953	2

Key Hubs - Causal Reasoning						
#	Network Object	Molecular Function	Object Activity	Correct/All predictions	p-value ▲	Distance
92	CtBP	Generic binding protein	-	9/9	0.001953	2
93	DPF3	Generic binding protein	+	9/9	0.001953	2
94	Rab-28	RAS superfamily	+	9/9	0.001953	2
95	E-cadherin	Generic binding protein	-	9/9	0.001953	2
96	Clusterin	Generic binding protein	-	9/9	0.001953	2
97	LAIR1	Generic receptor	-	9/9	0.001953	3
98	Pyrin (MEFV)	Generic binding protein	+	9/9	0.001953	2
99	SETD6	Generic protein	-	9/9	0.001953	2
100	IL-2 receptor	Generic receptor	+	9/9	0.001953	3
101	TBK1	Protein kinase	+	9/9	0.001953	2
102	ATRIP	Generic binding protein	+	9/9	0.001953	3
103	DYNC1I1	Generic binding protein	+	9/9	0.001953	2
104	RIG-G	Generic binding protein	+	9/9	0.001953	3
105	P-cadherin	Generic binding protein	-	9/9	0.001953	3
106	CSNK1G1	Protein kinase	-	9/9	0.001953	2
107	KIR3DL1	Generic receptor	-	9/9	0.001953	3
108	NKG2A	Generic receptor	-	9/9	0.001953	3
109	OGG1	Generic enzyme	+	9/9	0.001953	2
110	miR-345-5p	RNA	-	9/9	0.001953	2
111	IEX1	Generic protein	-	9/9	0.001953	2
112	MOX2	Transcription factor	-	9/9	0.001953	2
113	CDK5RAP3	Generic binding protein	-	9/9	0.001953	2
114	NKD2	Generic binding protein	+	9/9	0.001953	3
115	Ccd1	Generic binding protein	+	9/9	0.001953	3
116	Pinin	Transcription factor	-	9/9	0.001953	3
117	DTX4	Generic binding protein	-	9/9	0.001953	3
118	TRF2	Generic binding protein	+	9/9	0.001953	3
119	Siglec-E	Generic receptor	-	9/9	0.001953	3
120	Annexin I	Generic binding protein	-	9/9	0.001953	2
121	CD84	Generic binding protein	-	9/9	0.001953	3

Key Hubs - Causal Reasoning						
#	Network Object	Molecular Function	Object Activity	Correct/All predictions	p-value ▲	Distance
122	KIR2DL1	Generic receptor	-	9/9	0.001953	3
123	PRMT7	Generic enzyme	+	9/9	0.001953	3
124	BVRA	Generic enzyme	+	9/9	0.001953	2
125	PIRB	Generic receptor	-	9/9	0.001953	3
126	Copine-1	Transporter	-	9/9	0.001953	2
127	RelA (p65 NF-kB subunit)	Transcription factor	+	9/9	0.001953	1
128	ILT4	Generic receptor	-	9/9	0.001953	3
129	RNF10	Generic binding protein	-	9/9	0.001953	3
130	GNT-III	Generic enzyme	-	9/9	0.001953	3
131	UBC	Generic binding protein	-	9/9	0.001953	2
132	ELF3	Transcription factor	+	14/16	0.00209	2
133	HEXIM2	Generic binding protein	-	14/16	0.00209	3
134	CDK9	Protein kinase	+	14/16	0.00209	2
135	ELF1	Transcription factor	+	14/16	0.00209	2
136	miR-373-3p	RNA	-	14/16	0.00209	2
137	microRNA 203	RNA	+	16/19	0.002213	3
138	LCMT1	Generic enzyme	-	16/19	0.002213	3
139	miR-127-5p	RNA	+	16/19	0.002213	3
140	miR-211-3p	RNA	-	16/19	0.002213	3
141	KLF6	Transcription factor	+	16/19	0.002213	3
142	PACERR	RNA	-	21/27	0.002962	3
143	RPA1	Generic binding protein	+	21/27	0.002962	3
144	MKL1	Transcription factor	+	21/27	0.002962	3
145	NF-kB1 (p50)	Transcription factor	+	21/27	0.002962	2
146	DEC2	Transcription factor	-	11/12	0.003174	2
147	DCTN2	Generic binding protein	-	11/12	0.003174	2
148	miR-182-5p	RNA	-	11/12	0.003174	2
149	DDA3	Generic binding protein	-	11/12	0.003174	3
150	miR-1301-3p	RNA	-	11/12	0.003174	3
151	microRNA 138-1	RNA	+	11/12	0.003174	3
152	DDX42	Generic enzyme	-	11/12	0.003174	3
153	KLF6	Transcription factor	+	11/12	0.003174	2
154	SFMBT1	Generic protein	+	11/12	0.003174	3
155	FBXL11	Generic enzyme	-	11/12	0.003174	2

Key Hubs - Causal Reasoning

#	Network Object	Molecular Function	Object Activity	Correct/All predictions	p-value ▲	Distance
156	RPS3	Generic binding protein	+	11/12	0.003174	2
157	LYRIC	Generic binding protein	+	11/12	0.003174	2
158	ASPP2	Generic binding protein	+	11/12	0.003174	2
159	FALZ	Transcription factor	+	19/24	0.003305	3
160	CKIP-1	Generic protein	-	17/21	0.003599	3
161	Bcl-6	Transcription factor	-	17/21	0.003599	2
162	CHL1	Generic binding protein	+	17/21	0.003599	3
163	ZXDC	Transcription factor	+	17/21	0.003599	3
164	PIAS1	Generic enzyme	-	17/21	0.003599	2
165	MAGE-1 antigen	Generic protein	+	17/21	0.003599	3
166	miR-212-5p	RNA	+	17/21	0.003599	3
167	Elongin B	Generic protein	-	13/15	0.003693	3
168	L3MBTL2	Generic binding protein	-	15/18	0.003769	3
169	ADAM19	Metalloprotease	-	8/8	0.003906	3
170	TAF15	Generic binding protein	-	8/8	0.003906	2
171	FLJ11305	Generic binding protein	-	8/8	0.003906	2
172	HBXAP	Generic binding protein	+	8/8	0.003906	2
173	PDGF-AB	Receptor ligand	+	8/8	0.003906	3
174	miR-449b-5p	RNA	+	8/8	0.003906	2
175	PDGF-D	Receptor ligand	+	8/8	0.003906	3
176	CaMK IV	Protein kinase	+	8/8	0.003906	2
177	REV-ERB-BETA	Transcription factor	-	8/8	0.003906	2
178	LYVE-1	Generic receptor	+	8/8	0.003906	3
179	Adenosine A2b receptor	GPCR	-	8/8	0.003906	2
180	Ankyrin-B	Generic binding protein	-	8/8	0.003906	3
181	miR-199b-5p	RNA	-	8/8	0.003906	2
182	Annexin VI	Generic binding protein	+	8/8	0.003906	2
183	PDGF-C	Receptor ligand	+	8/8	0.003906	3
184	A2M	Receptor ligand	+	8/8	0.003906	3
185	NCK2 (Grb4)	Generic binding protein	+	8/8	0.003906	3
186	ADA	Generic enzyme	-	8/8	0.003906	3
187	PDGF-B	Receptor ligand	+	8/8	0.003906	3

Key Hubs - Causal Reasoning

#	Network Object	Molecular Function	Object Activity	Correct/All predictions	p-value ▲	Distance
188	HMG20A	Transcription factor	+	8/8	0.003906	3
189	HMG20B	Transcription factor	-	8/8	0.003906	2
190	RGS12	Regulators (GDI, GAP, GEF etc.)	-	8/8	0.003906	3
191	PDGF-R-beta	Receptor with enzyme activity	+	8/8	0.003906	2
192	HMGB1	Transcription factor	+	20/26	0.004678	3
193	microRNA 17	RNA	-	20/26	0.004678	3
194	miR-320c	RNA	-	18/23	0.005311	3
195	Syk	Protein kinase	+	18/23	0.005311	3
196	NEDD4L	Generic enzyme	-	23/31	0.005337	3
197	miR-125a-5p	RNA	-	10/11	0.005859	2
198	miR-7-5p	RNA	-	10/11	0.005859	2
199	hnRNP F	Generic binding protein	+	10/11	0.005859	3
200	miR-18a-5p	RNA	-	10/11	0.005859	2
201	C10orf90	Generic protein	+	10/11	0.005859	2
202	CTIP2	Transcription factor	-	10/11	0.005859	2
203	AEBP1	Transcription factor	+	16/20	0.005909	3
204	DNAJA3 (TID1)	Regulators (GDI, GAP, GEF etc.)	-	21/28	0.00627	3
205	miR-505-3p	RNA	-	14/17	0.006363	3
206	FKBP8	Generic binding protein	-	14/17	0.006363	3
207	DNMT1	Generic enzyme	-	14/17	0.006363	2
208	RGS6	Regulators (GDI, GAP, GEF etc.)	+	14/17	0.006363	3
209	CGI-72	Generic binding protein	-	14/17	0.006363	3
210	SOCS3	Generic binding protein	-	14/17	0.006363	2
211	ART-27	Generic binding protein	+	12/14	0.00647	2
212	ZBTB2	Transcription factor	-	12/14	0.00647	2
213	URI	Generic binding protein	-	12/14	0.00647	3
214	SMAR1	Generic binding protein	-	12/14	0.00647	2
215	miR-146a-5p	RNA	-	12/14	0.00647	2
216	miR-644a	RNA	-	12/14	0.00647	3
217	GPS1	Generic binding protein	+	12/14	0.00647	3
218	miR-372-3p	RNA	-	19/25	0.007317	3
219	ROR-beta	Transcription factor	+	7/7	0.007813	3

Key Hubs - Causal Reasoning						
#	Network Object	Molecular Function	Object Activity	Correct/All predictions	p-value ▲	Distance
220	alpha-6/beta-1 integrin	Generic receptor	+	7/7	0.007813	3
221	KCTD11	Voltage-gated ion-channel	+	7/7	0.007813	2
222	CRX	Transcription factor	+	7/7	0.007813	3
223	HDAC1	Generic enzyme	-	7/7	0.007813	1
224	SAMSN1	Generic binding protein	-	7/7	0.007813	2
225	BAFF-R	Generic receptor	+	7/7	0.007813	3
226	IL-27 receptor	Generic receptor	+	7/7	0.007813	3
227	microRNA 449a	RNA	+	7/7	0.007813	2
228	ING2	Generic binding protein	-	7/7	0.007813	2
229	IRF9	Transcription factor	+	7/7	0.007813	2
230	miR-483-3p	RNA	-	7/7	0.007813	2
231	JAK1	Protein kinase	+	7/7	0.007813	2
232	CDK5R1 (p25)	Generic binding protein	+	7/7	0.007813	2
233	miR-520h	RNA	+	7/7	0.007813	2
234	GSC	Transcription factor	-	7/7	0.007813	2
235	EVX-1	Transcription factor	+	7/7	0.007813	3
236	NRL	Transcription factor	+	7/7	0.007813	2
237	KCTD21	Voltage-gated ion-channel	+	7/7	0.007813	2
238	MTG16 (CBFA2T3)	Transcription factor	-	7/7	0.007813	2
239	DACT1	Generic binding protein	-	7/7	0.007813	2
240	Ski	Generic binding protein	-	17/22	0.00845	3
241	miR-146a-5p	RNA	-	17/22	0.00845	3
242	HIPK1	Protein kinase	-	20/27	0.009579	3
243	miR-491-3p	RNA	-	20/27	0.009579	3
244	ETV2	Transcription factor	+	20/27	0.009579	3
245	FNDC3B	Generic binding protein	-	15/19	0.009605	3
246	RANBP3L	Generic protein	-	15/19	0.009605	3
247	BMPR1B	Receptor with enzyme activity	+	15/19	0.009605	3
248	TdIF1	Generic binding protein	-	15/19	0.009605	3
249	PSMD10 (Gankyrin)	Generic binding protein	-	16/16	1.526E-05	2
250	PSMD10 (Gankyrin)	Generic binding protein	-	27/31	1.698E-05	3

Key Hubs - Causal Reasoning						
#	Network Object	Molecular Function	Object Activity	Correct/All predictions	p-value ▲	Distance
251	DAXX	Generic binding protein	-	19/20	2.003E-05	2
252	MOZ	Generic enzyme	+	15/15	3.052E-05	2
253	miR-518a-3p	RNA	-	14/14	6.104E-05	3
254	miR-520e	RNA	-	14/14	6.104E-05	2
255	NIK(MAP3K14)	Protein kinase	+	14/14	6.104E-05	2
256	miR-520c-5p	RNA	-	14/14	6.104E-05	2
257	GFI-1	Transcription factor	-	17/18	7.248E-05	2
258	LCMR1	Generic binding protein	-	17/17	7.629E-06	3
259	NRSF	Transcription factor	-	17/17	7.629E-06	2
260	ZFP36L2	Transcription factor	+	17/17	7.629E-06	3
261	IFT57 (HIPPI)	Generic binding protein	-	17/17	7.629E-06	3
262	Peregrin	Generic binding protein	+	17/17	7.629E-06	3
