# Supplementary material for: High expression of ID family and IGJ genes signature as predictor of low induction treatment response and worst survival in adult Hispanic patients with B-acute lymphoblastic leukemia
Source: J Exp Clin Cancer Res. 2016 Apr 5;35:64. doi: 10.1186/s13046-016-0333-z (PMC4820984; doi:10.1186/s13046-016-0333-z)
Supplement: Additional file 6: — Complete file derived from pathway analysis in patients who achieved complete remission. Signaling analysis was done using MetaCore KPA. File shows 4 key pathways dysregulated in this group of patients. (PDF 658 kb) [file 13046_2016_333_MOESM6_ESM.pdf]

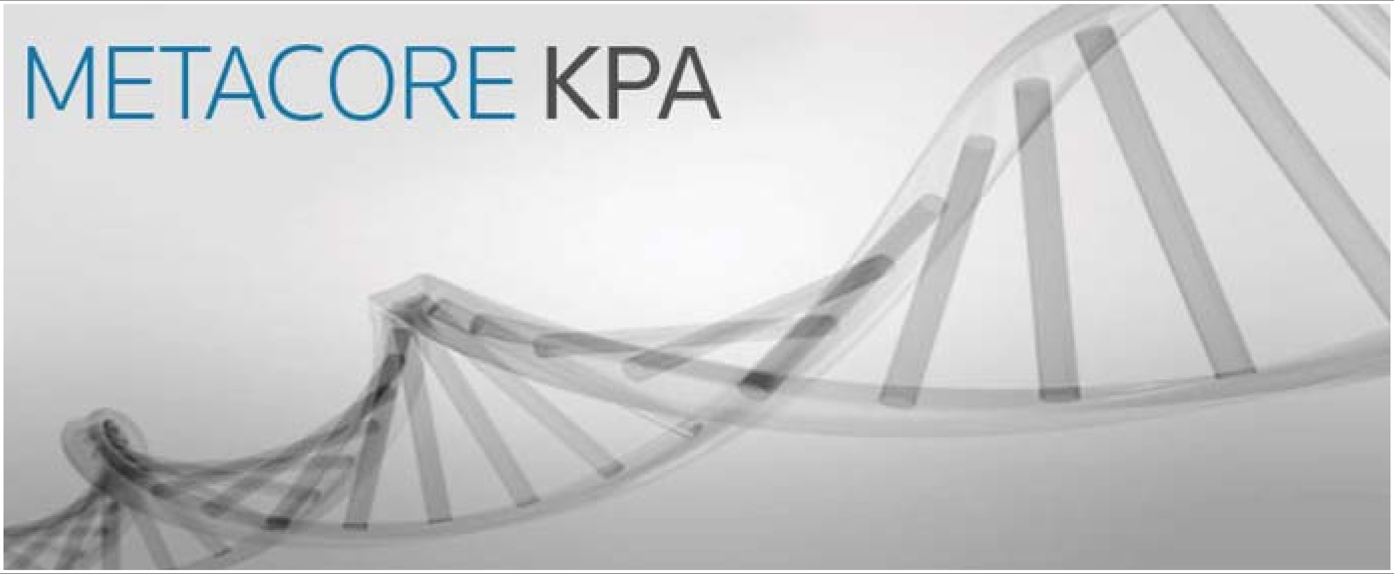

Remision

05 NOV 2015

CONTENTS

INTRODUCTION 3
INPUT DATA AND SETTINGS 4
RESULTS 5
Key Pathway Maps 5
Diseases (by Biomarkers) 15
Process Networks 18
Map Folders 19
Appendix 1: Legend 20
Appendix 2: Glossary 21
Appendix 3: List of Key Hubs IDs 23

INTRODUCTION

In order to understand functional processes behind the differentially expressed gene (DEG) list, KPA represents comprehensive pathway analysis workflow.

The Causal Reasoning approach and Overconnectivity analysis help identify both key direct regulators (i.e. one step away) of the dataset and the major “master” regulators of the global protein network. Concurrent enrichment analysis of both differentially expressed genes and their ranked direct and indirect regulators (Key Hubs), allows one to reconstruct the mechanisms underlining differential gene expression more comprehensively.

We define “Key Ontology Processes” as ontology terms (i.e. pathway maps) enriched with both differentially expressed genes and corresponding Key Hubs. They are identified by the following workflow.

1. Enrichment analysis is performed for the list of differentially expressed genes. Statistically significant ontology processes (enrichment p-value < 0.01) for differentially expressed genes are identified.
2. Calculation of Key Hubs by either Causal Reasoning approach (if DEGs associated with expression values) or Overconnectivity analysis (if DEGs uploaded without expression values).
3. Enrichment analysis is performed for the corresponding list of Key Hubs. Statistically significant ontology processes (enrichment p-value < 0.01) for Key Hubs are identified.
4. Ontology processes statistically significant for both the list of differentially expressed genes and the list of corresponding Key Hubs are identified.
5. Ontology processes that display “synergistic” behaviour for the list of differentially expressed genes and the list of corresponding Key Hubs are defined (please see “Enrichment synergy” in Glossary). The final list of synergistic ontology processes includes all ontology terms with synergistic expression pattern for the union of DEGs and Key Hubs and p-value < 0.01.
6. The resulted list of key processes includes ontology terms which show significant enrichment for both lists and synergistic behaviour.

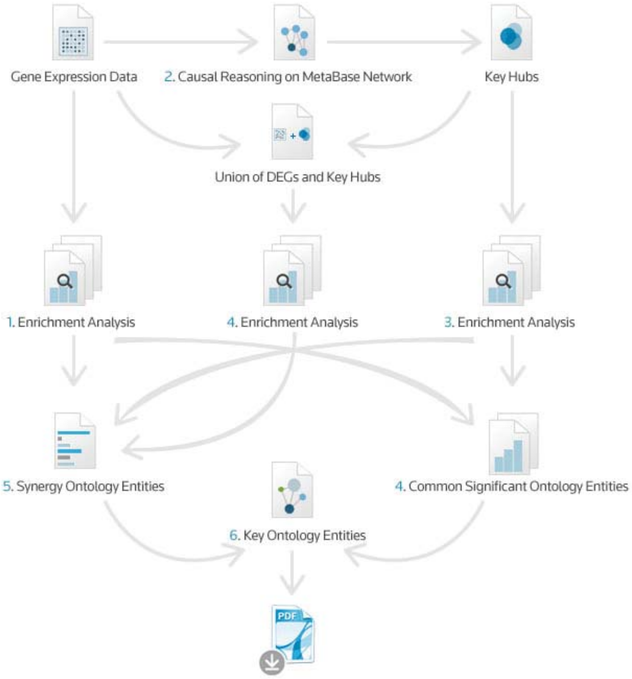

INPUT DATA AND SETTINGS

This section contains experiment file name and data format. Statistics section contains Ids that were in original file and how many of them were recognized by system and mapped on Network Objects. Analysis settings that were used for Key Hubs and Processes calculation steps are listed in the last section.

Analysis Overview	
Analysis Name	Remision
File Type	Gene Expression
File Content	Tag ID: Gene Symbol, Fold change
KPA Version	2.0

Statistics

IDs in File	204
Network objects	229
Unrecognized IDs	23

Analysis Settings

Selected Processes Ontologies	Key Pathway Maps, Diseases (by Biomarkers), Process Networks, Map Folders
Key Processes p-value Threshold	0.01
Key Hubs Calculation Algorithm	Causal Reasoning Analysis
Key Hubs p-value Threshold	0.01

This report contains only top 100 key results for each ontology.

RESULTS

Key Pathway Maps

Pathway maps are graphic images representing complete biochemical pathways or signaling cascades in a commonly accepted sense. All maps listed below are enriched with both input genes and Key Hubs.

Key Pathway Maps Details [4 processes]				
#	Name	Input Objects p-value ▲	Key Hubs p-value	Union Objects p-value
1	Immune response_ETV3 affect on CSF1-promoted macrophage differentiation	1.345E-5	0.001141	9.637E-6
2	Apoptosis and survival_p53-dependent apoptosis	1.52E-4	0.005134	3.349E-6
3	Development_YAP/TAZ-mediated co-regulation of transcription	0.001933	5.886E-5	1.925E-6
4	Transcription_Role of heterochromatin protein 1 (HP1) family in transcriptional silencing	0.00639	3.853E-4	5.152E-6

Maps and Descriptions [1 of 4]

Name	Input Objects ▲ p-value	Key Hubs p-value	Union Objects p-value
Immune response_ETV3 affect on CSF1-promoted macrophage differentiation	1.345E-5	0.001141	9.637E-6

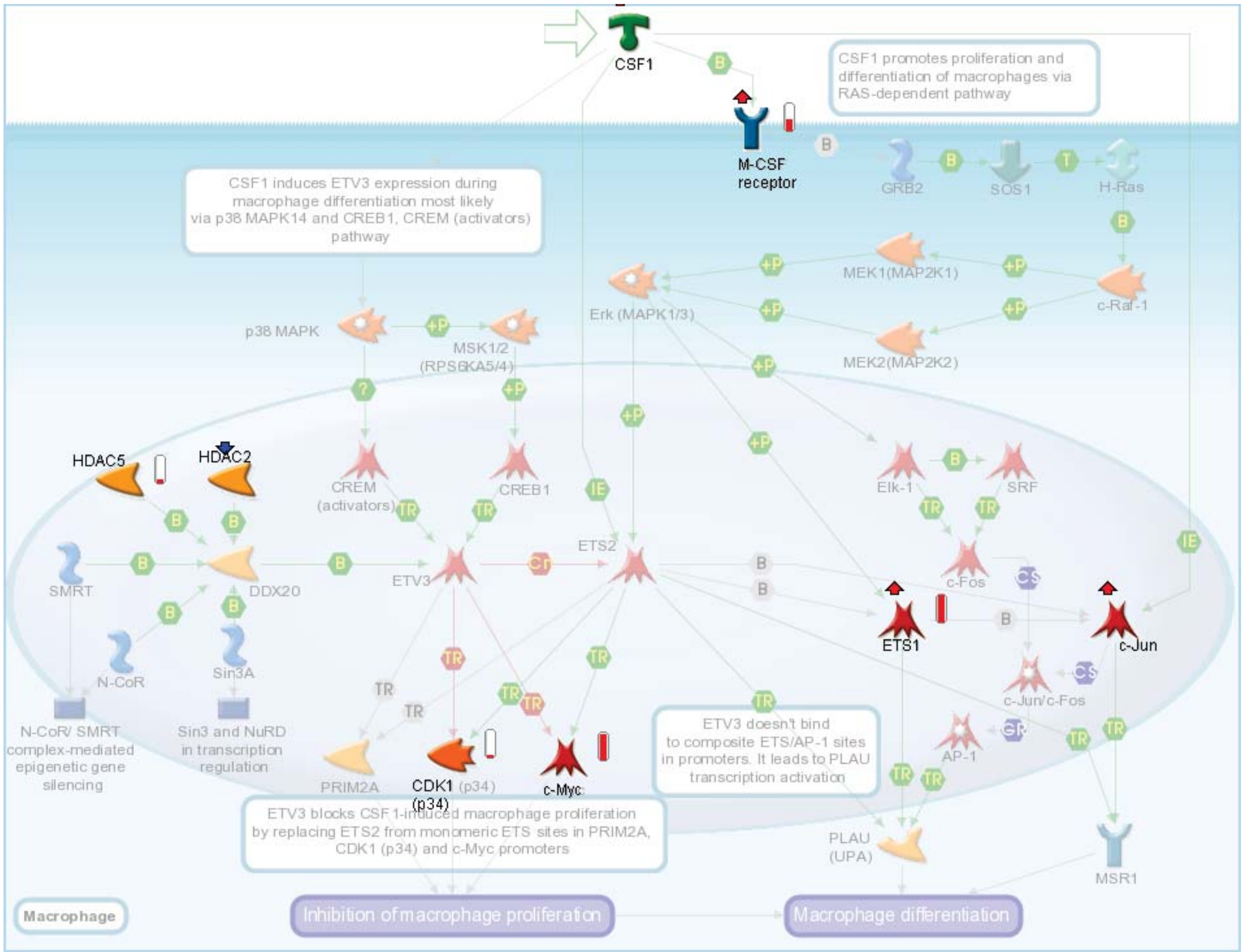

Description

CSF1 is involved in proliferation, differentiation and survival of cells of the monocyte/ macrophage lineage.

CSF1 acts via **M-CSF receptor** and transmits a growth and differentiation signal by activation of RAS signaling pathway [1], [2]. Activated by **CSF1** **M-CSF receptor** binds to **GRB2** coupled with **SOS1**. Activated **SOS1** stimulates **H-Ras** and it leads to **ERK1/2** activation through **H-Ras**/ **c-Raf-1**/ **MEK1(MAP2K1), MEK2(MAP2K2)** pathway [3], [4]. Activated **ERK1/2** phosphorylates**ETS1** and **ETS2** [5], [6]. Also, **ERK1/2** activation by **CSF1** leads to **Elk-1** phosphorylation, **Elk-1** binds to **SRF** and stimulates **c-Fos** transcription [3], [5], [7], [8]. In addition, **CSF1** stimulation enhances expression of **ETS2** and **c-Jun** [9], [10]. **ETS1**, **ETS2**, **c-Jun** and **c-Fos** forms ternary complexes and bind to composite ETS/**AP-1** sites in **PLAU (UPA)** [11], [12] and **MSR1** [13], [14] promoters and thus promote macrophage differentiation [10], [15].

Proliferation signaling of **CSF1** also involves RAS pathway and leads to **ETS2** phosphorylation by **ERK1/2. ETS2** binds to monomeric sites in **c-Myc**, **CDK1 (p34)** and **PRIM2A** promoters and thus promoter **CSF1** induced proliferation [4], [10].

ETV3 of the ETS-domain family transcription factors that functions as a transcriptional repressor can block mitogenic responses mediated by positively acting Ets factors. **ETV3** itself is induced by **CSF1,** most likely via **CSF1** promoted **p38 MAPK** activation pathway during macrophage differentiation [16]. **p38 MAPK** stimulates phosphorylation of **CREM (activators)** and **CREB1**, probably via **MSK1/2 (RPS6KA5/4)** [17]. **CREM (activators)** and **CREB1** in turn, activate transcription of **ETV3** [18]. Repressor activity of **ETV3** is stimulated by binding of **DDX20** associated with **HDAC2**, **HDAC5**, **N-CoR**, **SMRT** and **Sin3A** [19]. **ETV3** can bind only to monomeric ETS sites in **PRIM2A**, **CDK1 (p34)** and **c-Myc** promoters. Thus, it replaces **ETS2** and represses promoter activity of proliferative genes. However, it is not able to bind to ETS/AP-1 composite sites, so promoters of differentiation-responsive genes remain unaffected by **ETV3** and **CSF1**-induced cell differentiation goes on and cell proliferation is blocked [10].

Maps and Descriptions [2 of 4]

Name	Input Objects ▲ p-value	Key Hubs p-value	Union Objects p-value
Apoptosis and survival_p53-dependent apoptosis	1.52E-4	0.005134	3.349E-6

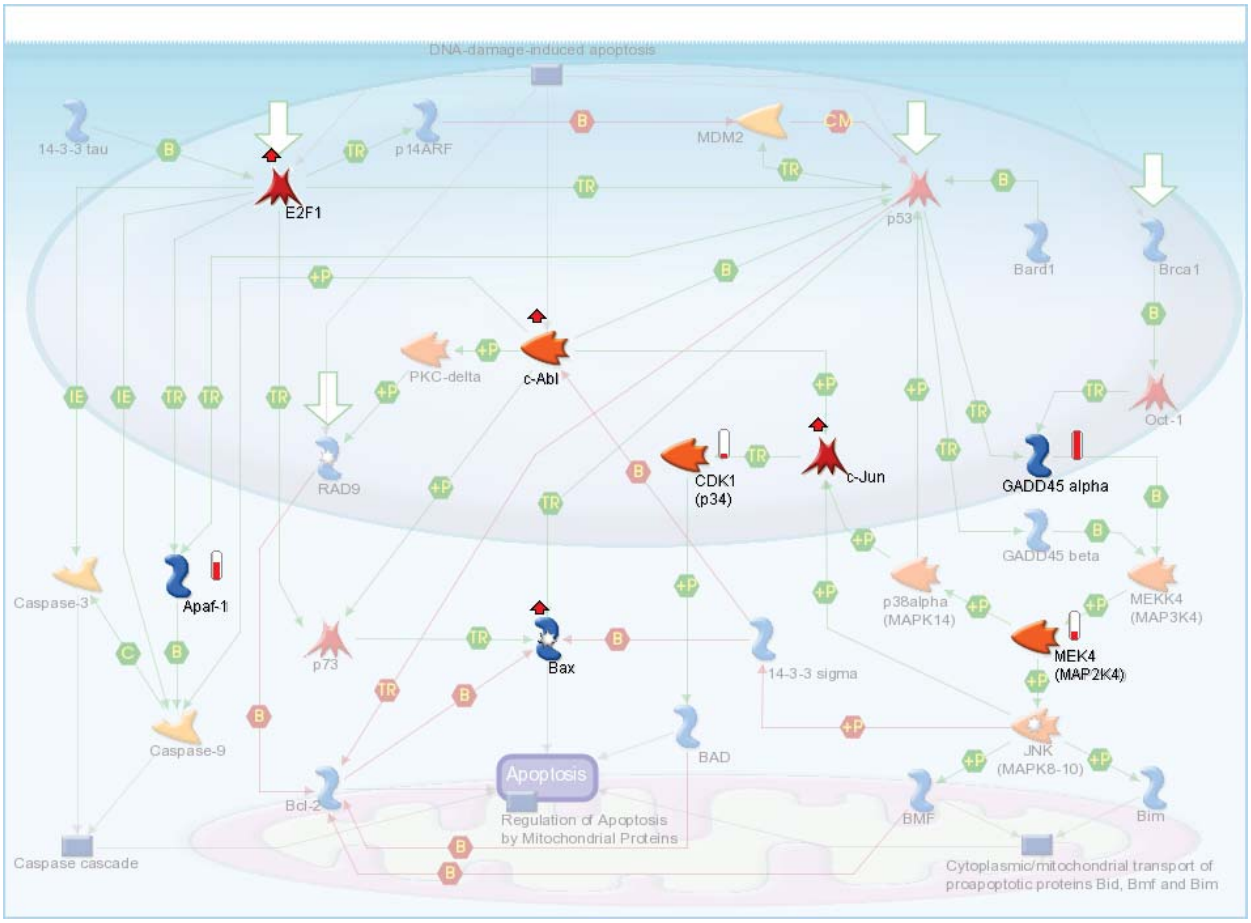

Description

p53-dependent apoptosis

Direct DNA damage by ionizing radiation or UV can activates the interconnected apoptotic pathways by stimulation of protein kinases from phosphoinositide-3-kinases family. These stimulated kinases directly or indirectly activate phosphorylation of proteins from apoptotic pathways: tumor suppressor **p53**, breast and ovarian cancer susceptibility protein 1 (**Brca1**), E2F transcription factor 1 (**E2F1**), proto-oncogene tyrosine-protein kinase **c-Abl** and cell cycle regulator **RAD9**. [1], [2]

Stimulated **Brca1** and **p53** excite transcription of growth arrest and DNA-damage-inducible transcripts alpha and beta (**GADD45 alpha/beta**)**. GADD45alpha/beta** triggers apoptosis through activation of mitogen-activated protein kinase kinase kinase 4 (**MEKK4**)/ mitogen-activated protein kinase kinase 4 (**MKK4**) or mitogen-activated protein kinase kinase 7 (**MKK7**)/ c-Jun N-terminal kinase/stress-activated protein kinase (**JNK**) cascade and/or **MEKK4** / **MKK4** or **MKK7** / mitogen-activated protein kinase 14 (**p38 alpha**) cascade. [1], [3], [4]

JNK and **p38 alpha** activate transcription of **c-Jun**, which, in turn, excites transcription of cyclin-dependent kinase 1 (**CDK1**). [5] **CDK1** catalyzes phosphorylation of the Bcl-2 - antagonist of cell death protein(**BAD**) at a distinct site, serine 128, and thereby induces **BAD**-mediated apoptosis. [6] Moreover, **JNK** stimulates activity of proapoptotic proteins, Bcl-2 modifying factor (**BMF**) and BCL2-like 11 factor (**Bim**). [7]

JNK phosphorylates 14-3-3protein, type sigma(**14-3-3 sigma**) and stimulates the break of the links between **14-3-3 sigma** and **c-Alb** and between **14-3-3 sigma** and Bcl-2 - associated X protein (**Bax**),that result in activation **c-Alb** and **Bax**.Activated **c-Alb** participates in apoptotic pathways (e.g. via phosphorylation tumor p53-related protein (**p73**) [8], transcription factor**c-Jun** or apoptosis-related cysteine protease 9 (**Caspase-9**). [9]

In addition, **c-Alb** activatesprotein kinase C, delta (**PKC delta**). [10] **PKC- delta** and **ATM** may activate by phosphorylation **RAD9**. **RAD9**, **BAD** and **BMF** are bound withB-cell lymphoma protein 2 (**Bcl-2**) and inhibit its anti-apoptosis activity.

Nuclear protein **p53** plays an important role in apoptosis. The basal **p53**-**Mdm2** loop is an essential component of **p53** regulation is. The ubiquitin-protein ligase E3 **Mdm2** protein ubiquitinates and promotes degradation of **p53**. On the other hand, the **mdm2** gene is the direct target for binding and transcriptional activation by **p53**. [11] **c-Abl** neutralizes the inhibitory effect of **Mdm2** on **p53**. [12], [13] In addition, during indirect activation of **p53**, **E2F1** excite the transcription of cyclin-dependent kinase inhibitor 2A (**p14ARF**), which interacts with **Mdm2** and blocks the ability of **Mdm2** to target **p53** for destruction. [14] **p53** may be activated by component of **Brca1**/**BARD1** complex, **BARD1** too. [15]

Activated **p53** regulates transcription of apoptosis regulator **Bax** [16], apoptotic protease activating factor (**Apaf-1**) [17] and **Bcl-2**. [18]

E2F may activate apoptosis pathways by stimulation **of p53** 14-3-3protein, type tau (**14-3-3 tau**) is required for expression and induction of **E2F1** apoptotic targets, such as **p73**, **Apaf-1**, and apoptosis-related cysteine protease 3 (**Caspase-3**). {PMID: 15494392}

Maps and Descriptions [3 of 4]

Name	Input Objects ▲<br>p-value	Key Hubs<br>p-value	Union Objects<br>p-value
Development_YAP/TAZ-mediated co-regulation of transcription	0.001933	5.886E-5	1.925E-6

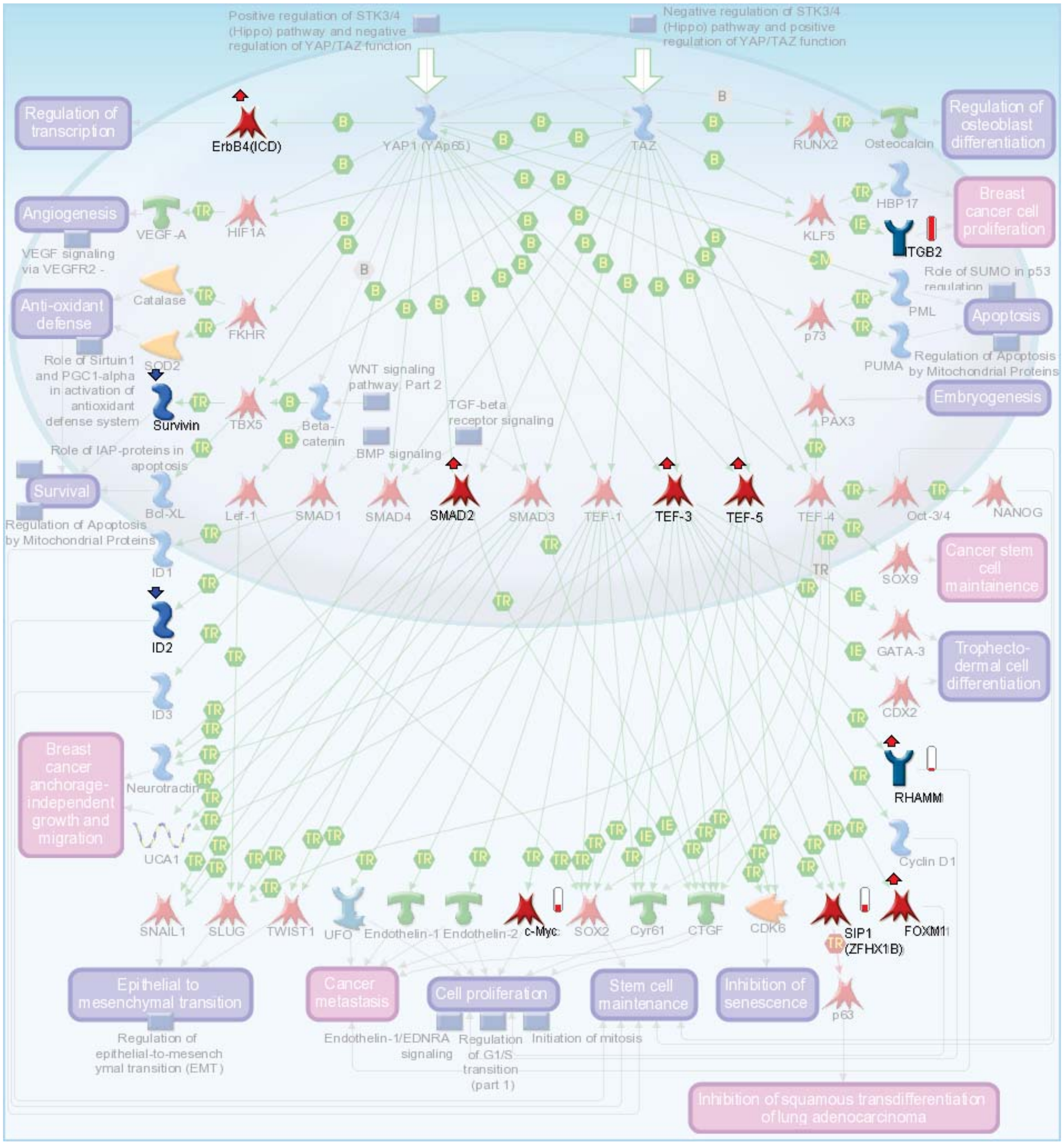

Abstract:

YAP1 (YAp65) (YAP) and **TAZ** are co-regulators of transcription that are well-known targets of so-called Hippo pathway. Activities of **YAP1 (YAp65)** and **TAZ** are regulated by phosphorylation. In general, phosphorylated **YAP1 (YAp65)** and **TAZ** localized to the cytoplasm and unable to regulate transcription, while unphosphorylated proteins localized in the nucleus where they regulate activity of transcription factors. **YAP1 (YAp65)** and **TAZ** activate multiple transcription factors such as transcriptional enhancer factor (TEF) family **TEF-1**, **TEF-3**, **TEF-4** and **TEF-5**, SMAD family, **RUNX2**, **KLF5**, **p73** etc. thus regulating fundamental normal cellular processes such as cell proliferation, cell survival, cell differentiation, stem cell self-renewal, embryogenesis etc.

Details:

YAP1 (YAp65) and **TAZ** are co-regulators of transcription that are well-known targets of so-called Hippo pathway. **YAP1 (YAp65)** and **TAZ** via binding to multiple transcriptional factors regulate fundamental normal cellular processes such as cell proliferation, cell survival (see negative regulation of apoptotic process), cell differentiation, stem cell self-renewal (see stem cell maintenance), embryogenesis (see embryo development) etc. Moreover, **YAP1 (YAp65)** and **TAZ** function dysregulation is involved in pathological processes such as human cancer development [1], [2], [3], [4], [5], [6].

Activities of **YAP1 (YAp65)** and **TAZ** are regulated by phosphorylation. In general, phosphorylated **YAP1 (YAp65)** and **TAZ** localized to the cytoplasm and unable to regulate transcription, while unphosphorylated proteins localized in the nucleus where they regulate activity of transcription factors [1], [2], [3], [4], [5], [6]. However, there is at least one exception: phosphorylated form of **YAP1 (YAp65)** is involved in regulation of **p73**-mediated transcription [7], [8].

YAP1 (YAp65) binds to **ErbB4(ICD)** and increases its transcriptional activity [9], [10].

Under hypoxic condition, **YAP1 (YAp65)** and **TAZ** bind to and stabilize **HIF1A** thus inducing transcription of downstream **HIF1A** target genes such as pro-angiogenic **VEGF-A** (see angiogenesis) [11], [12].

YAP1 (YAp65) interacts with **FKHR** forming a functional complex on the promoters of **Catalase** and **SOD2** antioxidant genes which stimulates their transcription leading to anti-oxidant defense and survival (see negative regulation of apoptotic process) [13].

TAZ directly associates with **TBX5** and markedly stimulates **TBX5**-dependent promoters. **YAP1 (YAp65)** also stimulates **TBX5**-dependent transcription, possibly by forming a heterodimer with **TAZ** [14]. Moreover, while phosphorylated **YAP1 (YAp65)** acts as inhibitor of **Beta-catenin** co-transcriptional activity [15], unphosphorylated **YAP1 (YAp65)** interacts with **Beta-catenin** in the nucleus [16], and **YAP1 (YAp65)** co-operates with **Beta-catenin** to activate **TBX5**-mediated transcription of anti-apoptotic proteins **Survivin** and **Bcl-XL** (see negative regulation of apoptotic process) [17].

YAP1 (YAp65) binds to **SMAD1**, activated downstream of **BMP** signaling, thus inducing expression of **ID1**, **ID2** and **ID3** which inhibit neural differentiation of embryonic stem cells thus promoting stem cell maintenance [16], [18].

Moreover, **YAP1 (YAp65)** and **TAZ** bind to and activate **SMAD4**, **SMAD2** and **SMAD3** supporting **TGF-beta**-triggered **SMAD2/3/4**-mediated transcription [16], [19], [20]. Activated **SMAD2/3/4** induces **SNAIL1**, **SLUG** and **TWIST1** transcription factors thus contributing to epithelial to mesenchymal transition [20].

Moreover, the key targets of **YAP1 (YAp65)** and **TAZ** are transcriptional enhancer factor (TEF) family **TEF-1**, **TEF-3**, **TEF-4** and **TEF-5** [21], [22], [2]. **TEF-1/3/4/5** activates transcription and induces expression of **CTGF** [23], [24], [25] and **Cyr61** [26] thus contributing to cell proliferation. Activated **SMAD2/3** also induces **CTGF** expression contributing to cell proliferation and cancer metastasis [6], [25].

In addition, **TEF-1/3/5** induces **CDK6** expression thus inhibiting cellular senescence [27].

Moreover, **TEF-1** induces expression of **UFO** [28], **Endothelin-1**, **Endothelin-2** and **c-Myc** [29] to promote cell proliferation and cancer cell metastasis [6].

TEF-1/3/5 also contributes to stem cell maintenance via induction of **SOX2** [30]. Moreover, **YAP1 (YAp65)**-activated **TEF-4** and **Beta-**

catenin-activated Lef-1 co-operatively induce SLUG and SOX2 expression [31].

Both TAZ-activated TEF-3 and **SMAD2/3** activates transcription of Neurotractin and UCA1 which are necessary for anchorage-independent growth and migratory properties of breast cancer cells [25].

In addition, **TEF-1/3/5** induces SIP1 (ZFHX1B) transcription which in turn represses p63 transcription thus leading to inhibition of squamous transdifferentiation of lung adenocarcinoma [32].

YAP1 (YAp65)-activated TEF-3 induces expression of Cyclin D1 and FOXM1 which contributes to cell proliferation [33].

In addition, TEF-3 activates transcription of RHAMM promoting cancer cell migration and invasion (metastasis) [34].

Moreover, TEF-3 induces expression of GATA-3 [35] and CDX2 [36], [37] thus leading to trophectodermal cell differentiation and development.

TEF-4 activates PAX3 transcription [38], and, additionally, TAZ enhances PAX3 transcriptional activity thus contributing to embryogenesis (see embryo development) [39]. In addition, TEF-4 activates transcription of Oct-3/4 which in turn induces NANOG expression leading to stem cell maintenance [40]. Moreover, TEF-4 induces SOX9 thus contributing to cancer stem cell maintenance [41].

Phosphorylated YAP1 (YAp65) binds to p73 forming a functional complex in the nucleus which activates transcription of the pro-apoptotic PUMA gene [7], [8]. Moreover, YAP1 (YAp65)/ p73 complex also induces transcription of another pro-apoptotic gene PML, which in turn can exert positive feedback loop via binding to YAP1 (YAp65) and increasing its stability [42], [43].

Moreover, YAP1 (YAp65) [44] and TAZ [45] bind to and stabilize KLF5 which leads to induction of KLF5 downstream targets HBP17 and ITGB2 thus contributing to breast cell proliferation [44].

Finally, YAP1 (YAp65) regulates RUNX2 activity thus changing expression of Osteocalcin, although data about YAP1 (YAp65) effect are contradicting [46], [47]. However, TAZ seems to be co-activator of RUNX2 contributing to Osteocalcin gene expression and thus to osteoblast differentiation [48].

Maps and Descriptions [4 of 4]

Name	Input Objects ▲ p-value	Key Hubs p-value	Union Objects p-value
Transcription_Role of heterochromatin protein 1 (HP1) family in transcriptional silencing	0.00639	3.853E-4	5.152E-6

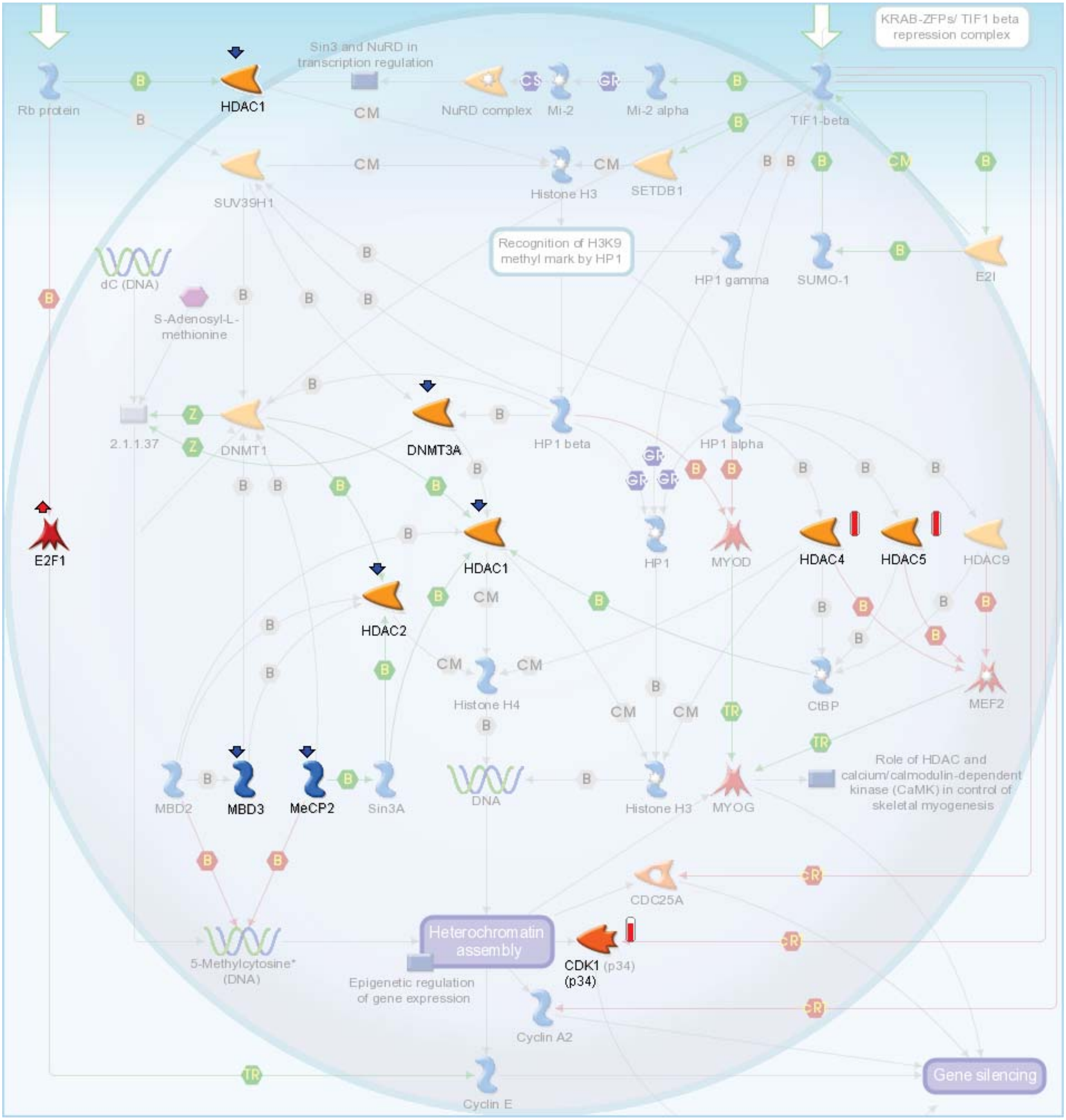

Abstract:

HP1 proteins, recognized readers of the heterochromatin mark methylation of **Histone H3** at K9 (H3K9), are important regulators of heterochromatin-mediated gene silencing and chromosome structure. **HP1** proteins cooperate with DNA and histone methyltransferases, methyl CpG binding domain-containing proteins, histone deacetylases and transcriptional corepressors to achieve an effective gene silencing.

Details:

HP1 proteins, recognized readers of the heterochromatin mark methylation of **Histone H3** at K9 (H3K9), are important regulators of heterochromatin-mediated gene silencing and chromosome structure [1], [2], [3], [4], [5]. In mammals, there are three **HP1** proteins, **HP1 alpha**, **HP1 beta** and **HP1 gamma**, that differ from each other by their spatial distribution within the nucleus, **HP1 alpha** being almost exclusively located in heterochromatin, whereas **HP1 beta** and even more extensively **HP1 gamma** are also found in euchromatin [6], [7].
HP1 beta recruits the H3K9 methyltransferase **Suv39H1** to chromatin, leading to spreading of H3K9 methylation along the chromatin fiber [3], [8], [9]. Additionally, **HP1 beta** and **Suv39H1** associates with both **DNMT1** and **DNMT3A** DNA methyltransferases [10]. In turn, **DNMT1** and **DNMT3A** cooperates with **HDAC1** and **HDAC2** [11], [12], [13], [14], [15] to bring about partial transcriptional repression [16], [17]. The generation of methylated chromatin by the DNA methyltransferases would allow DNA binding of methyl CpG binding domain (MBD)-containing proteins. **MBD2**, **MBD3** and **MeCP2** interact directly with **DNMT1** [12], [18], [19] and further recruit HDAC activities, thereby, lock chromatin into a fully silenced state [16]. Also, **MeCP2** represses gene expression by recruiting **Sin3A**, which interacts with **HDAC1** and **HDAC2** [20], [21]. **MBD2** associates directly with **HDAC1** and **HDAC2** [22], whereas **MBD3** interacts only with **HDAC2** [23].
HP1 alpha associates with class II HDACs, such as **HDAC4**, **HDAC5** and **HDAC9**, and **Suv39H1** to form a multiprotein transcriptional repression complex [24], [25]. In turn, class II HDACs bind to and inhibit **MEF2** transcriptional activity [26], [27], [28], [29], [30], [31] via targeting **HP1 alpha**/ class II HDACs/ **Suv39H1** repressive complex to **MEF2**-binding site in muscle-specific genes, such as **MYOG** [32], with subsequent gene silencing [24], [33]. Also, class II HDACs interact with the transcriptional corepressor **CtBP**, which associates with class I HDACs [34] and with other corepressors, such as N-CoR and SMRT, leading to repression of **MEF2**-dependent transcription [35]. Moreover, **HP1 alpha** and **HP1 beta** bind directly and inhibit **MYOD** transcriptional activity [36].

In addition to heterochromatic silencing, **Suv39H1** and **HP1** are involved in repression of euchromatic genes [37]. **Suv39H1** cooperates with **Rb protein** to repress the **Cyclin E** promoter [38]. **Rb protein** bound to **E2F1** recruits **Suv39H1** to the S-phase-specific gene promoter (e.g., **Cyclin E**) to methylate Lys 9 of **Histone H3** and provide a binding site for **HP1** [38], [39], [40]. **HP1** may protect the methyl group on Lys 9 from attack from potential demethylases, it may bring in other repressive functions, or it may enhance the stability of the **Rb protein**-associated repressor complex [38]. Notably, deacetylation of histone H3 at Lys 9 by **Rb protein**-associated deacetylase activity might be required as a preceding step to **Suv39H1**-mediated methylation [41], [42], [43].
HP1 alpha, **HP1 beta** and **HP1 gamma** interact with **TIF1-beta**, co-repressor for the KRAB zinc finger protein superfamily of transcriptional factors (KRAB-ZFPs), and facilitate **TIF1-beta**-mediated transcriptional repression [6], [7], [44], [45]. KRAB-ZFPs bind to its cognate recognition sequence and then recruits **TIF1-beta** to form a scaffold that coordinates the assembly histone deacetylases, histone methylases and the deposition of **HP1** proteins to silence gene expression by forming a facultative heterochromatin environment [44], [46], [47]. **TIF1-beta** binds to **SETDB1**, resulting in histone H3-K9 methylation and enhancement the binding of **HP1** proteins [46]. Also, **TIF1-beta** is associated with **Mi-2 alpha**, an integral component of the **NuRD complex** [48]. **NuRD complex** deacetylates histones in the promoter region, creating a favorable condition for **HP1** proteins to nucleate a local heterochromatin environment that results in effective gene silencing [48]. Moreover, sumoylation is required for KRAB-ZFPs/ **TIF1-beta**-mediated gene repression. **TIF1-beta** PHD domain binds to **E2I** and directs **SUMO-1** conjugation of an adjacent bromodomain. The PHD domain of **TIF1-beta** functions as an intramolecular SUMO E3 ligase. **SETDB1** and **Mi-2 alpha** encode functional SIM motifs and bind SUMO-modified **TIF1-beta** [49]. Notably, **HP1**/ **TIF1-beta** complex is associated with the promoter regions of **Cyclin A2**, **CDK1 (p34)** and **CDC25A**, causing repression of these genes [50].

Diseases (by Biomarkers)

This ontology is created based on the classification in Medical Subject Headings (MeSH). Each disease in diseases ontology has its corresponding biomarker gene or set of genes annotated manually from the literature.

Diseases (by Biomarkers) Details [Top 100 processes]					
#	Name	Input Objects ▲ p-value	Key Hubs p-value	Union Objects p-value
1	Stomach Diseases	7.615E-11	1.943E-23	6.263E-32
2	Stomach Neoplasms	1.116E-10	1.195E-23	5.744E-32
3	Breast Neoplasms	2.283E-9	4.005E-18	2.877E-25
4	Breast Diseases	2.302E-9	4.081E-18	2.955E-25
5	Immune System Diseases	1.401E-8	4.943E-29	9.087E-35
6	Diabetes Mellitus	3.341E-8	1.9E-7	3.625E-13
7	Diabetes Mellitus, Type 2	4.797E-8	3.603E-4	9.783E-10
8	Gonadal Disorders	5.842E-8	4.851E-21	1.827E-26
9	Ovarian Neoplasms	8.495E-8	1.124E-21	7.312E-27
10	Endocrine System Diseases	9.861E-8	3.314E-23	2.888E-28
11	Ovarian Diseases	1.37E-7	7.176E-21	6.91E-26
12	Adnexal Diseases	1.418E-7	7.897E-21	7.851E-26
13	Pathological Conditions, Signs and Symptoms	2.598E-7	1.008E-17	9.511E-23
14	Hemic and Lymphatic Diseases	2.882E-7	1.117E-20	1.415E-25
15	Skin and Connective Tissue Diseases	5.062E-7	1.298E-12	1.193E-17
16	Liver Neoplasms	5.268E-7	3.068E-23	3.669E-27
17	Carcinoma, Hepatocellular	5.576E-7	1.598E-22	1.892E-26
18	Wounds and Injuries	1.128E-6	3.999E-12	3.191E-17
19	Glucose Metabolism Disorders	1.288E-6	6.697E-6	3.451E-10
20	Skin Diseases	1.314E-6	2.057E-12	4.947E-17
21	Nutritional and Metabolic Diseases	3.024E-6	6.971E-7	3.822E-11
22	Genital Neoplasms, Female	3.113E-6	0.001737	7.337E-7
23	Signs and Symptoms	5.49E-6	1.202E-5	4.654E-10
24	Gastrointestinal Neoplasms	5.788E-6	1.976E-14	2.399E-18
25	Metabolic Diseases	5.86E-6	2.993E-7	2.762E-11
26	Female Urogenital Diseases and Pregnancy Complications	6.23E-6	8.193E-6	1.231E-9
27	Genital Diseases, Female	6.297E-6	4.395E-4	1.871E-7
28	RNA Virus Infections	7.784E-6	2.652E-15	1.468E-19
29	Endocrine Gland Neoplasms	8.081E-6	4.869E-25	1.541E-27
30	Female Urogenital Diseases	8.818E-6	6.323E-6	1.172E-9
31	Thyroid Neoplasms	9.324E-6	2.212E-16	4.529E-21
32	Liver Diseases	9.428E-6	5.981E-28	5.483E-30
33	Colonic Diseases	9.943E-6	2.148E-28	2.183E-30

Diseases (by Biomarkers) Details [Top 100 processes]				
#	Name	Input Objects ▲ p-value	Key Hubs p-value	Union Objects p-value
34	Leukemia	1.062E-5	5.119E-11	1.054E-14
35	Sexually Transmitted Diseases	1.193E-5	2.987E-15	2.924E-19
36	Sexually Transmitted Diseases, Viral	1.193E-5	2.987E-15	2.924E-19
37	Connective Tissue Diseases	1.25E-5	1.264E-22	5.39E-26
38	Lentivirus Infections	1.267E-5	3.553E-15	3.695E-19
39	HIV Infections	1.267E-5	3.553E-15	3.695E-19
40	Retroviridae Infections	1.426E-5	1.311E-15	1.584E-19
41	Autoimmune Diseases	1.505E-5	5.345E-25	3.676E-28
42	Bacterial Infections and Mycoses	1.793E-5	4.764E-13	5.536E-17
43	Pathologic Processes	1.853E-5	1.094E-27	1.494E-29
44	Gastrointestinal Diseases	2.208E-5	6.266E-17	4.518E-20
45	Lupus Nephritis	3.872E-5	8.879E-6	1.408E-9
46	Adenocarcinoma	3.946E-5	9.566E-33	2.009E-33
47	Pulmonary Disease, Chronic Obstructive	4.206E-5	7.035E-14	2.43E-17
48	Ischemia	4.395E-5	1.043E-6	5.757E-10
49	Carcinoma, Ductal	5.677E-5	1.512E-24	2.436E-27
50	Immunoproliferative Disorders	5.679E-5	4.285E-19	1.118E-21
51	Carcinoma	5.778E-5	7.035E-35	4.924E-35
52	Immunologic Deficiency Syndromes	6.354E-5	1.28E-16	8.802E-20
53	Rectal Diseases	6.607E-5	4.649E-10	6.186E-13
54	Cerebrovascular Disorders	7.073E-5	2.9E-5	9.765E-9
55	Thyroid Diseases	7.09E-5	3.638E-17	8.579E-21
56	Lymphoproliferative Disorders	8.753E-5	5.229E-20	2.798E-22
57	Neoplasms, Ductal, Lobular, and Medullary	9.151E-5	8.702E-25	2.814E-27
58	Lung Diseases, Obstructive	9.56E-5	5.359E-17	4.057E-19
59	Neoplasms, Neuroepithelial	9.807E-5	4.43E-15	3.541E-17
60	Colorectal Neoplasms	9.825E-5	5.713E-12	1.303E-14
61	Anemia	1.002E-4	6.912E-4	1.32E-6
62	Intestinal Neoplasms	1.053E-4	6.911E-12	1.679E-14
63	Virus Diseases	1.1E-4	2.691E-27	6.405E-29
64	Carcinoma, Ductal, Breast	1.103E-4	7.218E-22	5.418E-25
65	Brain Ischemia	1.3E-4	1.602E-5	7.201E-9
66	Thyroid Carcinoma, Papillary	1.378E-4	4.123E-12	1.373E-15
67	Lymphoma, Non-Hodgkin	1.397E-4	1.413E-12	5.26E-16
68	Neoplasms, Connective Tissue	1.439E-4	1.927E-17	5.238E-19
69	Urogenital Neoplasms	1.919E-4	5.728E-5	1.227E-7
70	Lymphoma	2.1E-4	6.307E-17	5.509E-20

#	Name	Input Objects ▲ p-value	Key Hubs p-value	Union Objects p-value
	Diseases (by Biomarkers) Details [Top 100 processes]			
71	Rheumatic Diseases	2.15E-4	5.111E-15	2.848E-17
72	Glioma	2.306E-4	2.862E-13	4.62E-15
73	Lupus Erythematosus, Systemic	2.446E-4	1.528E-21	3.637E-23
74	Hematologic Diseases	2.598E-4	1.494E-24	3.498E-25
75	Pancreatic Neoplasms	2.929E-4	3.653E-20	9.206E-21
76	Gram-Positive Bacterial Infections	3.029E-4	0.003721	2.255E-5
77	Digestive System Neoplasms	3.18E-4	2.948E-16	4.544E-18
78	Leukemia, Promyelocytic, Acute	3.786E-4	7.624E-7	4.038E-9
79	Anoxia	3.842E-4	7.251E-10	7.418E-13
80	Vascular Diseases	4.291E-4	2.384E-20	1.386E-21
81	Actinomycetales Infections	4.879E-4	0.003077	2.639E-5
82	Neoplasms, Adipose Tissue	4.935E-4	0.004856	1.124E-5
83	Intestinal Diseases	5.093E-4	2.423E-14	5.525E-16
84	Bacterial Infections	5.137E-4	8.648E-5	4.681E-7
85	Neoplasms, Vascular Tissue	5.528E-4	1.595E-10	4.198E-12
86	Lymphatic Diseases	5.563E-4	6.694E-14	1.386E-16
87	Lymphoma, Mantle-Cell	6.497E-4	2.109E-4	5.029E-7
88	Carcinoma, Pancreatic Ductal	6.544E-4	1.87E-11	1.345E-13
89	Arthritis, Rheumatoid	6.938E-4	4.577E-16	1.37E-17
90	Neoplasms, Connective and Soft Tissue	7.0E-4	1.96E-19	4.337E-20
91	Leukemia, Myeloid	7.848E-4	9.684E-17	1.132E-17
92	Infection	9.033E-4	8.474E-13	2.942E-15
93	Nervous System Neoplasms	9.794E-4	3.073E-11	1.373E-12
94	Arthritis	0.001129	5.529E-15	2.314E-16
95	Liver Cirrhosis	0.001259	1.89E-7	6.698E-10
96	Cardiovascular Diseases	0.00133	4.173E-20	1.079E-20
97	Melanoma, Cutaneous Malignant	0.001373	9.153E-16	3.113E-16
98	Leukemia, Myeloid, Acute	0.001406	5.697E-15	3.812E-16
99	Esophageal Diseases	0.001419	7.126E-7	8.396E-9
100	Signs and Symptoms, Respiratory	0.001419	7.813E-10	3.42E-12

Process Networks

A recognized series of events (interactions or biochemical reactions) accomplished by one or more ordered assemblies of molecular functions with a defined beginning and end.

Process Networks Details [2 processes]				
#	Name	Input Objects ▲ p-value	Key Hubs p-value	Union Objects p-value
1	Cell cycle_G2-M	4.904E-4	8.912E-4	2.032E-6
2	Cell cycle_Mitosis	0.003067	4.313E-6	2.635E-8

Map Folders

This is a collection of manually created pathway maps, grouped hierarchically into folders according to main biological processes. A map could participate in different folders if depicted pathway takes part in different main biological processes (Folders).

	Map Folders Details [15 processes]			
#	Name	Input Objects ▲ p-value	Key Hubs p-value	Union Objects p-value
1	Lung cancer	2.524E-7	3.836E-19	1.155E-24
2	Melanoma	1.156E-5	4.172E-17	3.232E-21
3	Asthma	1.97E-5	9.867E-17	8.907E-21
4	Colorectal Neoplasms	2.771E-5	9.115E-22	3.288E-25
5	Cell cycle and its regulation	3.38E-5	6.298E-12	2.983E-15
6	Neurofibromatoses	8.318E-5	6.629E-21	1.268E-22
7	Ovarian cancer	1.372E-4	4.771E-19	8.522E-22
8	Pancreatic Neoplasms	1.671E-4	3.701E-11	4.554E-14
9	Carcinoma, Hepatocellular	3.037E-4	3.631E-22	1.679E-23
10	Prostatic Neoplasms	3.956E-4	8.65E-16	5.359E-18
11	Depression	0.004199	5.572E-7	1.789E-8
12	Stomach Neoplasms	0.004782	4.806E-17	1.028E-17
13	Breast Neoplasms	0.005021	5.542E-14	1.157E-14
14	Apoptosis	0.005502	1.229E-12	3.223E-13
15	Neurodegeneration in Multiple sclerosis	0.009333	4.917E-18	3.038E-18

Appendix 1: Legend

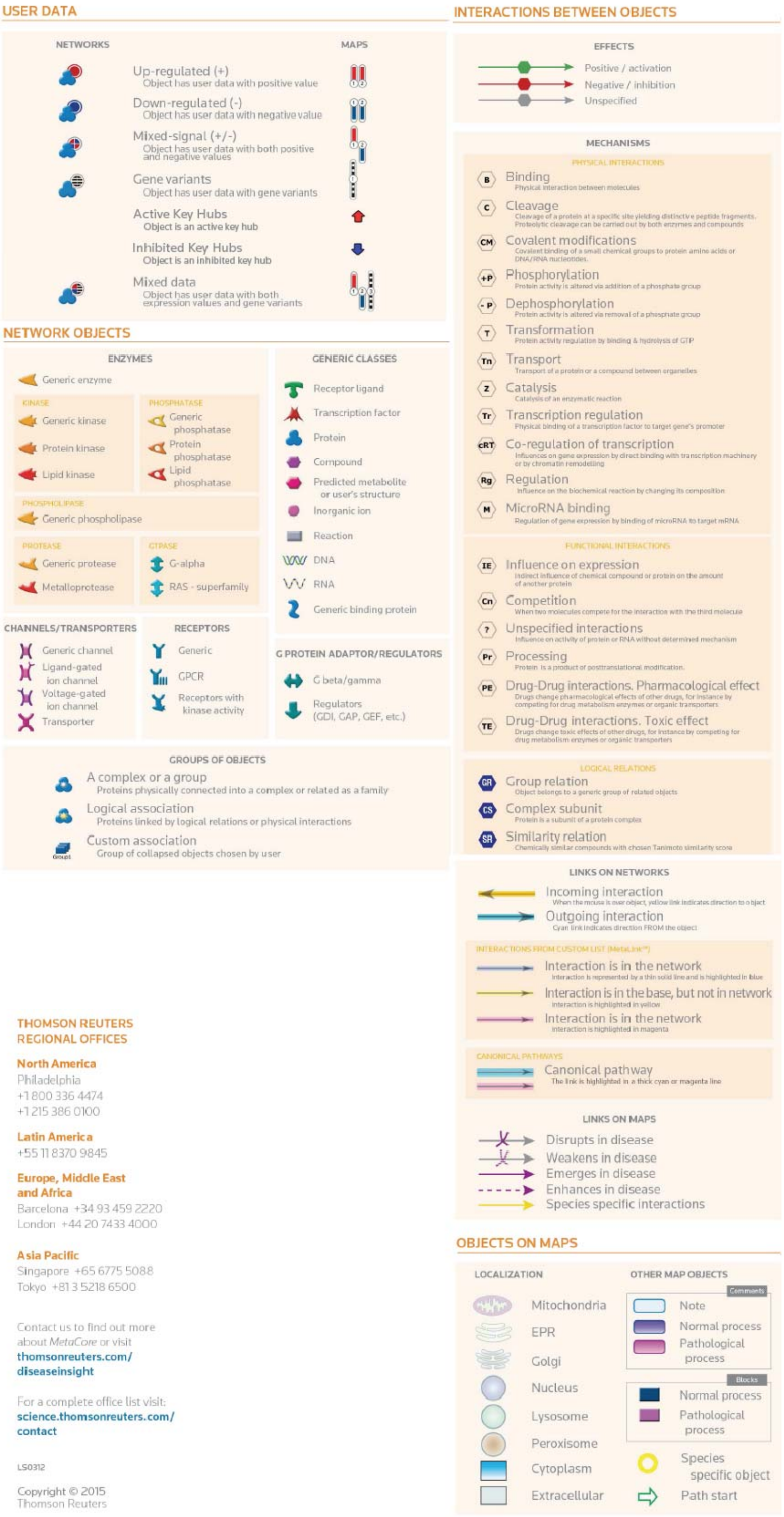

Appendix 2: Glossary

Processes Networks
A recognized series of events (interactions or biochemical reactions) accomplished by one or more ordered assemblies of molecular functions with a defined beginning and end.
Diseases (by Biomarkers)
This ontology is created based on the classification in Medical Subject Headings (MeSH). Each disease in diseases ontology has its corresponding biomarker gene or set of genes.
Disease Biomarker Networks
Manually created network models of diseases with disease biomarkers as seed nodes. The networks are organized in a folder tree. Each folder contains one or more networks for a disease. The name of the folder is the name of the disease.
Drug Target Networks (Drug Action Mechanisms)
Manually created networks with drug targets as seed nodes. These processes are derived from Processes networks that are targetable processes with genes that function coordinately when treated with drug.
Enrichment Analysis (EA) (also, Ontology Enrichment)
An analysis procedure that consists of mapping gene IDs of the dataset(s) of interest onto gene IDs in processes (terms) of built-in functional ontologies such as pathway maps, networks,diseases, etc. The terms in a given ontology are ranked based on “relevance” in the dataset. The statistical relevance procedure, a p-value of hypergeometric distribution, is calculated as the probability of a match to occur by chance, given the size of the ontology, the dataset and the particular process. The lower the p-value, the higher is the 'non-randomness' of finding the intersection between the dataset and the particular ontology term. That, in turn, translates into a higher ranking for the process matched. Everything equal, the more genes/proteins belong to a process/pathway, the lower the p-value. In EA we use multiple proprietary ontologies (canonical pathway maps, cellular processes, toxicities, disease biomarkers etc., and public ontologies such as Gene Ontology (cellular processes, protein functions, localizations).
Enrichment Synergy
The enrichment synergy method was offered for comparison of datasets that are functionally relevant but poorly overlapping at the gene level, for instance mutated and amplified genes in breast cancer [5]. The genes derived from different datasets may populate the very same pathway or process, which suggests that they are functionally complimentary. To determine whether two distinct gene lists cooperatively alter a certain cellular pathway or process, we calculate the synergy between them by ontology enrichment. An ontology term (pathway or process) is considered synergistic if the enrichment p-value for the non-redundant union of compared gene lists is lower than p-values for individual lists. More significant enrichment for the union reflects functional connectivity of two gene lists and their complementary effect on the pathway.
Process
An element or an term in an ontology, e.g., a given disease, or a given process, etc.
GO Localizations
A GO ontology for localization of the gene products inside or outside the cell. A given molecule in a given localization is represented by a network object in MetaCore™.
GO Molecular Functions
A GO ontology of hierarchically structured molecular functions. A protein may be linked to several different molecular functions.
GO Processes
A GO ontology for biological processes. The processes are structured as hierarchical tree with branches defined according to the Gene Ontology controlled vocabulary. GO process folders are nested, i.e., each folder references all the proteins participating in its sub-processes.

Key Hub (KH)	A topologically significant network object that supposed to regulate differential expression genes. KHs could be obtained by two approaches: causal reasoning network analysis and overconnectivity analysis. Using causal resoning the one could define one step KHs (transcriptional factors that statistically significant associated with experimental differential expressed genes regulation) and distant KHs (second step objects regulate one step transcriptional factors, etc, up to four steps). Overconnectivity analysis gives network objects that are overconnected with experimental differentially expressed genes.
Key Process	An ontology term (i.e. pathway maps) that enriched with both differentially expressed genes and corresponding key Hubs (see Introduction part for detailed workflow description).
Map Folder	This is a collection of manually created pathway maps, grouped hierarchically into folders according to main biological processes. Click on the folder name to open the folder in the new window and see maps.
Metabolic Networks	Metabolic Networks represent a reconstruction of metabolic processes.
Network Object	A process that describes the type of molecule, e.g. kinases, transcriptional factors, receptors, etc.
Ontology	Functional ontologies developed for biological processes, toxic processes, disease biomarkers, diseases, drug targets and drug action mechanisms. Each ontology has hierarchical tree structure and each has corresponding sets of pre-built networks and pathway maps, or, in case of disease biomarkers, gene lists.
Pathway Map	Pathway maps are graphic images representing complete biochemical pathways or signaling cascades in a commonly accepted sense. They are drawn by experts using Pathway Map Creator™ tool. Typically, a map comprises 3-5 MetaCore™ pathways. Maps are assembled into map folders divided onto regulatory, metabolic, disease, toxicity and drug action sections, and thus form an ontology of their own kind. Maps are interactive and hyperlinked to annotation pages for all objects displayed on them (genes, proteins, compounds and interactions).
Toxicity Networks	Toxicity networks are models of toxicity-related processes.

Appendix 3: List of Key Hubs IDs

For uploaded DEG lists, a causal reasoning test was performed to identify statistically significant network objects (p-value < 0.01). These proteins can be considered as topologically significant direct and indirect upstream regulators of the input genes (up to four steps from the DEG subset). First step regulators always are transcriptional factors while the other more distant regulators could be different regulatory proteins (Number of step from a significant regulator to DEG subset is defined in Distance column).

Key Hubs - Causal Reasoning

#	Network Object	Molecular Function	Object Activity	Correct/All predictions	p-value ▲	Distance
1	CDC23	Generic enzyme	-	19/21	0.0001106	3
2	NOTCH1 (NICD)	Transcription factor	+	37/48	0.0001111	2
3	RhoE	RAS superfamily	+	37/48	0.0001111	3
4	FRYL	Generic binding protein	+	37/48	0.0001111	3
5	ESCO2	Generic enzyme	-	37/48	0.0001111	3
6	miR-362-3p	RNA	-	13/13	0.0001221	2
7	MJD (ataxin-3)	Generic enzyme	+	13/13	0.0001221	3
8	miR-223-5p	RNA	-	13/13	0.0001221	2
9	ABT1	Generic binding protein	+	13/13	0.0001221	3
10	TAF1L	Protein kinase	+	13/13	0.0001221	3
11	FAP-1	Generic phosphatase	-	13/13	0.0001221	3
12	microRNA 134	RNA	-	13/13	0.0001221	2
13	TBP	Transcription factor	+	13/13	0.0001221	2
14	NR2C1 (TR2)	Transcription factor	-	33/42	0.0001358	3
15	GATA-1	Transcription factor	-	33/42	0.0001358	2
16	KLEIP	Generic binding protein	+	33/42	0.0001358	3
17	miR-195-5p	RNA	-	16/17	0.0001373	2
18	TRPS1	Transcription factor	-	16/17	0.0001373	2
19	miR-139-5p	RNA	-	16/17	0.0001373	2
20	DNMT3B	Generic enzyme	-	21/24	0.0001386	2
21	SrCap	Generic binding protein	+	21/24	0.0001386	2
22	CENP-C	Generic binding protein	-	21/24	0.0001386	3
23	HOXB3	Transcription factor	-	21/24	0.0001386	3
24	ZNF161	Transcription factor	-	21/24	0.0001386	3
25	Homer 2	Generic binding protein	-	31/39	0.000147	3
26	NFATC2IP	Generic binding protein	+	31/39	0.000147	3
27	miR-657	RNA	+	31/39	0.000147	3

Key Hubs - Causal Reasoning

#	Network Object	Molecular Function	Object Activity	Correct/All predictions	p-value ▲	Distance
28	IRF2BP2	Generic binding protein	-	31/39	0.000147	3
29	VRK2	Protein kinase	+	31/39	0.000147	3
30	NF-AT1(NFATC2)	Transcription factor	+	31/39	0.000147	2
31	IRF1	Transcription factor	+	23/27	0.0001554	2
32	STAT1/STAT2	Transcription factor	+	23/27	0.0001554	3
33	miR-608	RNA	-	29/36	0.0001563	3
34	Substance P receptor	GPCR	+	27/33	0.000162	3
35	EDNRA	GPCR	+	27/33	0.000162	3
36	Beta-arrestin1	Generic binding protein	+	27/33	0.000162	2
37	RBBP7 (RbAp46)	Generic binding protein	-	34/44	0.0001941	3
38	HMGB1	Transcription factor	+	18/20	0.0002012	2
39	miR-325-5p	RNA	-	18/20	0.0002012	3
40	miR-130b-3p	RNA	-	18/20	0.0002012	2
41	CUX1 (p110)	Transcription factor	+	18/20	0.0002012	2
42	RAG1	Generic enzyme	+	18/20	0.0002012	3
43	DPP4	Generic protease	-	18/20	0.0002012	3
44	LEDGF/p52	Generic binding protein	+	32/41	0.0002154	3
45	AAK1	Protein kinase	+	32/41	0.0002154	3
46	MAML2	Generic binding protein	+	37/49	0.0002349	3
47	MAML3	Generic binding protein	+	37/49	0.0002349	3
48	JMJD2B	Generic enzyme	+	30/38	0.000236	2
49	NPDC1	Generic protein	-	12/12	0.0002441	2
50	microRNA 548d-2	RNA	-	12/12	0.0002441	3
51	E2F6	Transcription factor	-	12/12	0.0002441	2
52	PELO	Generic binding protein	-	12/12	0.0002441	3
53	TCOF1	Transporter	+	12/12	0.0002441	3
54	HOXC9	Transcription factor	-	12/12	0.0002441	3
55	Keratin 19	Generic binding protein	+	12/12	0.0002441	3
56	Lingo1	Generic protein	-	12/12	0.0002441	3
57	miR-552-3p	RNA	-	12/12	0.0002441	3
58	BCMP84	Generic binding protein	+	12/12	0.0002441	3
59	BRD2	Protein kinase	+	12/12	0.0002441	2
60	ErbB2	Receptor with enzyme activity	+	12/12	0.0002441	2

Key Hubs - Causal Reasoning

#	Network Object	Molecular Function	Object Activity	Correct/All predictions	p-value ▲	Distance
61	GPI	Receptor ligand	+	12/12	0.0002441	3
62	miR-151-3p	RNA	+	12/12	0.0002441	3
63	Cyclin A1	Generic binding protein	+	20/23	0.0002441	2
64	microRNA 548d-1	RNA	-	12/12	0.0002441	3
65	MEF2A	Transcription factor	+	12/12	0.0002441	2
66	MSG1	Transcription factor	+	12/12	0.0002441	3
67	SH3TC2	Generic binding protein	+	12/12	0.0002441	3
68	RECK	Generic binding protein	-	12/12	0.0002441	3
69	CSF2RB	Generic receptor	+	28/35	0.0002541	3
70	TEF-3	Transcription factor	+	15/16	0.0002594	2
71	Elongin C	Generic protein	-	15/16	0.0002594	3
72	GABP alpha	Transcription factor	+	22/26	0.0002668	2
73	BORIS	Transcription factor	+	26/32	0.0002675	2
74	KLF13	Transcription factor	-	31/40	0.0003398	3
75	Cyclin T2	Generic binding protein	+	31/40	0.0003398	3
76	c-Myb	Transcription factor	+	31/40	0.0003398	2
77	miR-329-3p	RNA	-	17/19	0.0003643	2
78	AOF1	Generic binding protein	+	39/53	0.0004012	3
79	L-selectin	Generic receptor	+	27/34	0.0004107	3
80	miR-320-3p	RNA	-	19/22	0.0004277	2
81	ErbB4(ICD)	Generic binding protein	+	19/22	0.0004277	2
82	COP1	Generic enzyme	-	19/22	0.0004277	2
83	ZNF282	Transcription factor	+	19/22	0.0004277	2
84	RAD9A	Generic binding protein	+	25/31	0.000439	3
85	DDX3X	Generic enzyme	+	21/25	0.0004553	2
86	c-FLIP (S)	Generic binding protein	-	21/25	0.0004553	3
87	Histone H2AX	Generic binding protein	+	11/11	0.0004883	3
88	Nibrin	Generic binding protein	+	11/11	0.0004883	2
89	Pleiotrophin (OSF1)	Receptor ligand	-	11/11	0.0004883	3
90	miR-493-3p	RNA	-	11/11	0.0004883	2
91	CD44	Generic receptor	+	11/11	0.0004883	2
92	miR-149-3p	RNA	-	11/11	0.0004883	2
93	MAPK8/9	Generic kinase	+	11/11	0.0004883	2

Key Hubs - Causal Reasoning						
#	Network Object	Molecular Function	Object Activity	Correct/All predictions	p-value ▲	Distance
94	miR-576-3p	RNA	-	11/11	0.0004883	2
95	EBBP	Generic binding protein	-	11/11	0.0004883	2
96	miR-500-5p	RNA	-	11/11	0.0004883	3
97	DRIP130	Generic binding protein	+	14/15	0.0004883	2
98	SP4	Transcription factor	+	11/11	0.0004883	2
99	ACM3	GPCR	+	11/11	0.0004883	3
100	miR-512-3p	RNA	-	11/11	0.0004883	3
101	ANAPC5	Generic enzyme	-	11/11	0.0004883	2
102	ZNF537	Transcription factor	-	11/11	0.0004883	2
103	miR-330-3p	RNA	-	11/11	0.0004883	2
104	Birc6	Generic binding protein	+	11/11	0.0004883	3
105	miR-193a-3p	RNA	-	11/11	0.0004883	2
106	HCF1	Transcription factor	+	11/11	0.0004883	2
107	CDK5R1 (p35)	Generic binding protein	-	11/11	0.0004883	2
108	p38beta (MAPK11)	Protein kinase	+	14/15	0.0004883	2
109	TFDP3	Transcription factor	-	11/11	0.0004883	2
110	HIF-prolyl hydroxylase	Generic binding protein	-	11/11	0.0004883	2
111	IL-15 receptor	Generic receptor	+	11/11	0.0004883	3
112	JAK3	Protein kinase	+	11/11	0.0004883	2
113	MSN (moesin)	Generic binding protein	+	11/11	0.0004883	3
114	Osteopontin	Receptor ligand	+	11/11	0.0004883	3
115	Versican	Generic binding protein	+	11/11	0.0004883	3
116	microRNA 373	RNA	-	11/11	0.0004883	3
117	miR-603	RNA	-	11/11	0.0004883	2
118	E2F1	Transcription factor	+	11/11	0.0004883	1
119	Fibronectin	Receptor ligand	+	11/11	0.0004883	3
120	SFRS2 (SC-35)	Generic binding protein	+	11/11	0.0004883	2
121	microRNA 520c	RNA	-	11/11	0.0004883	3
122	GSC	Transcription factor	-	11/11	0.0004883	2
123	EVX-1	Transcription factor	+	11/11	0.0004883	3
124	UBPY	Generic protease	+	11/11	0.0004883	2
125	Den1	Generic protease	+	11/11	0.0004883	2
126	Aggrecan	Generic binding protein	+	11/11	0.0004883	3

Key Hubs - Causal Reasoning						
#	Network Object	Molecular Function	Object Activity	Correct/All predictions	p-value ▲	Distance
127	CCL5	Receptor ligand	+	11/11	0.0004883	3
128	IL-21 receptor	Generic receptor	+	11/11	0.0004883	3
129	BTG3	Generic binding protein	-	11/11	0.0004883	2
130	microRNA 32	RNA	-	11/11	0.0004883	2
131	HIC1	Transcription factor	-	30/39	0.0005325	3
132	microRNA 34b	RNA	-	28/36	0.0005966	3
133	Sec6	Generic protein	+	28/36	0.0005966	3
134	JAB1	Generic enzyme	+	28/36	0.0005966	2
135	ANAPC7	Generic binding protein	-	16/18	0.0006561	3
136	ANAPC2	Generic enzyme	-	16/18	0.0006561	3
137	ANAPC10	Generic binding protein	-	16/18	0.0006561	3
138	ANAPC1	Generic binding protein	-	16/18	0.0006561	3
139	HOTTIP	RNA	+	26/33	0.0006594	3
140	PIWIL4	Generic binding protein	+	26/33	0.0006594	3
141	WDR5	Generic binding protein	+	26/33	0.0006594	2
142	miR-214-5p	RNA	-	31/41	0.0007252	3
143	Tau (MAPT)	Generic binding protein	+	31/41	0.0007252	3
144	HSPB2	Generic binding protein	+	31/41	0.0007252	3
145	DET1	Generic protein	-	18/21	0.0007448	3
146	Thrombin	Generic protease	-	18/21	0.0007448	3
147	RGS17	Regulators (GDI, GAP, GEF etc.)	+	18/21	0.0007448	3
148	CD44 (ICD)	Generic binding protein	+	22/27	0.0007569	2
149	IL-34	Receptor ligand	+	20/24	0.0007719	3
150	RNF4	Generic enzyme	-	20/24	0.0007719	2
151	CSF1	Receptor ligand	+	20/24	0.0007719	3
152	M-CSF receptor	Generic receptor	+	20/24	0.0007719	2
153	NAB2	Transcription factor	-	37/51	0.0008846	3
154	miR-124-3p	RNA	-	37/51	0.0008846	3
155	EDF1	Transcription factor	+	13/14	0.0009155	2
156	miR-433-3p	RNA	-	13/14	0.0009155	2
157	TFIIIB90	Transcription factor	+	13/14	0.0009155	3
158	BCDIN3D	Generic enzyme	+	13/14	0.0009155	3
159	LH receptor	GPCR	-	27/35	0.0009391	3

Key Hubs - Causal Reasoning

#	Network Object	Molecular Function	Object Activity	Correct/All predictions	p-value ▲	Distance
160	c-Rel (NF-kB subunit)	Transcription factor	+	32/43	0.000957	2
161	CD28	Generic receptor	+	10/10	0.0009766	3
162	CYLD	Generic protease	+	10/10	0.0009766	2
163	microRNA 23b	RNA	-	10/10	0.0009766	2
164	JIK	Protein kinase	+	10/10	0.0009766	3
165	USP9X	Generic protease	+	10/10	0.0009766	2
166	c-IAP1	Generic binding protein	+	10/10	0.0009766	2
167	TWEAK(TNFSF12)	Receptor ligand	-	10/10	0.0009766	3
168	HGF	Receptor ligand	+	10/10	0.0009766	3
169	RFX1	Transcription factor	-	10/10	0.0009766	2
170	L1CAM	Generic binding protein	+	10/10	0.0009766	3
171	miR-362-5p	RNA	-	10/10	0.0009766	3
172	RHAMM	Generic receptor	+	10/10	0.0009766	2
173	PDEF	Transcription factor	-	10/10	0.0009766	2
174	Apo-2L(TNFSF10)	Receptor ligand	+	10/10	0.0009766	3
175	DNMT3A	Generic enzyme	-	25/32	0.001051	2
176	SALL3	Transcription factor	+	25/32	0.001051	3
177	AF-9	Generic binding protein	+	30/40	0.001111	3
178	BAF	Generic enzyme	+	15/17	0.001175	3
179	C/EBPgamma	Transcription factor	+	33/45	0.001229	3
180	ARX	Transcription factor	+	21/26	0.001247	3
181	ZBTB2	Transcription factor	-	21/26	0.001247	2
182	miR-9-3p	RNA	-	21/26	0.001247	2
183	KLF15	Transcription factor	+	17/20	0.001288	2
184	CDK3	Protein kinase	+	17/20	0.001288	2
185	Ebp1	Generic protein	-	19/23	0.0013	2
186	SMAR1	Generic binding protein	-	19/23	0.0013	2
187	miR-203-3p	RNA	-	26/34	0.001468	2
188	ETS	Transcription factor	-	29/39	0.001689	3
189	Ephrin-A receptor 4	Generic receptor	+	29/39	0.001689	3
190	miR-511-5p	RNA	+	29/39	0.001689	3
191	miR-338-3p	RNA	-	12/13	0.001709	2
192	CtBP2	Generic binding protein	-	22/28	0.00186	2
193	microRNA 9-1	RNA	-	22/28	0.00186	2
194	PKC	Protein kinase	+	9/9	0.001953	2

#	Network Object	Molecular Function	Object Activity	Correct/All predictions	p-value ▲	Distance
195	MACC1	Generic protein	+	9/9	0.001953	3
196	ERP5	Generic enzyme	+	9/9	0.001953	3
197	Lamin B2	Generic binding protein	+	9/9	0.001953	3
198	KCTD11	Voltage-gated ion-channel	+	9/9	0.001953	2
199	eIF3S9	Generic binding protein	+	9/9	0.001953	3
200	miR-1291	RNA	-	9/9	0.001953	3
201	DDX21	Generic enzyme	+	9/9	0.001953	2
202	miR-1470	RNA	-	9/9	0.001953	2
203	eIF3S4	Generic binding protein	+	9/9	0.001953	3
204	HDAC1	Generic enzyme	-	9/9	0.001953	1
205	LECT2	Receptor ligand	-	9/9	0.001953	3
206	miR-7515	RNA	-	9/9	0.001953	3
207	M9	Generic binding protein	+	9/9	0.001953	3
208	Ephrin-A receptor 7	Generic receptor	+	9/9	0.001953	3
209	eIF3S3	Generic binding protein	+	9/9	0.001953	3
210	SAMSN1	Generic binding protein	-	9/9	0.001953	2
211	eIF3S8	Generic binding protein	+	9/9	0.001953	3
212	Filamin-A (CTF)	Generic binding protein	+	9/9	0.001953	3
213	DND1	Generic binding protein	+	9/9	0.001953	2
214	eIF3	Generic protein	+	9/9	0.001953	2
215	microRNA 449a	RNA	+	9/9	0.001953	2
216	BATF2	Transcription factor	-	9/9	0.001953	2
217	Lamin B	Generic binding protein	+	9/9	0.001953	2
218	CD137(TNFRSF9)	Generic receptor	+	9/9	0.001953	3
219	MKK7 (MAP2K7)	Protein kinase	+	9/9	0.001953	2
220	HGF receptor (Met)	Receptor with enzyme activity	+	9/9	0.001953	2
221	ZPK(MAP3K12)	Protein kinase	+	9/9	0.001953	3
222	LZK(MAP3K13)	Protein kinase	+	9/9	0.001953	3
223	RNF13	Generic binding protein	+	9/9	0.001953	3
224	G-protein alpha-12 family	G-alpha	+	9/9	0.001953	3
225	HINT	Generic enzyme	-	9/9	0.001953	2

Key Hubs - Causal Reasoning (table title)

Key Hubs - Causal Reasoning						
#	Network Object	Molecular Function	Object Activity	Correct/All predictions	p-value ▲	Distance
226	eIF3S1	Generic binding protein	+	9/9	0.001953	3
227	LBC	Regulators (GDI, GAP, GEF etc.)	+	9/9	0.001953	2
228	CDK5R1 (p25)	Generic binding protein	+	9/9	0.001953	2
229	miR-139-3p	RNA	-	9/9	0.001953	2
230	70Z-PEP	Protein phosphatase	-	9/9	0.001953	3
231	miR-520h	RNA	+	9/9	0.001953	2
232	eIF3S10	Generic binding protein	+	9/9	0.001953	3
233	KCTD21	Voltage-gated ion-channel	+	9/9	0.001953	2
234	miR-513a-3p	RNA	+	9/9	0.001953	3
235	GA17	Generic binding protein	+	9/9	0.001953	3
236	HMG20A	Transcription factor	+	9/9	0.001953	3
237	PLGF	Receptor ligand	+	9/9	0.001953	3
238	eIF3S7	Generic binding protein	+	9/9	0.001953	3
239	HMG20B	Transcription factor	-	9/9	0.001953	2
240	VEGF-B	Receptor ligand	+	9/9	0.001953	3
241	eIF3S2	Generic binding protein	+	9/9	0.001953	3
242	Bak	Generic binding protein	+	9/9	0.001953	3
243	RNF187	Generic binding protein	+	9/9	0.001953	2
244	Bax	Generic binding protein	+	9/9	0.001953	3
245	c-Jun	Transcription factor	+	9/9	0.001953	1
246	IRE1	Protein kinase	+	9/9	0.001953	2
247	DACT1	Generic binding protein	-	9/9	0.001953	2
248	p66alpha	Generic binding protein	-	27/36	0.001967	3
249	KLF12	Transcription factor	-	27/36	0.001967	3
250	CtBP1	Generic binding protein	-	27/36	0.001967	2
251	DDX25	Generic enzyme	-	20/25	0.002039	3
252	GSPT1	Generic binding protein	+	20/25	0.002039	3
253	RBM9	Generic binding protein	+	20/25	0.002039	3
254	CDC26	Generic enzyme	-	14/16	0.00209	3
255	APC/hCDH1 complex	Generic enzyme	-	14/16	0.00209	2

Key Hubs - Causal Reasoning						
#	Network Object	Molecular Function	Object Activity	Correct/All predictions	p-value ▲	Distance
256	SOCS6	Generic binding protein	-	14/16	0.00209	3
257	CDC16	Generic binding protein	-	14/16	0.00209	3
258	Karyopherin alpha 3	Transporter	+	14/16	0.00209	3
259	ANAPC11	Generic enzyme	-	14/16	0.00209	3
260	ANAPC13	Generic enzyme	-	14/16	0.00209	3
261	ANAPC4	Generic enzyme	-	14/16	0.00209	3
262	microRNA 339	RNA	-	30/41	0.002162	3
263	microRNA 148a	RNA	-	30/41	0.002162	3
264	JMJD3	Generic enzyme	+	30/41	0.002162	2
265	JDP2	Transcription factor	-	18/22	0.002172	2
266	TRIM17	Generic binding protein	-	16/19	0.002213	3
267	PABP2	Generic binding protein	-	23/30	0.002611	3
268	LXR-alpha/RXR-alpha	Transcription factor	+	23/30	0.002611	3
269	miR-433-3p	RNA	-	31/43	0.002701	3
270	KIBRA	Generic binding protein	+	34/48	0.002758	3
271	NR2E3	Transcription factor	+	21/27	0.002962	2
272	miR-498	RNA	-	21/27	0.002962	3
273	PI3K reg class IA (p85)	Generic binding protein	+	26/35	0.002994	3
274	miR-18b-5p	RNA	-	11/12	0.003174	2
275	AMPK gamma subunit	Generic binding protein	+	11/12	0.003174	3
276	microRNA 31	RNA	-	11/12	0.003174	2
277	PHF8	Generic protein	+	11/12	0.003174	2
278	miR-136-5p	RNA	-	11/12	0.003174	2
279	AMPK alpha subunit	Protein kinase	+	11/12	0.003174	2
280	STAT4	Transcription factor	+	11/12	0.003174	2
281	SFRS12	Generic binding protein	-	11/12	0.003174	3
282	IL-12RB2	Generic receptor	+	11/12	0.003174	3
283	Flotillin-1	Generic binding protein	+	19/24	0.003305	3
284	JNK2(MAPK9)	Protein kinase	+	19/24	0.003305	2
285	miR-378-5p	RNA	-	24/32	0.0035	3
286	UCHL1	Generic enzyme	+	17/21	0.003599	2
287	miR-922	RNA	-	17/21	0.003599	3
288	RNPC1	Generic binding protein	-	13/15	0.003693	2

Key Hubs - Causal Reasoning						
#	Network Object	Molecular Function	Object Activity	Correct/All predictions	p-value ▲	Distance
289	c-Rel (NF-kB subunit)	Transcription factor	+	36/52	0.003894	3
290	CCL13	Receptor ligand	+	8/8	0.003906	3
291	CCL2	Receptor ligand	+	8/8	0.003906	3
292	Plectin 1	Generic binding protein	+	8/8	0.003906	3
293	CXCR4	GPCR	+	8/8	0.003906	2
294	C/EBPgamma	Transcription factor	+	8/8	0.003906	2
295	CXCL14	Receptor ligand	-	8/8	0.003906	3
296	CDC27	Generic binding protein	-	8/8	0.003906	2
297	HP	Receptor ligand	+	8/8	0.003906	3
298	Drebrin	Generic binding protein	+	8/8	0.003906	3
299	VDAC 1	Voltage-gated ion-channel	+	8/8	0.003906	3
300	MYH9	Generic binding protein	+	30/42	0.003958	3
301	Ku80	Generic binding protein	+	30/42	0.003958	3
302	TDG	Generic enzyme	+	25/34	0.004521	2
303	ADAR1	Generic enzyme	-	25/34	0.004521	3
304	microRNA 9-2	RNA	-	20/26	0.004678	2
305	Cathepsin S	Generic protease	+	20/26	0.004678	3
306	Caspase-10	Generic protease	-	34/49	0.0047	3
307	LRRC42	Generic binding protein	-	18/23	0.005311	3
308	DEC2	Transcription factor	-	18/23	0.005311	2
309	RPRD1B	Generic protein	+	18/23	0.005311	2
310	SENP5	Generic protease	+	18/23	0.005311	3
311	BIN1 (Amphiphysin II)	Generic binding protein	-	18/23	0.005311	2
312	MBD3	Generic binding protein	-	18/23	0.005311	2
313	Elk-4	Transcription factor	+	18/23	0.005311	2
314	CDON	Generic binding protein	+	23/31	0.005337	3
315	Taspase1	Generic protease	+	26/36	0.005665	3
316	FADD	Generic binding protein	-	26/36	0.005665	3
317	MLL1 (HRX)	Transcription factor	+	26/36	0.005665	2
318	QKI	Generic binding protein	-	10/11	0.005859	2
319	E2F8	Generic protein	-	10/11	0.005859	2
320	WNK1	Protein kinase	+	10/11	0.005859	3

Key Hubs - Causal Reasoning						
#	Network Object	Molecular Function	Object Activity	Correct/All predictions	p-value ▲	Distance
321	KLF8	Transcription factor	+	10/11	0.005859	2
322	PAX8	Transcription factor	+	10/11	0.005859	2
323	HEY2	Transcription factor	-	10/11	0.005859	2
324	PRR6	Generic enzyme	+	16/20	0.005909	3
325	NF-I	Transcription factor	+	16/20	0.005909	2
326	TARBP2	Generic binding protein	-	16/20	0.005909	3
327	DR4(TNFRSF10A)	Receptor with enzyme activity	-	16/20	0.005909	3
328	Fc alpha receptor	Generic receptor	+	16/20	0.005909	3
329	CD79 complex	Generic receptor	+	16/20	0.005909	3
330	PSTPIP2	Generic binding protein	+	16/20	0.005909	3
331	SPHK1	Lipid kinase	+	16/20	0.005909	3
332	Lyn	Protein kinase	+	16/20	0.005909	2
333	Fc gamma RI	Generic receptor	+	16/20	0.005909	3
334	EFHD2	Generic binding protein	+	16/20	0.005909	3
335	Hemogen	Generic protein	-	21/28	0.00627	3
336	PES1	Generic binding protein	+	12/14	0.00647	2
337	BOP1	Generic binding protein	+	12/14	0.00647	3
338	MAK	Protein kinase	+	12/14	0.00647	3
339	GRB14	Generic binding protein	-	12/14	0.00647	3
340	CDH1	Generic binding protein	-	12/14	0.00647	2
341	miR-1285-3p	RNA	-	33/48	0.006642	3
342	miR-135b-5p	RNA	-	19/25	0.007317	2
343	miR-31-5p	RNA	-	19/25	0.007317	2
344	miR-644a	RNA	-	19/25	0.007317	3
345	HSPA1L	Generic binding protein	-	7/7	0.007813	3
346	NudE	Generic binding protein	+	7/7	0.007813	3
347	HUC	Generic binding protein	+	7/7	0.007813	2
348	Parathymosin	Generic binding protein	+	7/7	0.007813	2
349	HDAC2	Generic enzyme	-	7/7	0.007813	1
350	PMF1	Generic binding protein	-	7/7	0.007813	2
351	ZNF378	Generic enzyme	+	7/7	0.007813	3

#	Network Object	Molecular Function	Object Activity	Correct/All predictions	p-value ▲	Distance
Key Hubs - Causal Reasoning						
352	HSPA6	Generic binding protein	-	7/7	0.007813	3
353	GCR-beta	Transcription factor	+	7/7	0.007813	2
354	Importin 13	Transporter	+	7/7	0.007813	2
355	BTG1	Generic binding protein	+	7/7	0.007813	2
356	Zdhhc2	Generic enzyme	+	7/7	0.007813	3
357	Connexin 37	Generic channel	-	7/7	0.007813	3
358	FOXO3A	Transcription factor	+	7/7	0.007813	1
359	HSP70	Generic binding protein	-	7/7	0.007813	2
360	UBCH7	Generic enzyme	-	7/7	0.007813	2
361	RFX5	Transcription factor	-	7/7	0.007813	2
362	CACNA1G	Voltage-gated ion-channel	+	7/7	0.007813	3
363	TRAP80	Generic binding protein	+	7/7	0.007813	2
364	Relaxin 2	Receptor ligand	+	7/7	0.007813	2
365	GCR-alpha	Transcription factor	+	7/7	0.007813	1
366	TEF-5	Transcription factor	+	7/7	0.007813	2
367	Ku70/80	Generic binding protein	+	7/7	0.007813	2
368	CRP	Generic binding protein	-	7/7	0.007813	3
369	NRIP	Generic binding protein	+	7/7	0.007813	2
370	AP-2	Transcription factor	-	7/7	0.007813	2
371	NMT2	Generic enzyme	+	7/7	0.007813	3
372	FOXM1	Transcription factor	+	7/7	0.007813	1
373	MEIS2	Transcription factor	+	7/7	0.007813	2
374	HSPA2	Generic binding protein	-	7/7	0.007813	3
375	MAD	Transcription factor	-	7/7	0.007813	2
376	HSPA7	Generic binding protein	-	7/7	0.007813	3
377	G-protein beta-1	G beta/gamma	-	7/7	0.007813	2
378	SUMO-4	Generic enzyme	+	7/7	0.007813	2
379	NMT1	Generic enzyme	+	7/7	0.007813	3
380	TRFP	Generic binding protein	+	7/7	0.007813	2
381	STAMP (KIAA0998)	Generic enzyme	+	7/7	0.007813	2
382	PMCA1	Generic protein	-	7/7	0.007813	3
383	ZDHHC3	Generic enzyme	+	7/7	0.007813	3

Key Hubs - Causal Reasoning

#	Network Object	Molecular Function	Object Activity	Correct/All predictions	p-value ▲	Distance
384	Relaxin 1	Receptor ligand	+	7/7	0.007813	2
385	ERM	Transcription factor	+	7/7	0.007813	2
386	eNOS	Generic enzyme	+	7/7	0.007813	2
387	AP-2E	Transcription factor	-	7/7	0.007813	3
388	HMG1,2	Transcription factor	+	7/7	0.007813	2
389	G-protein beta-2	G beta/gamma	-	7/7	0.007813	2
390	miR-3127-5p	RNA	-	22/30	0.008062	3
391	MAD3	Transcription factor	-	22/30	0.008062	3
392	ARGBP2	Generic binding protein	-	22/30	0.008062	3
393	ID2	Transcription factor	-	22/30	0.008062	2
394	miR-431-5p	RNA	-	22/30	0.008062	3
395	ASB4	Generic binding protein	+	22/30	0.008062	3
396	WASF2	Generic binding protein	+	22/30	0.008062	3
397	MSH5	Generic binding protein	+	22/30	0.008062	3
398	c-Abl	Protein kinase	+	22/30	0.008062	2
399	PELP1	Transcription factor	+	28/40	0.008295	2
400	MLL2	Generic enzyme	+	25/35	0.008337	2
401	Sgsm1	Regulators (GDI, GAP, GEF etc.)	-	17/22	0.00845	3
402	miR-222-3p	RNA	-	17/22	0.00845	2
403	JunD	Transcription factor	+	17/22	0.00845	2
404	NURR1	Transcription factor	-	17/22	0.00845	2
405	COTE1	Generic binding protein	+	20/27	0.009579	3
406	Helios	Transcription factor	-	20/27	0.009579	3
407	WWOX	Generic enzyme	-	20/27	0.009579	2
408	IRS-2	Generic binding protein	+	20/27	0.009579	3
409	FOXP3	Transcription factor	-	20/27	0.009579	2
410	Survivin	Generic binding protein	-	15/19	0.009605	2
411	CIDEC	Generic protein	-	15/19	0.009605	3
412	DUS2	Generic enzyme	-	15/19	0.009605	3
413	CDCA8	Generic binding protein	-	15/19	0.009605	3
414	hnRNPH'	Generic binding protein	-	15/19	0.009605	3
415	miR-1285-3p	RNA	-	15/19	0.009605	2
416	Insulin processed	Receptor ligand	+	15/19	0.009605	3

Key Hubs - Causal Reasoning

#	Network Object	Molecular Function	Object Activity	Correct/All predictions	p-value ▲	Distance
417	eIF2S1	Generic binding protein	-	15/19	0.009605	3
418	Furin	Generic protease	+	15/19	0.009605	3
419	JNK3(MAPK10)	Protein kinase	+	15/19	0.009605	2
420	FBXL7	Generic binding protein	+	15/19	0.009605	3
421	MSP	Receptor ligand	+	15/19	0.009605	3
422	IGF-2	Receptor ligand	+	15/19	0.009605	3
423	PKR	Protein kinase	+	15/19	0.009605	2
424	NF-AT5	Transcription factor	+	15/19	0.009605	2
425	MKLP2	Generic binding protein	-	15/19	0.009605	3
426	CUGBP1	Generic binding protein	-	15/19	0.009605	2
427	Perlecan	Generic binding protein	-	15/19	0.009605	3
428	Cyclin C	Generic binding protein	+	29/42	0.00976	3
429	EAP30	Generic binding protein	-	20/21	1.049E-05	3
430	ELL	Generic binding protein	-	20/21	1.049E-05	2
431	Tubulin (in microtubules)	Generic binding protein	+	33/33	1.164E-10	3
432	E2F7	Generic protein	-	23/23	1.192E-07	2
433	HYPH	Generic enzyme	-	32/38	1.217E-05	3
434	HDBP1	Transcription factor	-	32/38	1.217E-05	3
435	Huntingtin	Generic binding protein	-	32/38	1.217E-05	2
436	MLX	Transcription factor	-	32/38	1.217E-05	3
437	miR-570-3p	RNA	-	38/43	1.25E-07	3
438	BAF155	Generic binding protein	+	25/28	1.372E-05	2
439	miR-106a-5p	RNA	-	23/24	1.49E-06	2
440	Aprataxin	Generic binding protein	+	30/31	1.49E-08	3
441	SYT	Generic binding protein	+	44/50	1.622E-08	3
442	NCOA3 (pCIP/SRC3)	Generic binding protein	+	44/50	1.622E-08	2
443	HB-EGF	Receptor ligand	+	40/48	1.653E-06	3
444	Zac1	Transcription factor	+	27/31	1.698E-05	2
445	S14 protein	Generic binding protein	+	27/31	1.698E-05	3
446	Leptin	Receptor ligand	+	29/29	1.863E-09	3

Key Hubs - Causal Reasoning

#	Network Object	Molecular Function	Object Activity	Correct/All predictions	p-value ▲	Distance
447	NudC	Generic binding protein	+	29/29	1.863E-09	3
448	HSP90 alpha	Generic binding protein	+	29/29	1.863E-09	2
449	EAR2	Transcription factor	-	39/49	1.923E-05	3
450	Galectin-3	Generic binding protein	+	19/20	2.003E-05	2
451	Annexin VII	Generic binding protein	+	19/20	2.003E-05	3
452	Elastin	Generic binding protein	+	19/20	2.003E-05	3
453	CD13	Metalloprotease	+	19/20	2.003E-05	3
454	CSPG4 (NG2)	Cell membrane glycoprotein	+	19/20	2.003E-05	3
455	TRIP11	Generic binding protein	+	33/34	2.037E-09	3
456	ARNT	Transcription factor	+	33/34	2.037E-09	2
457	ZAK	Protein kinase	+	26/27	2.086E-07	3
458	MSK1	Protein kinase	+	26/27	2.086E-07	2
459	p107	Generic binding protein	-	32/35	2.088E-07	2
460	APP	Generic receptor	+	35/43	2.097E-05	3
461	miR-22-3p	RNA	-	33/38	2.128E-06	2
462	WRCH-1	RAS superfamily	+	37/42	2.217E-07	3
463	MAP3K3	Protein kinase	+	32/32	2.328E-10	3
464	Galectin-3	Generic binding protein	+	35/41	2.437E-06	3
465	HSP90 alpha	Generic binding protein	+	45/56	2.689E-06	3
466	TTRAP	Generic binding protein	-	39/47	2.77E-06	3
467	microRNA 208a	RNA	-	39/47	2.77E-06	3
468	miR-107-3p	RNA	-	25/27	2.824E-06	2
469	Pellino 1	Generic protein	-	40/51	2.852E-05	3
470	Necdin	Generic binding protein	-	22/23	2.861E-06	2
471	Dysbindin	Generic binding protein	+	22/23	2.861E-06	3
472	ELF1	Transcription factor	+	22/23	2.861E-06	2
473	DDX6	Generic enzyme	-	15/15	3.052E-05	2
474	Lnk	Generic binding protein	-	30/34	3.082E-06	3
475	RAD1	Generic binding protein	+	36/45	3.287E-05	3
476	alpha-2/beta-1 integrin	Generic receptor	+	36/45	3.287E-05	3

Key Hubs - Causal Reasoning						
#	Network Object	Molecular Function	Object Activity	Correct/All predictions	p-value ▲	Distance
477	ETS1	Transcription factor	+	43/56	3.667E-05	3
478	miR-501-5p	RNA	-	28/28	3.725E-09	3
479	HBXIP	Generic binding protein	+	28/28	3.725E-09	2
480	microRNA 20a	RNA	-	18/19	3.815E-05	2
481	MTF-1	Transcription factor	+	18/19	3.815E-05	2
482	ATF-2/c-Jun	Transcription factor	+	18/19	3.815E-05	2
483	microRNA 106b	RNA	-	18/18	3.815E-06	2
484	miR-122-5p	RNA	-	18/18	3.815E-06	2
485	TACC3	Generic binding protein	+	32/33	3.958E-09	3
486	Beta-1 adrenergic receptor	GPCR	+	34/40	4.182E-06	3
487	GATA-1	Transcription factor	-	39/50	4.511E-05	3
488	HMG20B	Transcription factor	-	42/52	4.532E-06	3
489	ETS1	Transcription factor	+	38/46	4.624E-06	2
490	NF-AT2(NFATC1)	Transcription factor	+	38/46	4.624E-06	2
491	HB9	Transcription factor	-	38/46	4.624E-06	3
492	microRNA 17	RNA	-	21/21	4.768E-07	2
493	miR-106b-5p	RNA	-	21/21	4.768E-07	2
494	miR-223-3p	RNA	-	21/21	4.768E-07	2
495	HSP27	Generic binding protein	+	25/29	5.186E-05	2
496	E2F3	Transcription factor	+	25/29	5.186E-05	2
497	miR-124-3p	RNA	-	35/44	5.302E-05	2
498	ACK1	Protein kinase	+	35/44	5.302E-05	3
499	Neuregulin 1	Receptor ligand	+	21/22	5.484E-06	3
500	Mxi1	Transcription factor	+	28/29	5.588E-08	3
501	miR-455-5p	RNA	-	27/32	5.654E-05	3
502	DROSHA	Generic enzyme	-	31/38	5.808E-05	3
503	microRNA 132	RNA	+	31/38	5.808E-05	3
504	MeCP2	Generic binding protein	-	31/38	5.808E-05	2
505	PRMT1	Generic enzyme	+	29/35	5.842E-05	2
506	p16INK4	Generic binding protein	-	24/24	5.96E-08	2
507	SUV39H2	Generic enzyme	+	24/24	5.96E-08	3
508	Laminin 1	Receptor ligand	+	24/24	5.96E-08	3
509	SMARCA3	Transcription factor	+	24/24	5.96E-08	2
510	ZNF277	Transcription factor	+	24/24	5.96E-08	3
511	NeuroD2	Transcription factor	-	24/24	5.96E-08	3

Key Hubs - Causal Reasoning

#	Network Object	Molecular Function	Object Activity	Correct/All predictions	p-value ▲	Distance
512	ISOC2	Generic enzyme	+	24/24	5.96E-08	3
513	HLX1	Transcription factor	+	24/24	5.96E-08	3
514	microRNA 424	RNA	+	20/22	6.056E-05	3
515	HIC1	Transcription factor	-	20/22	6.056E-05	2
516	FBXL11	Generic enzyme	-	20/22	6.056E-05	2
517	ATF-2/c-Jun	Transcription factor	+	47/60	6.073E-06	3
518	EGLN2	Generic binding protein	-	14/14	6.104E-05	2
519	PRDM13	Generic binding protein	-	14/14	6.104E-05	3
520	miR-331-3p	RNA	-	14/14	6.104E-05	2
521	HOX11	Transcription factor	+	14/14	6.104E-05	3
522	JunB/Fra-1	Transcription factor	+	14/14	6.104E-05	3
523	Mucin 4	Generic binding protein	+	14/14	6.104E-05	3
524	hASH1	Transcription factor	+	14/14	6.104E-05	2
525	Flotillin-2	Generic binding protein	+	14/14	6.104E-05	3
526	RWDD3	Generic enzyme	+	14/14	6.104E-05	2
527	miR-200b-3p	RNA	-	30/33	7.006E-07	2
528	NUDEL	Generic binding protein	+	17/18	7.248E-05	3
529	Nucleolin	Generic binding protein	+	27/27	7.451E-09	2
530	miR-505-3p	RNA	-	26/29	7.618E-06	3
531	microRNA 27b	RNA	-	17/17	7.629E-06	2
532	NF45 (ILF2)	Transcription factor	+	17/17	7.629E-06	3
533	Prdm8	Generic binding protein	+	24/25	7.749E-07	3
534	Jagged1	Receptor ligand	-	36/46	7.821E-05	3
535	CIN85	Generic binding protein	-	37/43	8.181E-07	3
536	HIVEP3	Transcription factor	+	32/40	9.108E-05	3
537	SMAD2	Transcription factor	+	32/40	9.108E-05	2
538	PEAR1	Generic protein	+	20/20	9.537E-07	3
539	HOXB7	Transcription factor	+	30/37	9.554E-05	3
540	Histone H1.3	Generic binding protein	-	26/31	9.61E-05	3
541	FBI-1 (Pokemon)	Transcription factor	-	26/31	9.61E-05	2
542	MDR1	Transporter	+	26/31	9.61E-05	3
543	miR-93-5p	RNA	-	28/32	9.651E-06	2
544	STAT5	Transcription factor	+	23/25	9.716E-06	2

Key Hubs - Causal Reasoning						
#	Network Object	Molecular Function	Object Activity	Correct/All predictions	p-value ▲	Distance
545	SIX4	Transcription factor	+	28/34	9.756E-05	3
546	UBR3	Generic binding protein	-	28/34	9.756E-05	3
547	APEX	Generic enzyme	+	28/34	9.756E-05	2
548	EPAS1	Transcription factor	+	28/34	9.756E-05	2
